# Pathogen-derived mechanical cues potentiate the spatio-temporal implementation of plant defense

**DOI:** 10.1186/s12915-022-01495-w

**Published:** 2022-12-27

**Authors:** Ophélie Léger, Frédérick Garcia, Mehdi Khafif, Sebastien Carrere, Nathalie Leblanc-Fournier, Aroune Duclos, Vincent Tournat, Eric Badel, Marie Didelon, Aurélie Le Ru, Sylvain Raffaele, Adelin Barbacci

**Affiliations:** 1 Université de Toulouse, INRAE, CNRS, Laboratoire des Interactions Plantes Micro-organismes Environnement (LIPME), 31326 Castanet-Tolosan, France; 2 Université de Toulouse, INRAE, Mathematiques et Informatique Appliquées de Toulouse (MIAT), 31326 Castanet-Tolosan, France; 3 Université Clermont Auvergne, INRAE, PIAF, 63000 Clermont-Ferrand, France; 4 Laboratoire d’Acoustique de l’Université du Mans (LAUM), UMR 6613, Institut d’Acoustique - Graduate School (IA-GS), CNRS, Le Mans Université, Le Mans, France; 5 Plateforme Imagerie TRI-FRAIB, Université de Toulouse, CNRS, 31326 Castanet-Tolosan, France

**Keywords:** Plant immunity, Mechanoperception, Mechanotransduction, Microtubules, Plant-pathogen interactions, Quantitative disease resistance

## Abstract

**Background:**

The ongoing adaptation of plants to their environment is the basis for their survival. In this adaptation, mechanoperception of gravity and local curvature plays a role of prime importance in finely regulating growth and ensuring a dynamic balance preventing buckling. However, the abiotic environment is not the exclusive cause of mechanical stimuli. Biotic interactions between plants and microorganisms also involve physical forces and potentially mechanoperception. Whether pathogens trigger mechanoperception in plants and the impact of mechanotransduction on the regulation of plant defense remains however elusive.

**Results:**

Here, we found that the perception of pathogen-derived mechanical cues by microtubules potentiates the spatio-temporal implementation of plant immunity to fungus. By combining biomechanics modeling and image analysis of the post-invasion stage, we reveal that fungal colonization releases plant cell wall-born tension locally, causing fluctuations of tensile stress in walls of healthy cells distant from the infection site. In healthy cells, the pathogen-derived mechanical cues guide the reorganization of mechanosensing cortical microtubules (CMT). The anisotropic patterning of CMTs is required for the regulation of immunity-related genes in distal cells. The CMT-mediated mechanotransduction of pathogen-derived cues increases *Arabidopsis* disease resistance by 40% when challenged with the fungus *Sclerotinia sclerotiorum*.

**Conclusions:**

CMT anisotropic patterning triggered by pathogen-derived mechanical cues activates the implementation of early plant defense in cells distant from the infection site. We propose that the mechano-signaling triggered immunity (MTI) complements the molecular signals involved in pattern and effector-triggered immunity.

**Supplementary Information:**

The online version contains supplementary material available at 10.1186/s12915-022-01495-w.

## Background

The immune system regulates the permanent interactions between living organisms and microorganisms of the environment. Plants are no exception to this principle, and a complex immune system emerged during evolution to support their adaptation to their biotic environment. The perception of fluctuations in the cellular environment associated with pathogens is at the heart of the immune system. Current models of plant immunity involve the perception of microbial molecules, free carbohydrates, and other host degradation products for the activation of localized defense. The PAMP-triggered immunity (PTI) is initiated by the perception of conserved molecular patterns termed PAMP (pathogen-associated molecular patterns) by cell surface and intracellular pattern recognition receptors (PRRs). Molecules designated as effectors secreted by pathogens can suppress PTI by inhibiting the perception of PAMPs. However, effector perception by intracellular plant immune receptors triggers the so-called effector-triggered immunity (ETI) that confers complete disease resistance. In nature, a partial resistance phenotype called quantitative disease resistance (QDR) is often observed [1]⁠. The QDR is intrinsically multigenic [2] involving multiple genes that may not be specific to plant defense [3]⁠. As a genetically controlled spatio-temporal process, plant immunity requires the dynamic regulation of gene expression based on signaling across plant cells and organs. The transfer of secondary messenger molecules such as calcium or reactive oxygen species is proposed to inform rapidly distant cells of the occurrence and position of a pathogen attack [4]⁠. However, signals governing plant systemic immunity remain incompletely understood [5]⁠⁠.

Once in host tissues, many plant pathogens derive carbon from the cell wall of their host for growth and reproduction. It is the case of necrotrophic fungal pathogens that secrete enzymes and organic acids to exploit carbon sources in plant cell walls [6]⁠. The active fungal cell wall polysaccharide hydrolysis modifies plant cell wall stiffness and affects the mechanical stress monitored by cells. Mechanical stress and strain tensor fields are mechanical cues read by cells and relayed by other forms of signals such as Ca2+ waves closely linked to ROS production [7]⁠. Mechanical cues are central to many aspects of plant development and adaptation. Indeed, plants are composed of fixed and bound cells that form a solid-like continuous material favorable to the spread of mechanical cues. Plant tissues are under high internal turgor pressure generated by the interaction of water in the vacuole and the stiff polysaccharides wall. In plants, the monitoring of stress fluctuations in cell walls is a central mechanism to the perception of self (proprioception) and the environment (exteroception) [8, 9]⁠.

The molecular basis of plant mechanoperception remains partial. Proteins involved in mechanoperception have been identified in different cell compartments [10, 11]⁠⁠. Membrane-bound mechanosensitive ion channels regulate ion fluxes across the membrane in response to fluctuations in tension and are widespread in plants [10]⁠. They contribute to the direct perception of mechanical cues by conformational changes of their transmembrane region. MscS-like family, homologs of bacterial mechanosensitive channels are localized to the plasma membrane, mitochondria, and plastids and are involved in the regulation of turgor pressure. Mid1-complementing activity (MCA) promotes Ca2+ fluxes whereas the mechanically gated two-pore potassium (TPK) family promotes K+ fluxes. The perception of mechanical cues can also be indirect by perceiving consequences of mechanical stimuli on cell wall status through members of transmembrane receptor-like kinases (RLKs) family such as *Catharanthus roseus* RLK (crRLKs), wall-associated kinases (WAKs). Cytoskeleton filaments are also central components of plant mechanoperception. Involved in perception and transmission, cortical microtubules (CMTs) play a double role in mechanoperception. Because of their stiffness, CMTs are a good substrate to transmit tension through tissue [12, 13]. Over the years, it has become clear that CMTs are also their own mechanosensor that reorganizes in the direction of the principal stress [14–17]⁠.

Some of these mechanosensitive proteins are involved in cell wall integrity (CWI) maintenance ensuring the functional integrity of walls [18, 19]. Interestingly, the PTI response is dependent on osmotic pressure and mechanosensitive channels [20]⁠. However, whether plant cells directly sense and respond to mechanical stimuli induced by pathogen colonization has not been formally established. This prompted us to ask whether the mechanoperception of pathogen-derived stress and strain could also act as a signal controlling immune responses in plants.

Here, we investigated the activation of plant immunity by pathogen-derived mechanical cues during post-invasion stage by combining confocal microscopy, image analysis, mechanical modeling, and molecular biology. We focused on the *Arabidopsis thaliana* quantitative disease resistance to the necrotrophic fungus *Sclerotinia sclerotiorum* [1, 21]⁠. Like other necrotrophic fungi, *S. sclerotiorum* derives the energy required for its own growth and virulence by destroying host cells. In this system, plant cell death is generally not caused by direct physical contact between pathogen and host cells but by a myriad of secreted molecules like oxalic acid and cell wall degrading enzymes [22]⁠⁠⁠. To attest to the tensorial aspect of mechanical cues encoding intensity and direction [23]⁠, cortical microtubules (CMTs) are relevant anisotropic probes. In this work, we show that the mechanoperception of pathogen-derived stress potentiates immunity in healthy cells distant from the infection site. This mechanism requires the anisotropic patterning of mechanosensitive microtubules and contributes to >40% of the disease-resistance phenotype.

## Results

### Infection by a fungal pathogen partially releases tension in distal plant cell walls

**Fig. 1:**
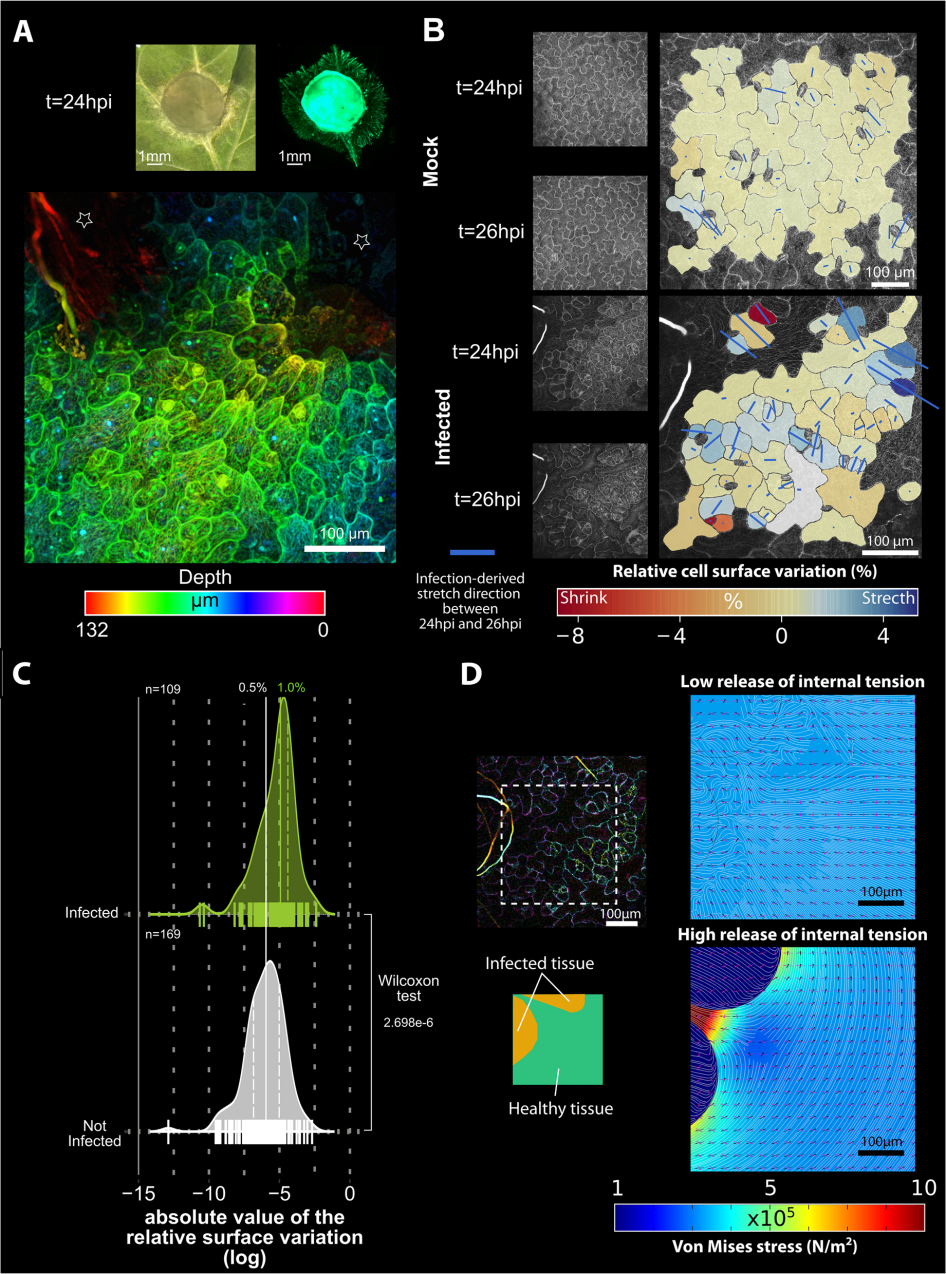
Infection by *Sclerotinia sclerotiorum* releases internal mechanical stress in *Arabidopsis thaliana* epidermal cells. **A** At 24 h post-inoculation (hpi), the fungal colony actively grew in plant tissue. The colony center was in necrotic hydrolyzed tissue (brown bright field top left) whereas the colony periphery grew in healthy tissue (fluorescence top right). Z colored-depth micrograph (bottom) showing an under-epidermal growth of *S. sclerotiorum* (stars). **B** Confocal micrograph representative of *A. thaliana* epidermal cells in uninfected tissue (top) and in the vicinity of *S. sclerotiorum* infection front, with an invasive hyphae visible as a bright thick filament in the upper left corner of the image. Overlays show the experimental analysis of pathogen-derived stretch (blue segments, scale specific to each micrograph) between 24 and 26 hpi, and cell surface variation indicated as a colored background. **C** Distribution of the magnitude of the absolute value of the relative cell surface variation triggered by pathogen-derived stress release. Individual measures are shown as bars along the *X*-axis (*n*=278 cells), the average was 1% in infected tissue and 0.5% healthy tissue. **D** Mechanical finite element model (FEM) of infection-derived stress. The model was used to simulate stress and strain in a region of interest (ROI) mimicking the leaf sector shown in **B** and at the left of the panel. Low release of internal stress and Young’s modulus (top). High release of internal stress and Young’s modulus (bottom)

To analyze the mechanical consequences of infection by the necrotrophic fungal pathogen *Sclerotinia sclerotiorum*, we monitored the 2D kinematics of *Arabidopsis* deformation in epidermal cells expressing the microtubule-associated protein 4 (MAP4) fused to GFP over 2-h time courses (24 to 26 h post-inoculation (hpi)). At 24 hpi, the fungus has already penetrated the host tissue and grows actively (Fig. 1A). In the post-invasion stage, the fungal colony is spatially self-organized. The colony center derived its resources from the hydrolyzed cells to support the growth and the virulence of the distal hyphae in contact with healthy and resistant plant cells [22]⁠. Since reshuffling of mechanical stress in walls leads to cell deformations, we combined time-lapse confocal imaging and optical flow tracking [24]⁠ to quantify cell shape variation. We found that infection by *S. sclerotiorum* generated mechanical strains in healthy and viable plant cells adjacent to the mycelium periphery (Additional file 1: Fig. S1), due to the global reorganization of mechanical stress balance in walls (Fig. 1B). Relative surface shape variations were extracted from the strain tensor and computed as the first invariant. The mean magnitude of variations was on absolute value ca. 1% of the initial surface, and twice higher in infected than in healthy tissues (Fig. 1C). The distribution of relative surface variation indicated that the infection caused deformation of all cells in the vicinity of the necrosis. In infected tissues, 50% of the cells had a relative surface variation between 0.6 and 10%. In uninfected tissue, 25% of cells only had a relative surface variation superior to 0.6%. The possible guard cell deregulation triggered by *S. sclerotiorum*-secreted oxalic acid [25]⁠ did not bias the mechanical strain fields measured between 24 hpi and 26 hpi in the vicinity of the fungal colony (Additional file 2: Fig. S2). Deformations affected all observed cells and were not limited to cells directly in contact with the pathogen (Fig. 1A). Cell surfaces were either shrunken or stretched, suggesting that the mechanical stress reshuffling necessary to reach the mechanical equilibrium resulted from the balance between local shape-derived stress and pathogen-derived stress. The pathogen-derived strain pattern was weakly tangential to the periphery of the area colonized by the fungus (Fig. 1B), in contrast with the circumferential stress pattern typically observed after laser ablation of cells [14, 15]⁠. To explore possible causes for this discrepancy, we derived a 2D mechanical model of leaf tissue at a similar scale to confocal observations. In this model, we mimicked the mechanical effect of pathogen inoculation by reducing Young’s modulus and internal stress in infected zones. The model assumed isotropic plant tissue implying that stress scaled with strain. The model showed that a low release of the initial isotropic tension led to a non-circumferential strain pattern around the infection site whereas a further release of internal stress led to the expected circumferential strain patterning observed in previous studies (Fig. 1D). During infection, the circumferential CMTs patterning suggesting the circumferential stress patterning was also observed in advanced hydrolyzed epidermal tissue stage (Additional file 3: Fig. S3). We conclude that the non-circumferential strain pattern we observed is due to the partial release of mechanical stress in intact leaf tissue, consistent with *S. sclerotiorum* causing partial cell wall loosening through the chemical activity of enzymes and organic acids [22]⁠.

### Pathogen-derived tensile stretches guides the reorganization of CMTs

**Fig. 2:**
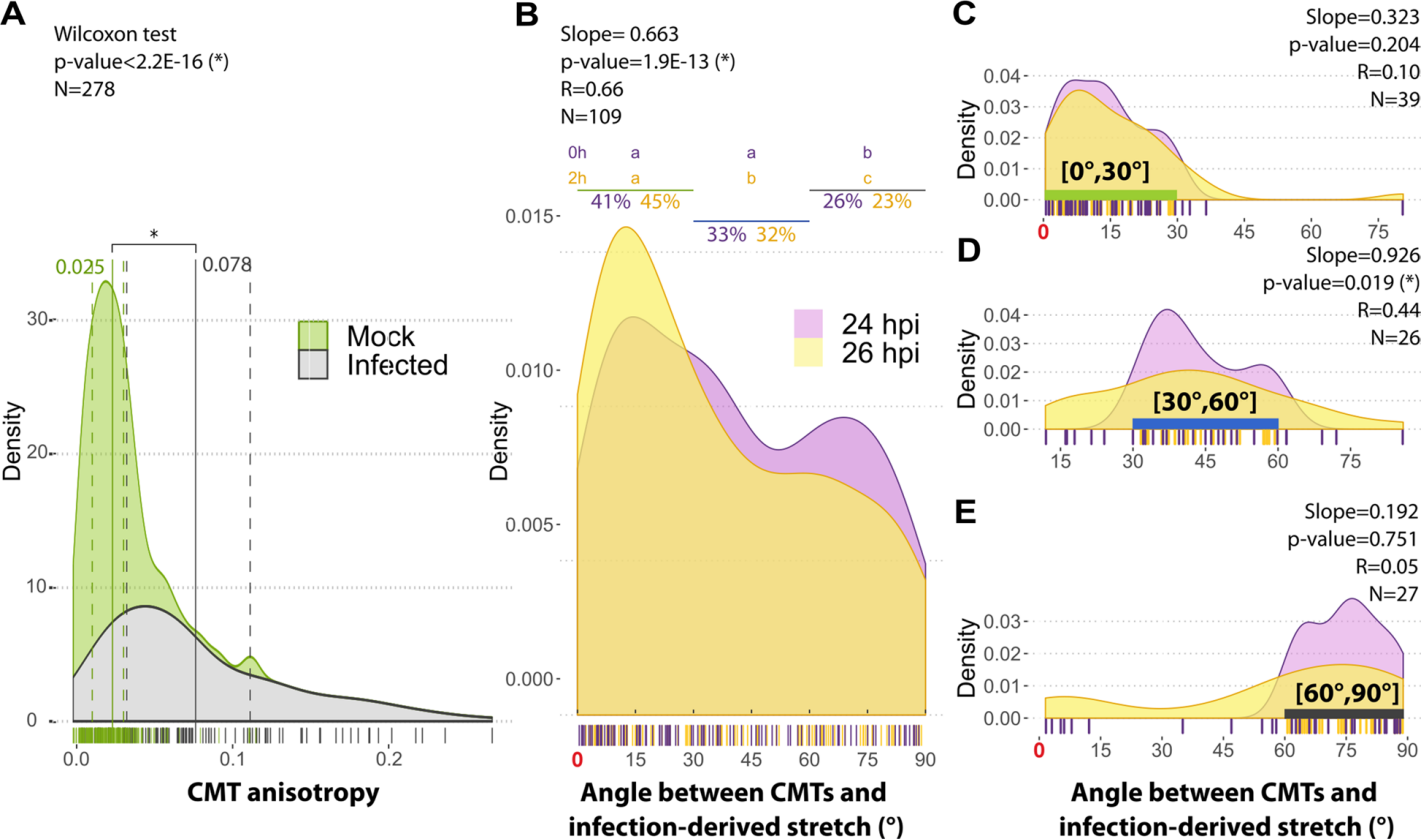
Pathogen-derived stretch guides the reorganization of cortical microtubules (CMTs) in plant cells. **A** Distributions of CMTs anisotropy *S. sclerotiorum*-infected (*n*=109 cells) and mock-treated *A. thaliana* leaves (*n*=169 cells). Solid vertical bars indicate distribution means. Dashed vertical bars indicate first and third quartiles. **B** Distribution of CMTs angle relative to the pathogen-derived stretch direction at 24 hpi and 26 hpi. The proportion of cells with CMTs oriented within 0–30°, 30–60°, and 60–90° of the pathogen-derived stretch is indicated above the graph and compared at 24 and 26hpi using a proportion test. Distribution of CMTs angle relative to the pathogen-derived stretch at 24 and 26hpi for cells with CMTs within 0–30° (**C**), 30–60° (**D**), and 60–90° (**E**) of the pathogen-derived stretch. The slope of the linear model indicated the temporal trends of angle evolution over 2h

Laser ablation experiments showed that mechanoperception occurs when the stress release exceeds shape-derived stress [15]⁠. In such a case, mechanoperception is associated with the anisotropic reorganization of the cortical microtubules in the direction of the tension variations. Consistent with these results, we found that CMT anisotropy was higher in plant cells of *S. sclerotiorum*-infected leaves than in mock-treated leaves (Fig. 2A, Additional file 4: File S1). The signal instructing the reorientation of CMTs must therefore encode the direction of the bias, i.e., encode 3D information (described by a tensorial field). While diffusive molecules more likely encode 1D information (described by a scalar field), mechanical stress and strain, 3D by nature, are prime signal candidates instructing CMTs reorganization [13, 26]⁠.

In the absence of growth (plastic deformation), in vitro and in vivo experiments suggest that CMTs align in the direction of the stretch, i.e., the elastic elongation. Our cell-deformation analysis (Fig. 1A) indicated that *S. sclerotiorum*-derived stress was measurable from 24 hpi. To determine whether cells perceive pathogen-derived stress, we monitored the angle between pathogen-derived stretch and CMTs in *Arabidopsis* epidermal cells over 2 h (24 to 26 hpi). At 24 hpi, a majority of cells exhibited CMTs already oriented between 0 and 60° from the stretch direction (Fig. 2B). Nevertheless, we detected a significant reorganization of CMTs parallel to the stretch during the subsequent 2 h. During this period, the proportion of CMTs within 30° of the stretch direction increased from 41 to 45% (Fig. 2C). When closely aligned or orthogonal with stretch, CMTs orientation remained stable (Fig. 2D, E). Alignment of CMTs along the stretch was mostly due to the reorganization of CMTs initially oriented from 30 to 60° of the stretch (Fig. 2D). Altogether, these results suggested that plant cells perceived pathogen-derived tensile stretches as mechanical cues guiding the reorganization of CMTs.

### Anisotropic CMTs patterning occurs in healthy plant cells distant from the infection

**Fig. 3:**
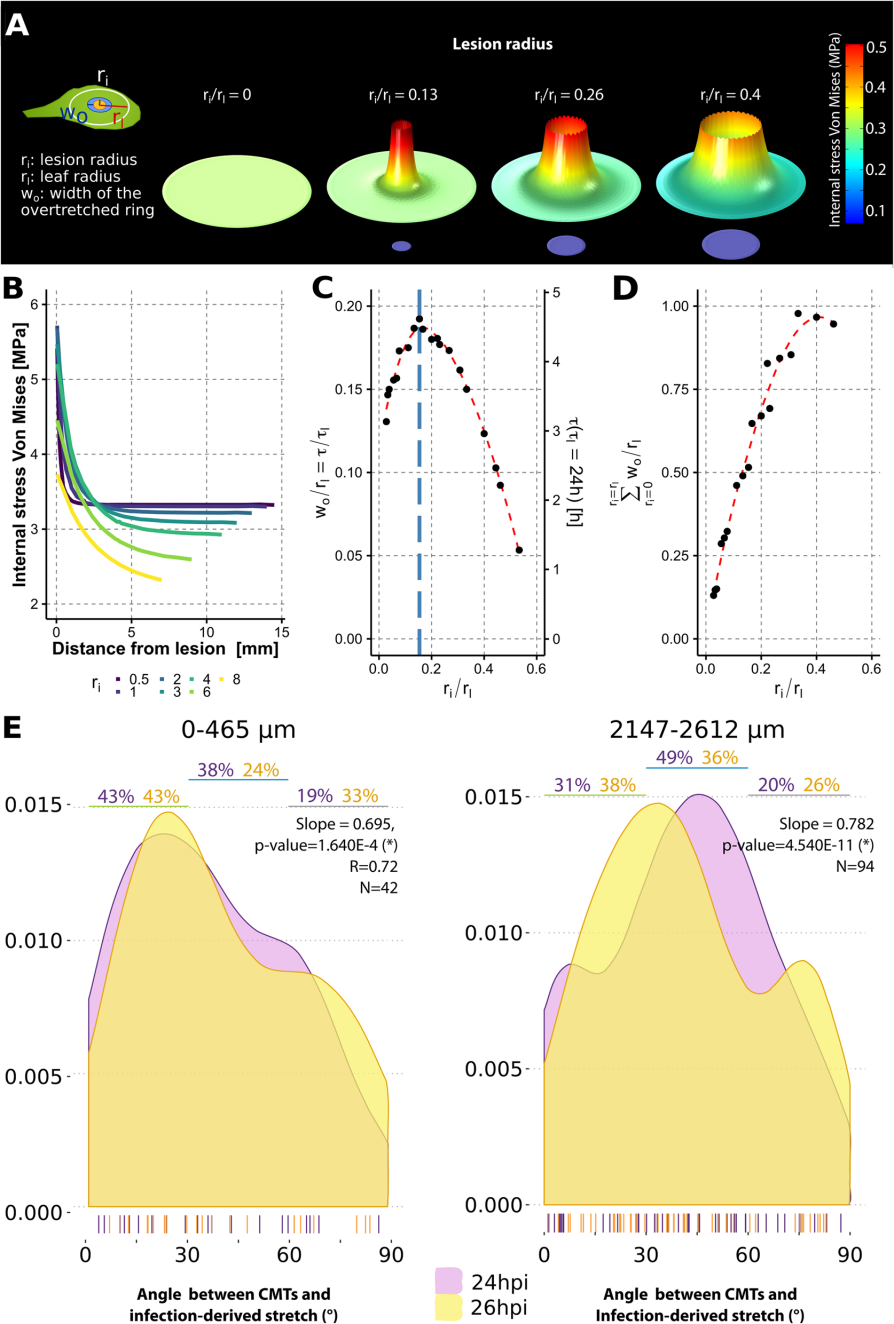
Pathogen-derived cues shape the reorganization of CMTs in a stress fluctuation ring around the colonized area. **A** Biomechanical model of infected leaf. Healthy tissue was initially under isotropic tension (left). The infection releases progressively the internal tension (from left to right) that reshuffle internal stress through tissue and creates a pathogen-derived overstretched ring around the colony. **B** Infection-derived stress (*Y*-axis) relative to distance from the lesion (*X*-axis) and the size of the virtual lesion (colors). The shape of the stress-distance profiles was only dependent on the size of the lesion. For lesions below 6 mm of radius, the width of the overstretched ring was larger than 2mm. **C** Leaf overstretched proportion (w_0_/r_l_, *Y*-axis) was only dependent on the relative size of the infection (r_i_/r_l_). The right *Y*-axis shows the shortest delay in hours between the pathogen-derived stress fluctuation and contact with the lesion (τ) assuming elastic tissue, constant lesion growth speed, and a representative time of 24h to infect a whole *A. thaliana leaf* (τ_l_). Stress fluctuations arise in the typical time of 1 h ahead of contact with the lesion. **D** Cumulative overstretched proportion of leaves relative to growth of the lesion. Almost 100% of cells were overstretched during the infection. **E** Patterns of pathogen-derived stretch and CMT orientation within a 0.5 mm (left) and 2.5 mm (right) range from the area colonized by *S. sclerotiorum* at 24 hpi and 26 hpi, measured from the analysis of confocal micrographs (*N*=42 and 94 cells respectively). Individual measures are shown as bars along the *x*-axis, the % of cells with CMTs oriented within 0–30°, 30–60°, and 60–90° of the pathogen-derived stretch is indicated above the graph, and slope of the linear model indicated the temporal trends of the distribution

Our observations suggested that the perception of pathogen-derived mechanical cues was not restricted to cells immediately adjacent to the colonized area. Plant tissue structure may favor the transmission of pathogen-derived mechanical cues to inform distant cells of the presence of a pathogen. The transmission of pathogen-derived mechanical cues are mostly related to two different mechanisms that could involve variations of cell wall stress. The first mechanism would rely upon enzymes and oxalic acid diffusion. The second mechanism would be caused by the release of tissue tension associated with the necrosis formation containing the center of the mycelium colony [6]⁠. In necrosis, most plant cells are destructed and are not turgid anymore. This localized loss of turgor pressure may involve stress fluctuations in the apoplast and a water flow required to reach a new mechanical equilibrium [27]⁠. Mechanical cues should propagate more rapidly than the fungal growth to be effective. The fungal growth is in the magnitude of 1 μm/s [28]⁠. A 1-mm growth typical time would be τ_G_ ~ 10^3^ s ~ 1 h. Enzyme or acid would diffuse from the colony periphery to the host tissue. The diffusion time increases quadratically with the distance. Although depending on the cellular context, diffusion coefficients are in the magnitude of 10 μm^2^/s for enzymes and 10^3^ μm^2^/s for H_3_0^+^ [29]. The shortest typical diffusion time required for a 1-mm diffusion in host tissue would be τ_D_ ~ 10^3^ s and is similar to the fungal growth typical time. The typical time of the turgor pressure reshuffling triggered by the localized necrosis-associated turgor loss is given by the poroelastic time. As diffusion, poroelastic time scales with the distance square. The typical value of the water diffusion coefficient is 10^2^ μm^2^/s [30, 31]⁠. Previous results suggested that 1% strain triggered CMTs reorganization. The typical time required to strain by 1% cells at 1 mm of the lesion would be τ_P_ ~ 1 s. The turgor pressure reshuffling caused by the turgor loss associated with the lesion may be effective infection-derived mechanical cues perceived by cells distant from the infection site. These biomechanical considerations suggested that this mechanism would act in parallel with the deterioration of cell wall mechanics of healthy cells caused by acid and enzyme diffusion. To estimate the distance to which the lesion may reshuffle internal stress during infection, we designed a 2D mechanical elastic model at the scale of the leaf to compute the spatial stress release caused by infection (Fig. 3A). The progress of the lesion over time was modeled by the radial growth of a virtual pathogen colony (r_i_) from 0.5 to 10 mm in modeled leaves. Since lesions are composed of destructed cells, we assumed no internal stress and low elastic modulus in the infected area. Leaves were modeled as circles with different radii (r_l_), Young’s modulus, and internal tension density representative of *A. thaliana* leaves (Additional file 5: Table S1). Simulations showed that the pathogen-derived stress developed an overstretch ring adjacent to the infection site in which tension was higher than the initial tension (Fig. 3A, B). The relative width of the overstretched ring (w_o_/r_l_) depended only on the lesion radius (Fig. 3C). The relative width of the overstretched ring increased with colony growth (r_i_/r_l_) until the colony reached ca 15% of the leaf width. At this threshold value, almost 20% of the modeled leaf radius was overstretched. As the colony kept growing, the proportion of the overstretched ring decreased and vanished when colony exceeded ca 50% of the leaf radius showing that the overstretch ring depended on the number of destructed and non-turgid cells but also on the number of turgid cells remaining in the tissue. During the course of the colony growth, almost 100% of the leaf radius got overstretched at some point (Fig. 3D). The transmission of stress fluctuations in the overstretched ring preceded the presence of the pathogen. Under the elastic tissue assumption, these results suggested that the lesion resulting from fungal infection may cause stress fluctuations in almost all living cells of the leaves before they eventually get infected. The transmission of stress fluctuations in the overstretched ring preceded the presence of the pathogen. Kinematics of leaf infections suggests that the growth speed is nearly constant once the infection started [28]⁠. Under this assumption, the delay between pathogen-induced stress fluctuation and contact with invasive hyphae was estimated as τ/τ_l_ = w_o_/r_l_, with τ_l_ the time required to infect a r_l_ radius leaf (Fig. 3C). The delay between stress fluctuation and the presence of the pathogen depends on the relative size of the colony and was always over 10% of the time required to infect the whole leaf. Infection experiments show that the representative time τ_l_ of *A. thaliana* leaf by *S. sclerotiorum* is about 24h [28]⁠. Therefore, the typical time between stress fluctuations and contact with invasive hyphae is about 1 h. These results established for pure elastic tissue suggested that the turgor loss in the lesion site triggered the reshuffling of cell wall stress around the lesion site. In viscous tissue, the stress fluctuation transmission in cell walls is damped by mechanical energy dissipation. It involves than in plant tissue, the overstretched ring’s effective thickness and the time between the signal perception and the contact with the colony would be smaller. Previous experiments have shown that cell wall stress fluctuations induced by large tissue laceration are transmitted at a larger scale [15]⁠ and trigger CMTs reorganization. Cut and laser ablation experiments demonstrate that the signal transmission distance depends on the amount of internal stress released by the cut. At 24 hpi, a significant amount of internal stresses was already released by about the 5-mm radius colony (Fig. 1A). The mechanical stress fluctuations caused by the release of internal tension, enzymes, and acid diffusion may be infection-derived mechanical cues perceived by cells in a stress fluctuation ring.

In agreement with this biomechanical analysis, we observed the reorganization of CMTs over 2 h (24–26 hpi) in the stress fluctuation ring around distal hyphae of *S. sclerotiorum* in *Arabidopsis* leaf epidermal cells expressing the MAP4:GFP construct. At 24 hpi, 43% of the CMTs within ca. 0.5 mm from the fungal hyphae were within 30° of the direction of the infection-derived stretch and remained stable over 2 h (Fig. 3E). At c.a. 2.5mm from the infection site, the proportion of CMTs within 30° of the direction of pathogen-derived stretch increased from 31 to 38% (Fig. 3E). The global organization of CMTs along the direction of the stretch appeared dependent on the distance from the infection site. Close to the infection site, the alignment of CMTs with mechanical cues was already clear at 24hpi, whereas the reorganization was initiated but incomplete in distant cells. We conclude that mechanical stretch caused by pathogen infection was perceived at a supra-cellular level and triggered the CMTs re-orientation.

### Mechanoperception of pathogen-derived cues mediated by CMT anisotropic patterning was associated with the expression of disease resistance genes in distal cells and impacted the disease resistance phenotype

**Fig. 4:**
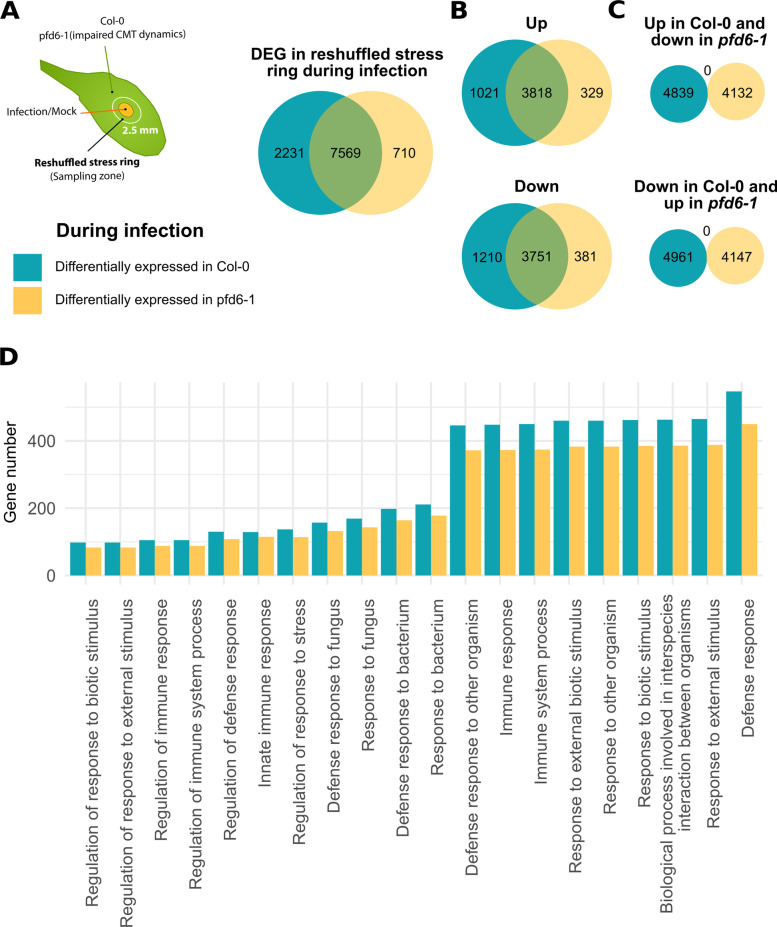
Effect of impaired CMTs dynamics on the expression of genes associated with plant disease resistance in the stress fluctuation ring. **A** Most of genes differentially modulated during infection in the stress fluctuation ring were expressed independently on genotype. Few genes are specifically modulated in *pfd6-1* with decreased CMT dynamics (710 genes) independently of the up- or downregulation (**B**). All genes are modulated in the same direction in both genotypes (**C**). **D** Number of genes differentially expressed in the 20 most enriched ontologies

We reasoned that, if adaptive, the CMT reorganization in the direction of pathogen-derived mechanical cues in the stress fluctuation ring is likely to influence plant immune responses. To test this hypothesis, we took advantage of the *A. thaliana pfd6-1* mutant in Col-0 background that exhibits a nonsynonymous nucleotide substitution in the prefoldin 6 gene (At1g29990) with no effect on the expression level (Additional file 6: Fig S4) and leading to decreased CMT dynamics [32]⁠. First, we measured the expression of genes in the *pfd6-1* and Col-0 wild-type genotypes for healthy and infected plants (Fig. 4A, Additional file 7: File S2). Samples were extracted from the 2.5-mm stress fluctuation ring around the inoculated or mock-treated area, in which no disease symptoms were visible. In agreement with the limited genetic difference between Col-0 and *pfd6-1*, the differential gene (DE) analysis [33] ⁠of healthy plants indicated that both genotypes have similar basal transcriptomic activity (Additional file 8: Fig S5). Only 51 over 32,827 genes (0.1%) were differentially expressed basally and not considered for further analysis (Additional file 9: Fig S6). Infection triggered the differential expression of around 25 % of plant genomes (Fig. 4A). A reduced number of genes was modulated in *pfd6-1* (8279 genes) compared to Col-0 (9800 genes) (Fig. 4A). The DE genes around the necrosis appeared partly dependent on the CMT dynamics (Fig. 4A). 7569 genes were modulated in both genomes, whereas 2231 (*resp.* 710) were modulated specifically in Col-0 (*resp. pfd6-1*). Interestingly, the sign of gene expression variations was not affected in the *pfd6-1* mutant line (Fig. 4B, C), suggesting that impaired CMT dynamics could cause targeted gene expression modulation rather than bulk deregulation. More than 50% of the 1671 genes modulated by rain and touch treatments [34]⁠ were also modulated during infection around the lesion site (50.4% in *pfd6-1*, 58.0% in Col-0). To further evaluate whether the reduced number of genes expressed in *pfd6-1* affected plant function, we performed a gene enrichment analysis [35] ⁠and selected the 20 pathways with the lowest enrichment false discovery rate (Fig. 4D, Additional file 10: File S3). All pathways identified were connected with plant defense response. For every pathway, such as defense response to fungus, the modulated gene number was smaller in the *pfd6-1* mutant line (Fig. 4D), suggesting that some resistance-associated genes expression correlated with CMTs reorganization dynamics in the stress fluctuation ring.

**Fig. 5:**
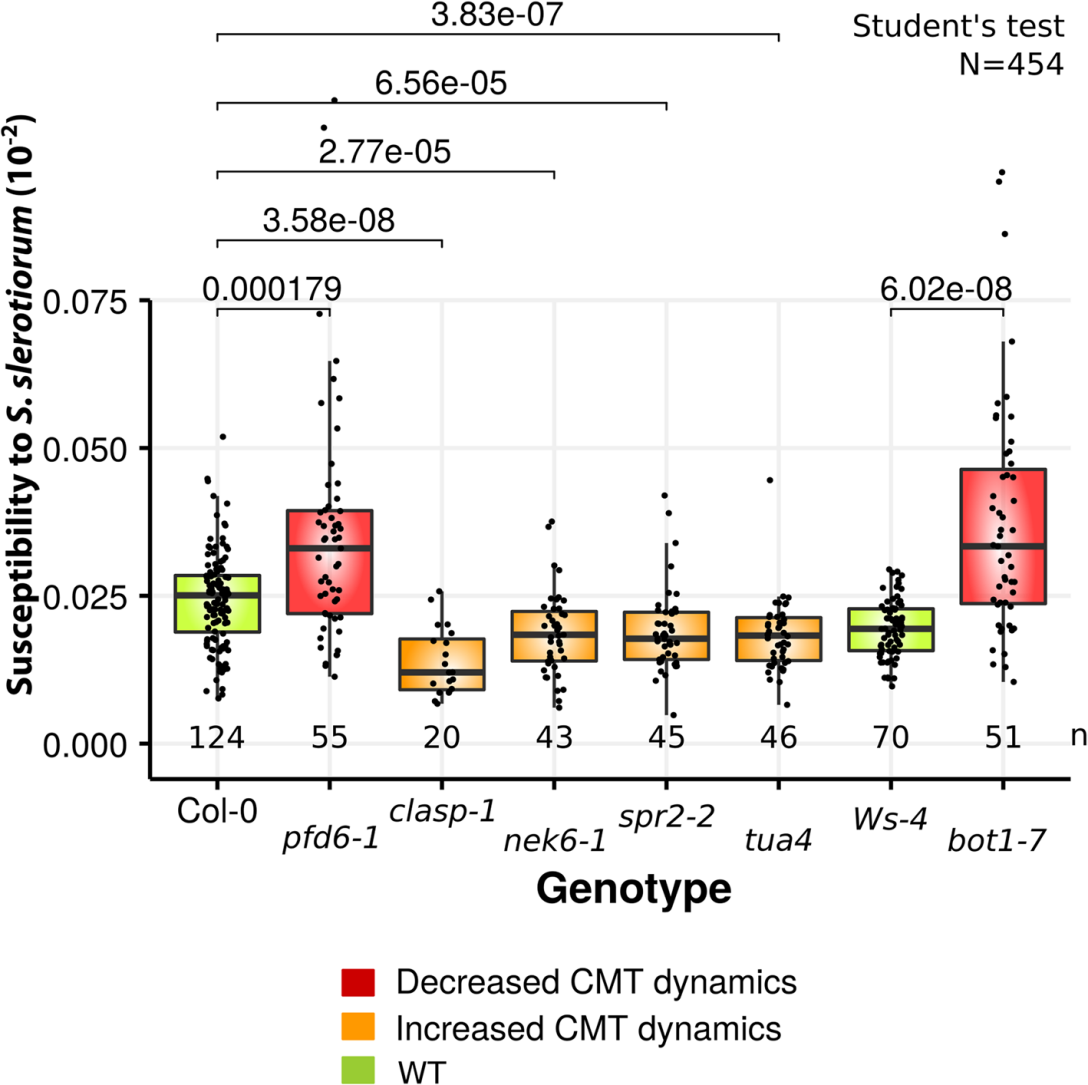
Effect of CMT dynamics on plant susceptibility to *S. sclerotiorum*. Plant susceptibility to *S. sclerotiorum* fungal pathogen was drastically increased in mutants that exhibited decreased CMT dynamics (*pfd6-1, bot1-7* in red) compared with wild type (Col-0, Ws-4 in green). Conversely, mutants with increased CMT dynamics exhibited a decreased susceptibility to the fungal pathogen (*clasp-1*, *nek1-6*, *spr2-2*, *tua-4* in orange). Values were computed on 454 leaves analyzed in three independent assays. Means were compared by *t*-test and significant *p*-values are indicated over brackets. Number of leaves per genotype is indicated under every boxplot. Boxplots show first and third quartiles (box), median (thick line), and the most dispersed values within 1.5 times the interquartile range (whiskers). ns. non-significant

Finally, to evaluate how pathogen-induced CMT reorganization contributed to the overall plant resistance, we compared susceptibililty to *S. sclerotiorum* in mutant lines affected in CMTs dynamics such as *pfd6-1*, using image-based time-resolved phenotyping [28]⁠. *bot1-7* is a katanin mutant allele in the Ws-4 background affected in the CMTs severing process that exhibits a reduced response to mechanical stress [15]⁠. Conversely, nek6-1 impaired in CMTs depolymerization [36]⁠, *spr2-2* [37]⁠, *clasp-1* [38],⁠ and *tua4* [39]⁠ exhibit enhanced response to mechanical stress. The plant susceptiblity to fungal pathogen was associated directly with the ability to form CMTs anisotropic patterning (Fig. 5, Additional file 11: File S4). Mutant lines exhibiting enhanced response to mechanical stress were less susceptible to *S. sclerotiorum* than wild-type. *clasp-1*, exhibiting hyper-response toward mechanical cues was the most resistant mutant line. Inversely, the fungal pathogen colonized *pfd6-1* and *bot1-7* significantly faster than the respective wild type, indicating that CMT dynamics contributed at least to ca. 40% of the disease resistance phenotype (Fig. 5). Altogether our results show that the mechanoperception of pathogen-derived cues in leaves reshuffle internal stress around the infection site, triggering CMT anisotropic patterning and controlling the expression of disease resistance genes in distal cells that contribute to the overall resistance phenotype.

## Discussion

The plant immune response is a spatio-temporal process orchestrated by multiple signals. Known signaling cascades are initiated by the interaction of pathogen-associated molecular patterns with cellular receptors. However, signals governing plant systemic immunity remain incompletely understood [40, 41]⁠. By combining modeling, image analysis, biomechanics, and molecular biology, we show that mechanical cues potentiate plant resistance during infection by necrotrophic fungi. We found that mechanical stress release in cell walls, likely due to hydrolysis by pathogen enzymes triggered mechanical cues guiding the reorganization of CMTs. We also found that pathogen-derived stress fluctuations inform living epidermal cells around the infection site during infection. In plant cells distant from the fungal colony, CMT reorganization instructed by pathogen-derived mechanical cues regulates the expression of some resistance-associated genes. Finally, we show that the transduction of pathogen-derived mechanical cues associated with CMT anisotropic patterning contributes to at least 40% of the disease resistance phenotype. This drives us to propose that mechanoperception plays an active role in the spatio-temporal implementation of plant resistance to fungal pathogens. Such a mechanical triggered immunity (MTI) would act in complement with other signals.

In this work, we focused on CMT-associated mechanoperception. The *pfd6-1* and *bot1-7* mutant lines are impaired in CMT dynamics [32, 42]⁠. Therefore, alterations in their disease susceptibility and resistance gene expression patterns result primarily from the ability of CMTs to reorient in the direction of the principal stretch (Fig. 2). It suggests that the mechanoperception of pathogen-derived mechanical cues through CMT anisotropic patterning is a prominent mechanism for plant immunity regulation. Mechanoperception is also involved in cell wall integrity (CWI) maintenance mechanisms that regulate cell wall composition dynamically during growth. CWI maintenance mechanism may also rescue the loss of pattern-triggered immunity (PTI) signaling [20]⁠. Responses to cell wall damages are achieved partly by hypo-osmotic perception channels MCA1, MSL2, and MSL3 activated by the increase of tension in cell walls [20]⁠ and by proteins such as CrRlks THE1 and FER wall sensors [43]⁠. Cell size deformations observed around the fungal colony (Fig. 1B, C) are associated with turgor pressure fluctuations. Consequently, CWI-proteins are most likely also involved in the stress fluctuation perception. It would suggest that MTI would rely on different mechanoreceptive proteins in addition to CMTs.

How CMT and osmo-sensitive channel mechanoperception pathways regulate plant immunity remains elusive. Our disease susceptibility analysis of *pfd6-1* and *bot1-7* suggests that CMT mechanoperception acts either in parallel or upstream of other mechanoperception pathways. Interestingly, in CWI maintenance CMTs acts in parallel to the pleiotropic CrRLK FER involved in many plant responses including innate immunity and growth [43]⁠. Some mechanosensitive channels such as MCA1, OSCA, and PIEZO contribute to calcium signaling. However, at least in the shoot apical meristem, the CMT reorganization in the direction of the principal stress is independent of calcium [44]⁠. Conversely, CMT anisotropic patterning participates in cell-to-cell transmission of mechanical tension that favors the opening of mechanosensitive channels. Calcium signaling is closely linked with ROS (reactive oxygen species) production [7]⁠ that participates in the CMT dynamic organization regulation [45]. These mechanisms may form a self-organizing regulation loop. The CMT anisotopic patterning triggered by pathogen-derived mechanical cues could initiate this loop by promoting calcium influx through the mechanosensitive channels opening that triggers ROS production and *in fine* promotes CMT anisotropic patterning.

We found that the deformation of plant tissue by the fungal pathogen, likely through enzymatic degradation of an important number of cells and enzyme and acid diffusion, produced a stress fluctuation ring around the fungal colony (Fig. 3). Pathogen-derived mechanical cues are expected to be transmitted in a non-cell autonomous manner, because it is transmitted by fluctuations cell wall mechanical properties (Fig. 4). Plant immunity involves quasi-instantaneous signals such as ROS and calcium waves that propagate at ~1mm/s in *A. thaliana* leaves [46]. The propagation of ROS and calcium signals requires an active relay from one cell to the next that can be manipulated by pathogen effectors [47, 48]⁠. For instance, *S. sclerotiorum* secretes the SsITL protein reported to inhibit host immunity by interacting with the sensing calcium receptor CAS [48]⁠. CMTs are also targeted by pathogen effectors such as the *Pseudomonas syringae* effector HopZ1a [49]⁠. Although the perception of infection-derived mechanical cues by CMTs is cell autonomous, their propagation through the cell wall is non-cell autonomous. Therefore, the perception of mechanical signals may be altered by pathogen effectors with no impact on their transmission. Furthermore, the disruption of both molecular and mechanical signal perception by pathogens relies on the diffusion of effectors. The diffusion time increases with the square of the distance implying that signal manipulation by pathogens is mostly efficient in host cells close to the pathogen. As a result, cell autonomous molecular signals would be more sensitive to pathogen hijacking than mechanical cues transmitted passively by the tissue structure. The specific properties of mechanical signals should therefore ensure the robustness of mechanosignaling in an infection context.

Our results echo the gain of resistance conferred by mechanical stimuli observed previously [50–52]⁠. Soft repeated mechanical stimuli applied by rolling or brushing leaves conferred gain of resistance to the necrotrophic fungus *Botrytis cinerea*. Despite being not mandatory to obtain a gain of resistance, trichomes are involved in the mechanoperception of raindrops which activate immunity [53]⁠. The gain of resistance obtained by the repetition of mechanical stimuli could be used to develop methods for sustainable cultural practice like the traditional Japanese mugifumi [54, 55]⁠. Altogether these results provide a scenario in which the regulation of immunity by pathogen-derived mechanical cues is sensitized by the perception of repeated abiotic stimuli. Remarkably, in contrast to what has been found in poplar, there is currently no report of *Arabidopsis* acclimation to abiotic mechanical signals that may result in the increase of plant susceptibility to infection. The challenge of quantifying the amount of mechanical signal that scales with the sum of strain [56, 57] is undoubtedly one of the locks to overcome to further understand how mechanoperception of abiotic mechanical signals regulates immunity. The MTI modulation by repeated abiotic mechanical stimuli would form thigmoimmunity by analogy with thigmomorphogenesis describing the shape regulation by mechanical signals [58]⁠.

Mechanoperception is found to be central to many plant functions that are essential to its survival. How tensorial variations in stress and deformation of the cell wall matrix can regulate multiple functions remains a key question. A simple explanation with complex consequences would be that the specificity of the response is obtained by a combination of signals of different nature. The resulting interlaced signaling system initiating the implementation of specific immunity would be poorly robust and readily diverted by pathogens, but may be backed-up by a more general stress response triggered by mechanical signals alone.

## Conclusions

We describe a role for CMTs in connecting mechanoperception and defense regulation in plants, a process we propose to refer to as mechano-signaling triggered immunity (MTI). The solid-like mechanical structure of plant tissue composed of bound cells surrounded by stiff walls favors the transmission of pathogen-derived mechanical cues through the modification of mechanical properties of the apoplast. Mechanoperception occurs in healthy cells around the infection site and does not require the loss of wall integrity but only the tensorial variation of stress [59]. Our results highlight the mechanoperception of pathogen-derived stress as a distinct layer of the plant defense system with unique properties. Well-established mechanisms of plant immunity include pathogen-associated molecular pattern (PAMP)-triggered immunity (PTI) which involves the perception of pathogen-derived molecules of damage-associated molecular patterns (DAMPs) derived from plant molecules [60]⁠. The MTI layer of plant immunity differs from previously described mechanisms by the non-molecular nature of the signals that activate it. Infection-derived mechanical cues are spatialized since local cell wall hydrolysis involves a supra-cellular stress reshuffling in the vicinity of pathogen-colonized cells. Infection-derived mechanical cues contain oriented 3D information (tensorial). Because of the intrinsic properties of mechanical cues, MTI could orchestrate spatially, around the infection site, a non-specific form of resistance and boost molecular immune responses. The spatial propagation of pathogen-derived mechanical cues would explain partly the spatial heterogeneity of gene expression observed during infection [41]⁠. Recent results suggest that perception of cell damage caused by infection is not mediated by DAMPs only and involves additional cues [5]⁠. We propose that pathogen-derived mechanical cues, generated across plant cells by the release of internal tension, acts as additional non-molecular cues triggering defense response to fungal infection. In contrast with molecular-based defense layers, mechanical cues cannot inform cells of the nature of the attacking pathogen. The so-called mechano-signaling triggered immunity (MTI) would complement thus the signals involved in PTI and effector-triggered immunity (ETI).

## Methods

### Plant/fungus materials, culture conditions, and inoculation

*Arabidopsis thaliana* plants were grown at 23°C with 9 h/day of light for 5 weeks. *Sclerotinia sclerotiorum* was grown on PDA (Potato Dextrose Agar, Fluka) plates at 23°C for 4 days in the dark prior to inoculation. Inoculations were performed by depositing a 5-mm diameter plug of culture medium containing mycelium on the upper surface of leaves. Mock treatment consisted of a 5-mm diameter plug of mycelium-free PDA. For quantitative RT-PCR analyses, inoculated plants were placed in mini-greenhouses during 24 h to keep a high humidity level promoting infections. For phenotypic analysis, 3 leaves per plant were cut and put in Navautron devices to monitor the kinematic of lesion development [28]⁠. Plant susceptibility to infection was estimated from lesion growth kinematics. For each leaf, the susceptibility corresponded to the growth rate of the lesion during the exponential phase. The susceptibility μ was obtained by fitting the curve described by S(t) = A.exp(μ .t) with S the lesion size, t the time. The more the growth rate, the more the plant susceptibility to infection. Genotype susceptibilities to *S. sclerotiorum* infection were compared by *t*-test (Fig. 4B). Susceptibility to *S. sclerotiorum* is available in Additional file 11: File S4.

### Quantitative RNA analysis

Plant samples used for the measurement of gene expression were collected 24 h post-inoculation (hpi). The leaves were cut at the base of the petiole and immediately placed on a glass plate cooled by liquid nitrogen. For each leaf, 2.5-mm wide leaf rings were cut around the necrosis and stored at −80°C. RNA extraction was performed using the Nucleospin RNA kit (Macherey-Nagel). cDNA was synthesized with Superscript III reverse transcriptase (Invitrogen Carlsbad, CA) using 1 μg of total RNA. RNAseq reads were mapped simultaneously against *A. thaliana* Col-0 (TAIR10 release) and *S. sclerotiorum 1980* genomes, using nf-core/rnaseq pipeline version 3.0 [61]⁠ with the following parameters “--skip_alignment --pseudo_aligner salmon” that performs adapter and quality trimming with Trim Galore software version 0.6.6. then transcript assignation and quantification with salmon tool (version 1.4.0). RNAseq data underlying this article are available in Sequence Read Archive (SRA) at https://identifiers.org/insdc.sra:SRP400889 with SRP400889 the project number (Additional file 12: Table S2). The differential gene analysis of *A. thaliana genes* was performed using iDEP.96 [33]⁠ online methods. Pre-process step consisted of the normalization of counts by the edgeR log2(CPM+c) methods. The minimal count per million (CPM) was set at 0.5 and the pseudo count c set at 4. RNA sequencing data consistency was successfully checked by a PCA analysis (Additional file 8: Fig S5). Differentially expressed genes were identified by DESeq2 method with a false discovery rate (FDR) set to 1E−3 and a min fold change set to 1 (Additional file 7: File S2). Fifty-one genes with differential basal expression were identified by computed DEG in mock-infected genotype. For the computation of DEG during infection, we considered two modalities (Infected, Mock) and two genomes (Col-0, *pfd6-1*). The gene enrichment analysis was performed using ShinyGO [35]⁠ on all DEGs not basally differentially regulated (Additional file 10: File S3). Genes differentially expressed after raindrops and touch treatments were extracted from [34]. The 1671 genes modulated independently on the mechanical stimuli were considered for the analysis.

### Confocal imaging

MAP4-GFP Arabidopsis [62]⁠ leaves were imaged between 24 and 26 h post-inoculation (hpi) with infections by *S. sclerotiorum* expressing OAH1::GFP, using a Leica TCS TCSPC SP8 confocal microscope with a HC FLUOTAR 25×/0.95 water objective. These timepoints were chosen so that successful infection events could be identified visually for further analyses. Preparations were sealed with Vaseline to avoid evaporation and to limit spurious leaf displacements. Images were acquired at 1024×1024 pixels with 4× line average and a z-stack step of 0.8μm. GFP fluorescence was excited with the 488-nm ray line of an argon laser and recorded in the 505 to 530 nm emission range.

### Image analyses

Noise and isolated pixels in image stacks were removed by applying a median filter (Fiji > radius=3). The z-projection of the maximum pixel intensity was used as the input image for the computation of the CMT anisotropy, CMT angle, and the displacement field (Fiji > Z Stack). A binary mask was drawn manually using a graphical tablet to separate the plasma membranes and cell walls from the cortical microtubules (CMTs). For every completely visible cell at 24 and 26 hpi, the mean CMTs angle (in 0°, 180°) and anisotropy (0 = purely isotropic, 1= purely isotropic) were computed on masked images using fibriltool Fiji plugin [63]⁠. We used the ST+KLT method [64]⁠ of the open-source Kineplant’s toolbox (https://github.com/A02l01/CRtoolbox) to compute the spatial field of displacement [24]. The maximal number of good features to track was set to 2000 and the size of the Gaussian interpolation window was set at 16 px. Cell walls and membrane displacements were obtained by convoluting the dense field of displacement given by the ST+KLT method and the inverted mask used for CMTs analysis (Additional file 13: Fig S7). All raw images used in this work were available in Additional file 14: File S5.

### Computation of inoculation-derived stretch directions and comparison with CMTs orientation

The displacement field of cell walls and plasma membranes obtained by the ST+KLT method was used for the computation of stretch directions. For every cell, a 2D linear transformation Φ was fitted to describe the spatial displacement of the cell wall over 2 h. The transformation tensor **F** was computed as the gradient of Φ. Φ was defined as **u** = **A.X** + **B** with **u** the displacement field computed by ST+KLT method, **A** a 2×2 matrix, and **B** a vector. The Green-Lagrange strain tensor **E** was computed from **F** as **E** = (**F**^T^**F−I)/2**. The two principal components of the strain tensor **E** were deduced from its eigenvectors with eigenvalues λ_1_, λ_2_. Infection-derived stretch directions were compared to the mean direction of CMTs. For λ_1_ , λ_2_ with opposite signs, the stretch direction was the direction of the eigenvector associated with the maximal positive eigenvalue. For negative λ_1_, λ_2_, no stretch direction was reported. For positive λ_1_, λ_2_, we assumed that CMTs reorganization occurred in both stretch directions. Consequently we computed the angle between the CMTs and the direction of eigenvectors bisector. The relative surface variation was computed as the sum of eigenvalues of **E**. Computations and stretch field rendering were performed using the NumPy python package [65]⁠ and matplotlib [66]⁠.

### Analyses of CMTs reorientation

The comparison of CMTs anisotropies in healthy and inoculated tissues was performed on 278 cells (109 in infected tissue, 169 in healthy tissue) corresponding to 6 different leaves (3 infected, 3 healthy) of 6 different *Arabidopsis* plants. Wilcoxon’s test was used to assess the mean difference in absolute values of the relative cell-surface variation for inoculated and healthy plants, and in phenotypic effect of impaired CMT dynamics on the susceptibility to *S. sclerotiorum* inoculation. The temporal evolution of CMT caused by pathogen-derived strain was tested by the linear model **φ**(t=26hpi) = a.**φ**(t=24hpi) +b+ε, with **φ** angles between the infection-derived stretch direction and the CMT direction. Slopes inferior to 1 indicated a convergence between CMTs angle and infection-derived stretches. We next tested variations in proportion within 30° angular sectors at constant time and between 24 hpi and 26 hpi by a proportion test, with results summarized by a letter in Fig. 2B. All statistical analyses were performed using R software [67]⁠ and plots drawn using the ggplot2 library [68]⁠.

### Modeling mechanical stress and strains during infection

Healthy plant tissue was modeled as a flat membrane with a radius of 15 mm and a thickness of 100 μm. The internal stress in healthy plant tissue due to the interaction of water in vacuole and cell walls was modeled as an in-plane isotropic tension set to 100 N/m. The Young’s modulus of the membrane was set to 1 MPa and Poisson’s modulus set to 0.4. The principal stress associated with the initial tension was c.a. 0.3 MPa and in the same magnitude as reported turgor pressure. Infected tissue was modeled by reducing Young’s and Poisson’s moduli and in-plane tension (Additional file 5: Table S1) in a subdomain of the membrane corresponding to the area hydrolyzed by the fungal colony. To investigate the spatial patterning of principal strain, the disease area was modeled by two 1-mm radius circles associated with lower Young’s modulus set to 0.1 MPa. To investigate the spatial patterning of the overstretched area, we performed a sensitivity analysis to test for the dependency of the overstretched area width on the leaf and lesion radii, in-plane tension, and Young’s modulus (Additional file 5: Table S1). Ranges of variation of these parameters are provided in Table S2. The overstretched width was deciphered by comparing the spatial tension field computed in infected tissue and the tension field obtained in healthy tissue (no fungus). The overstretched width was computed as the value for which the ratio between the latter stress fields equaled 1. Computation, modeling, and rendering were performed using COMSOL 5.2 software.

## Supplementary Information

**Additional file 1: Fig S1.** Evolution of chloroplasts and CMT fluorescence during infection. Chloroplast fluorescence (red) decreased with the diseases advances and was used as a proxy of cell viability.

**Additional file 2: Fig S2.** Absolute values of relative surface variations induced by infection-derived stresses. Stomata did not biased strains in their vicinity in mock or infected plants.

**Additional file 3: Fig S3.** In advanced hydrolyzed epidermal tissue, the CMTs are circumferential to the infected zone suggesting a circumferential stress patterning. Scale bar corresponds to 100 μm. Color codes for z depth.

**Additional file 4: File S1.** Data used for the analysis of CMTs reorganization. A row corresponds to a cell. Headers are: {*Cx, Cy, Aniso1, Aniso2, Angle1, Angle2, eps1, theta1, eps2, theta2, Id, Moda, MT_eps11, MT_eps22*}. Fields are separated by comma. *Cx, Cy* Coordinates of the center of the cell. *Aniso1* and *Aniso2* are cortical microtubules (CMTs) anisotropies 24 hours post inoculation (hpi) and 26 hpi. *Angle1*, *Angle2* are CMTs directions 24 hpi and 26 hpi. *eps1*, *theta1* are the value and the orientation of the first principal strain direction. *eps2*, *theta2* are the value and the orientation of the second principal strain direction. *Id* are the cell id. *Moda* is the test-set of the experiments (Mock, Infected). *MT_eps11*, *MT_eps22* are the angles between CMT and the principal stretch direction.

**Additional file 5: Table S1.** Parameters values used to model the effect of fungal-derived hydrolysis heterogeneities on the principal strain patterning and the dependency of the overstretched length on the lesion radius.

**Additional file 6: Fig S4.** Description of transcriptomic data relative to Col-0 and the mutant line *pfd6-1* in mock and infected condition. (A) Normed count distributions are similar and depended on the modality of infection. (B) The expression level of AT1G29990 (PFD6) was not infected on the non synonymous nucleotide substitution in the *pfd6-1* mutant line.

**Additional file 7: File S2.** Differential gene expression analysis in the overstretched ring aroune the necrosis in Col-0 and *pfd6-1* genotypes.

**Additional file 8: Fig S5.** PCA analysis of count per millions (cpm). The first PCA axis explaining 78.4% of the variance was relative to transcriptomic differences triggered by infection.

**Additional file 9: Fig S6.** Differential gene analysis computed with DESeq2 method.

**Additional file 10: File S3.** Enrichment analysis.

**Additional file 11: File S4.** Plant susceptibility to *S. sclerotiorum*. Headers are: *UIdNPsiSlopePMutationGenotypelog_slope* with *UId* a leaf identifier, *N* the leaf number, *Psi* the latency time, *Slope* the growth rate of the disease corresponding to the susceptibility, *P* codes for a experiment ID, *Mutation* describes the CMT dynamics, *Genotype* codes for the plant genotype and *log_slop*e corresponds to the log of Slope.

**Additional file 12: Table S2.**

**Additional file 13: Fig S7.** Image analysis pipeline for the CMTs reorganization and displacement field.

**Additional file 14: File S5.** Pictures used in this work (raw and analyzed).

## Data Availability

All data and material used in this work are available in additional files except RNAseq data available at https://identifiers.org/insdc.sra:SRP400889 [69]. SRA codes specific to every test set are available in Additional file 12: Table S2.

## Abbreviations

CMT: Cortical microtubules
crRLK: *Cantharanthus roseus* receptor-like kinases
CWI: Cell wall integrity
DAMP: Damage-associated molecular patterns
ETI: Effector-triggered immunity
FEM: Finite element model
GFP: Green fluorescent protein
hpi: Hours post inoculation
MscS: Mechano-sensitive channel of small conductance
MTI: Mechano-signaling triggered immunity
PAMP: Pathogen-associated molecular patterns
PRR: Pattern recognition receptors
PTI: PAMP-triggered immunity
QDR: Quantitative disease resistance
ROI: Region of interest
ROS: Reactive oxygen species
RLK: Receptor-like kinases
TPK: Two-pore potassium
WAK: Wall-associated kinases
