# Supplementary figures and images for: Pathogen-derived mechanical cues potentiate the spatio-temporal implementation of plant defense
Source: BMC Biol. 2022 Dec 27;20:292. doi: 10.1186/s12915-022-01495-w (PMC9795618; doi:10.1186/s12915-022-01495-w)

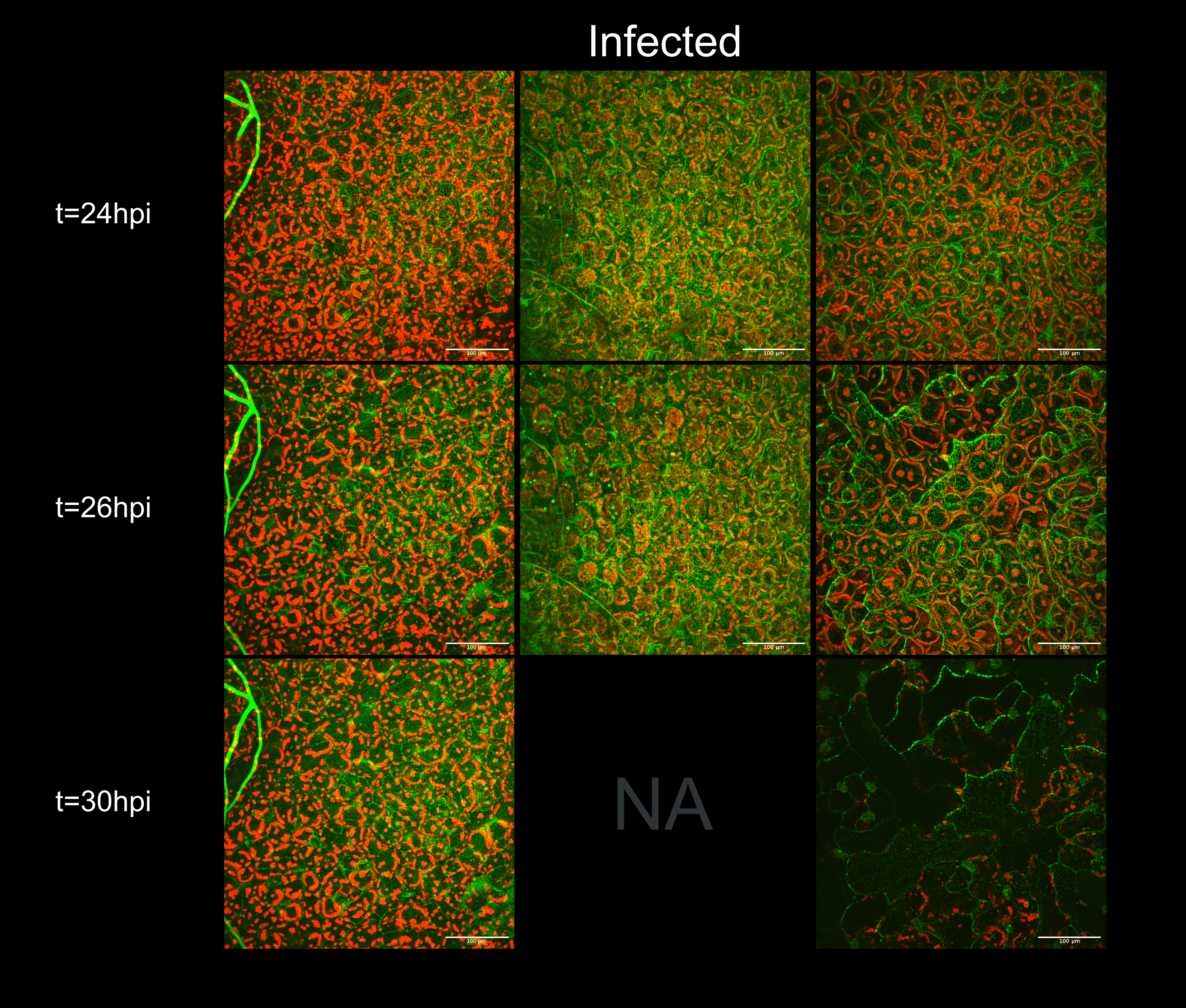

Supplement: Supplementary file 1 — Additional file 1: Fig S1. Evolution of chloroplasts and CMT fluorescence during infection. Chloroplast fluorescence (red) decreased with the diseases advances and was used as a proxy of cell viability. [file 12915_2022_1495_MOESM1_ESM.png]

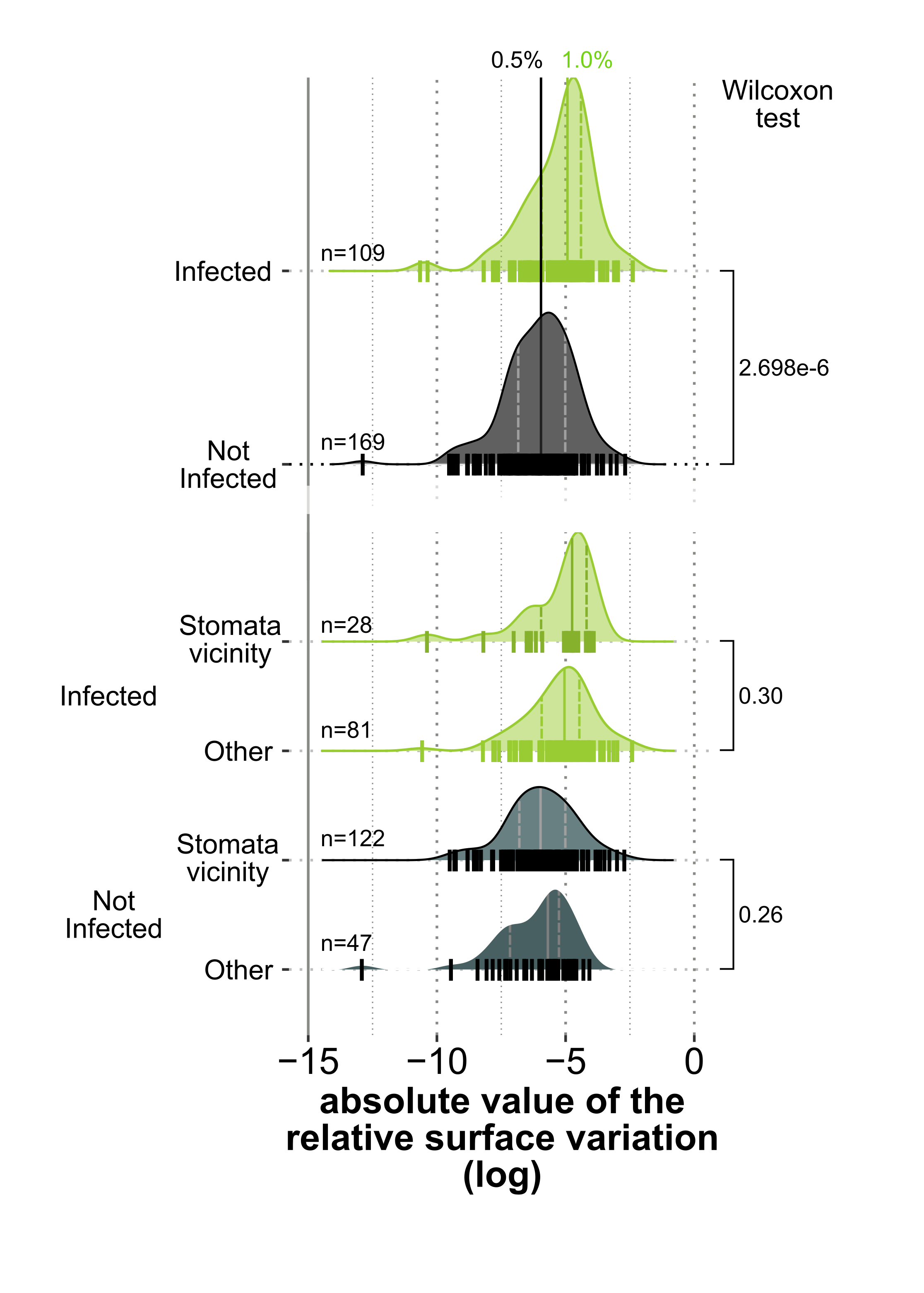

Supplement: Supplementary file 2 — Additional file 2: Fig S2. Absolute values of relative surface variations induced by infection-derived stresses. Stomata did not biased strains in their vicinity in mock or infected plants. [file 12915_2022_1495_MOESM2_ESM.png]

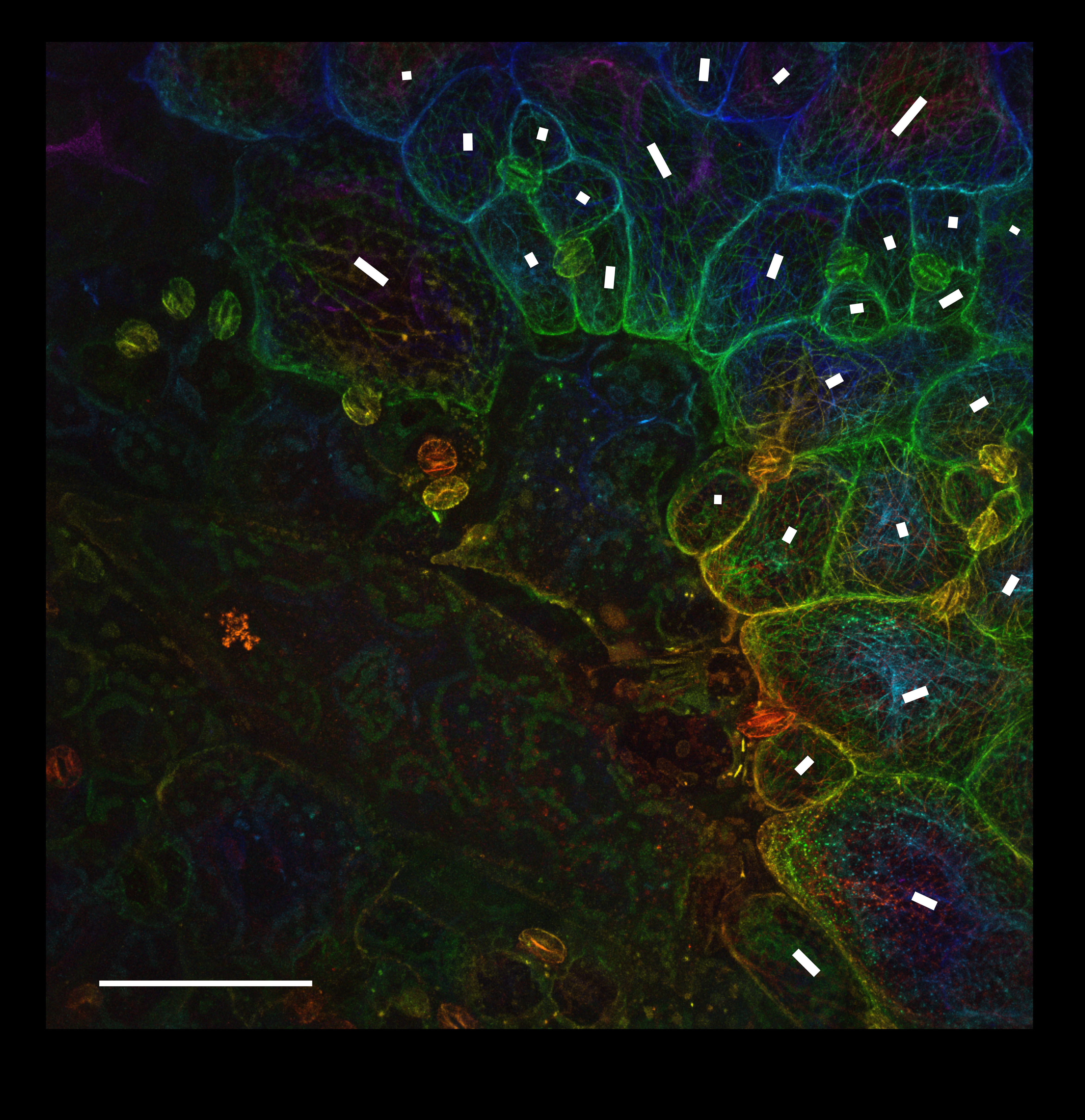

Supplement: Supplementary file 3 — Additional file 3: Fig S3. In advanced hydrolyzed epidermal tissue, the CMTs are circumferential to the infected zone suggesting a circumferential stress patterning. Scale bar corresponds to 100 μm. Color codes for z depth. [file 12915_2022_1495_MOESM3_ESM.png]

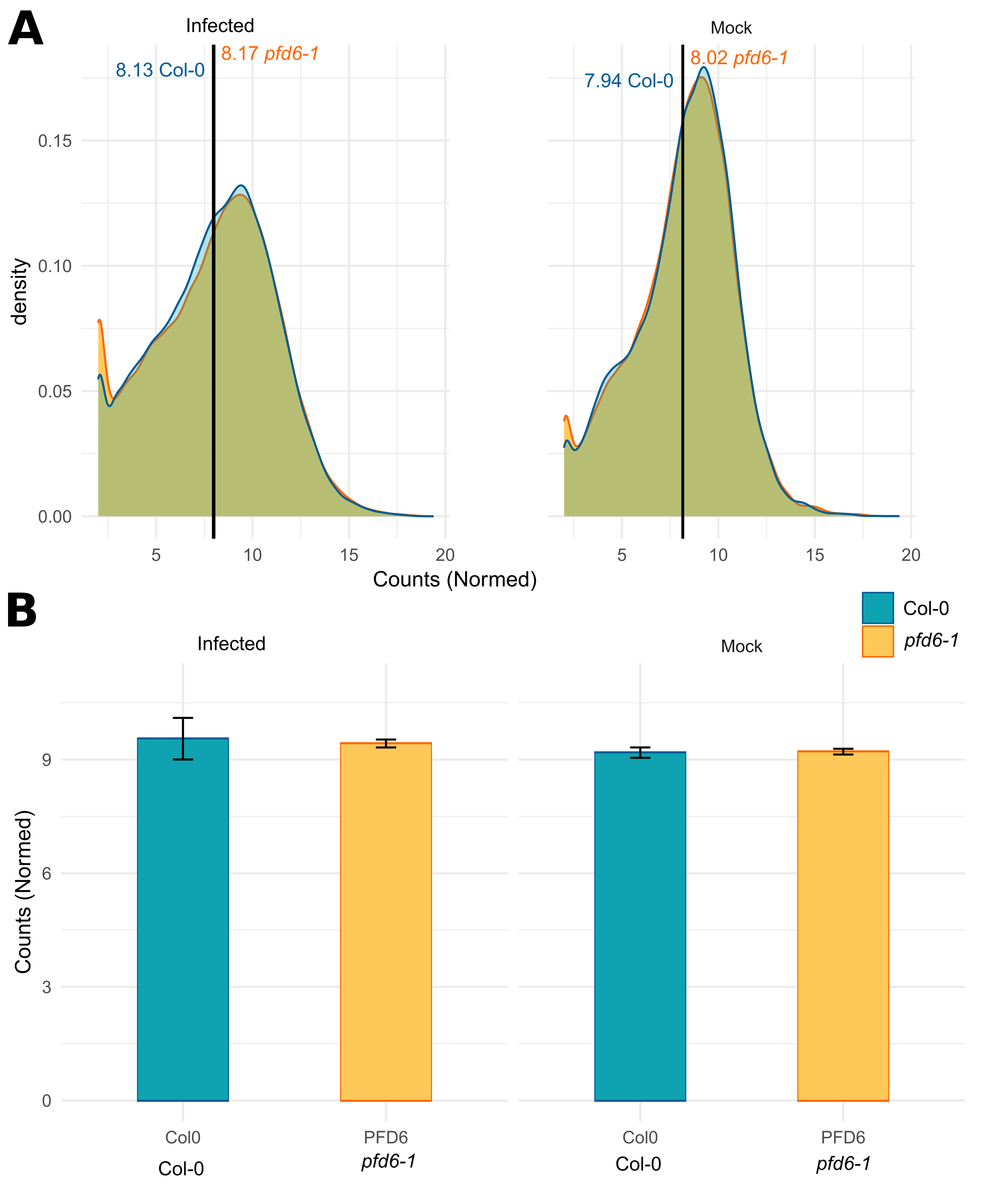

Supplement: Supplementary file 6 — Additional file 6: Fig S4. Description of transcriptomic data relative to Col-0 and the mutant line pfd6-1 in mock and infected condition. (A) Normed count distributions are similar and depended on the modality of infection. (B) The expression level of AT1G29990 (PFD6) was not infected on the non synonymous nucleotide substitution in the pfd6-1 mutant line. [file 12915_2022_1495_MOESM6_ESM.png]

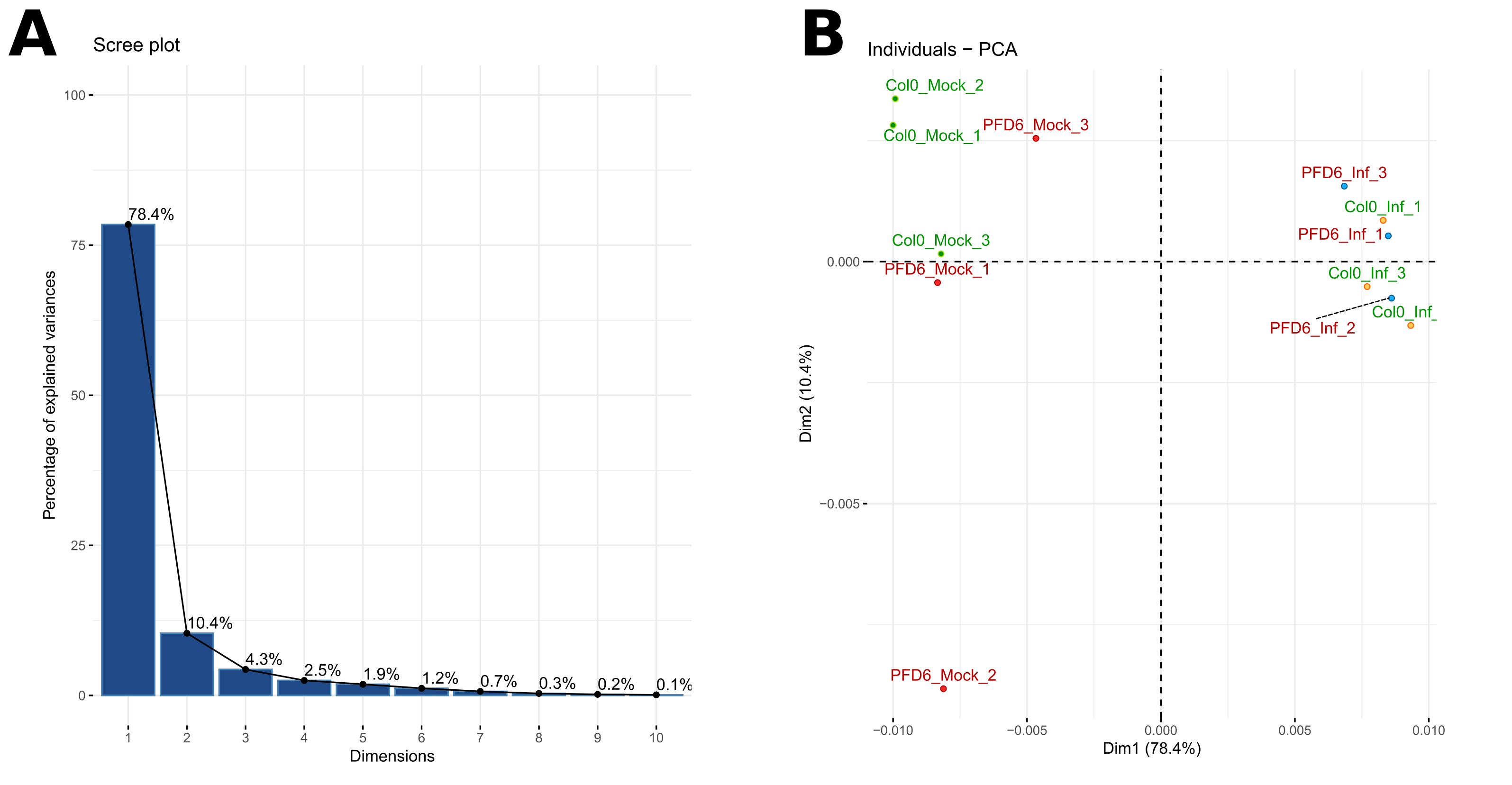

Supplement: Supplementary file 8 — Additional file 8: Fig S5. PCA analysis of count per millions (cpm). The first PCA axis explaining 78.4% of the variance was relative to transcriptomic differences triggered by infection. [file 12915_2022_1495_MOESM8_ESM.png]

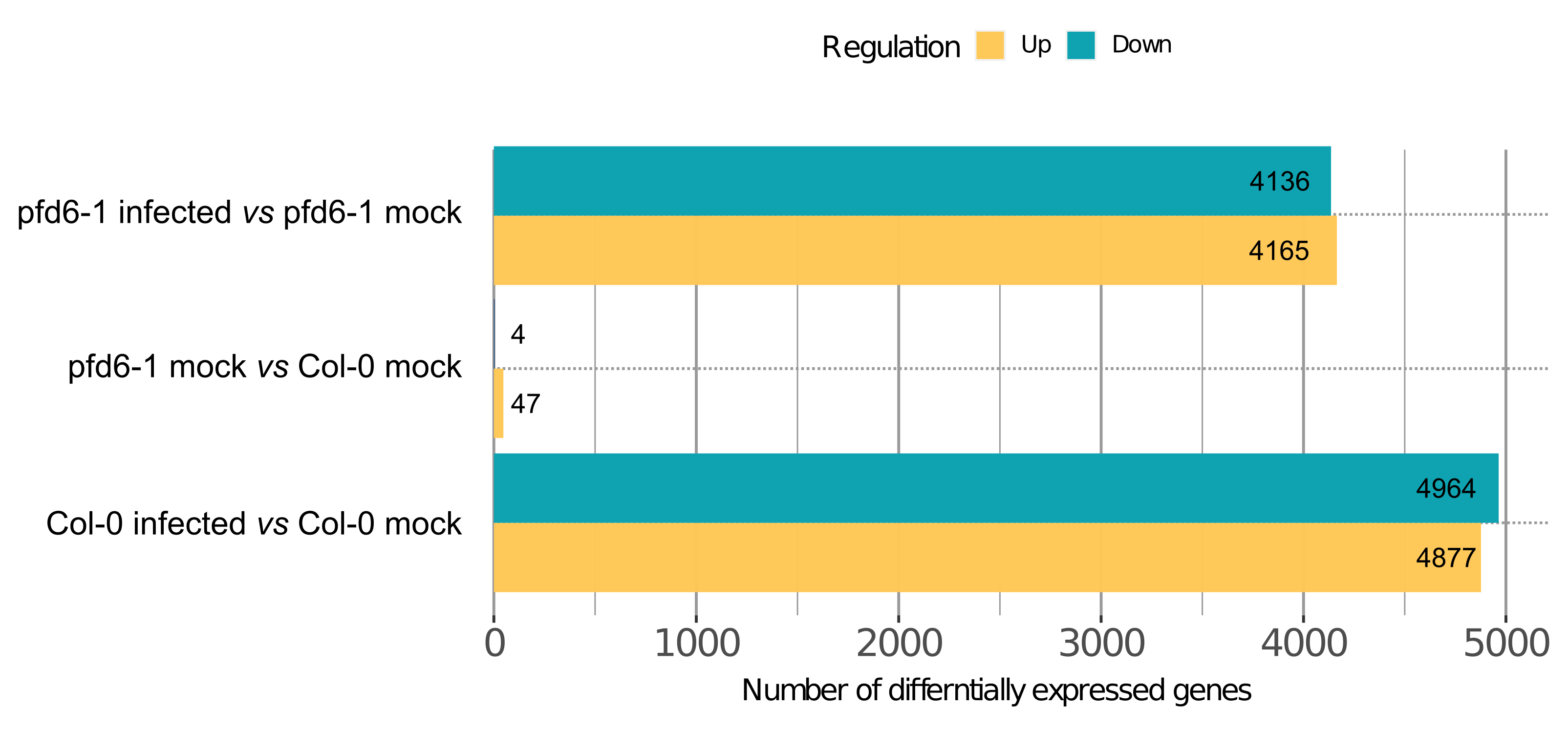

Supplement: Supplementary file 9 — Additional file 9: Fig S6. Differential gene analysis computed with DESeq2 method. [file 12915_2022_1495_MOESM9_ESM.png]

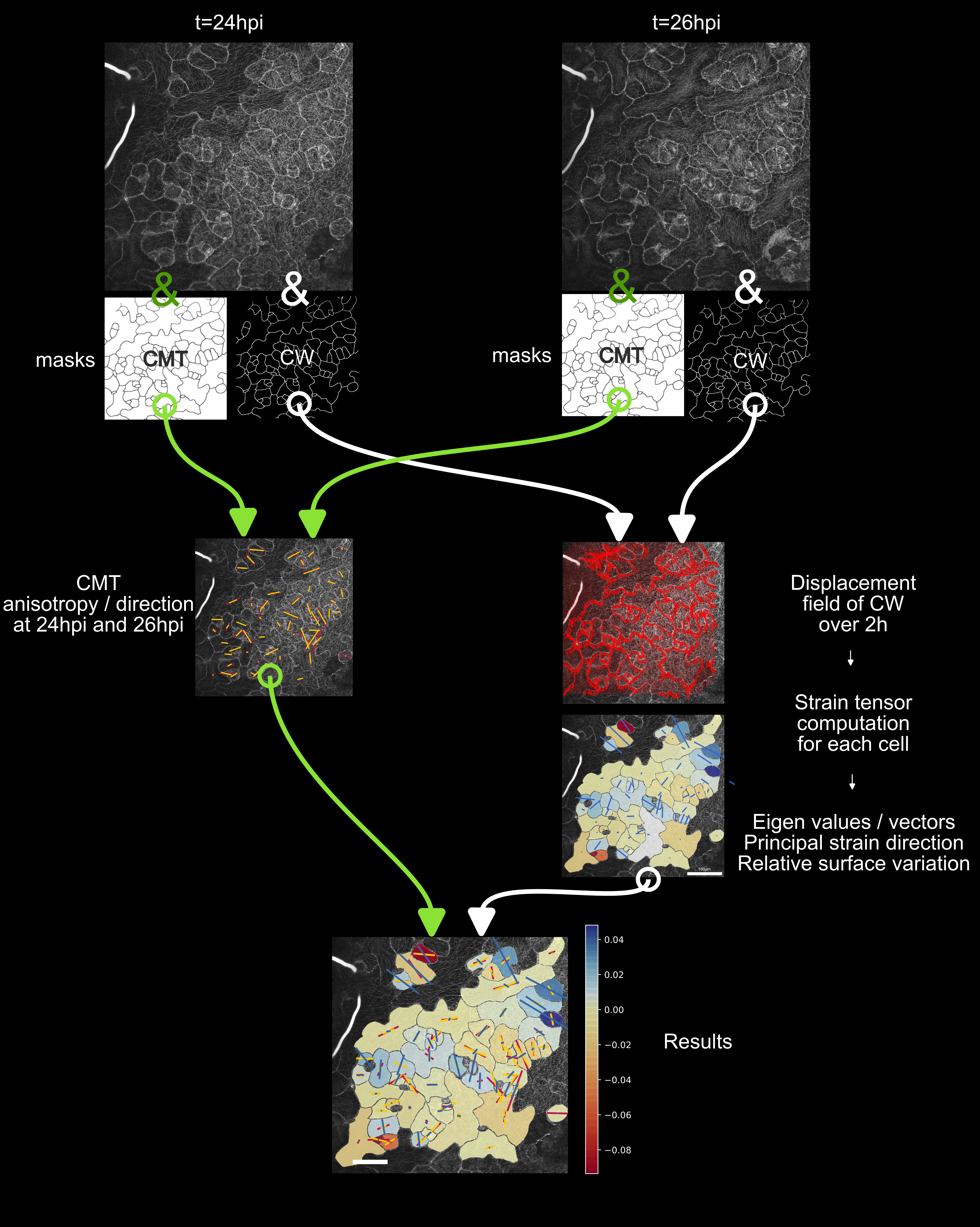

Supplement: Supplementary file 13 — Additional file 13: Fig S7. Image analysis pipeline for the CMTs reorganization and displacement field. [file 12915_2022_1495_MOESM13_ESM.png]

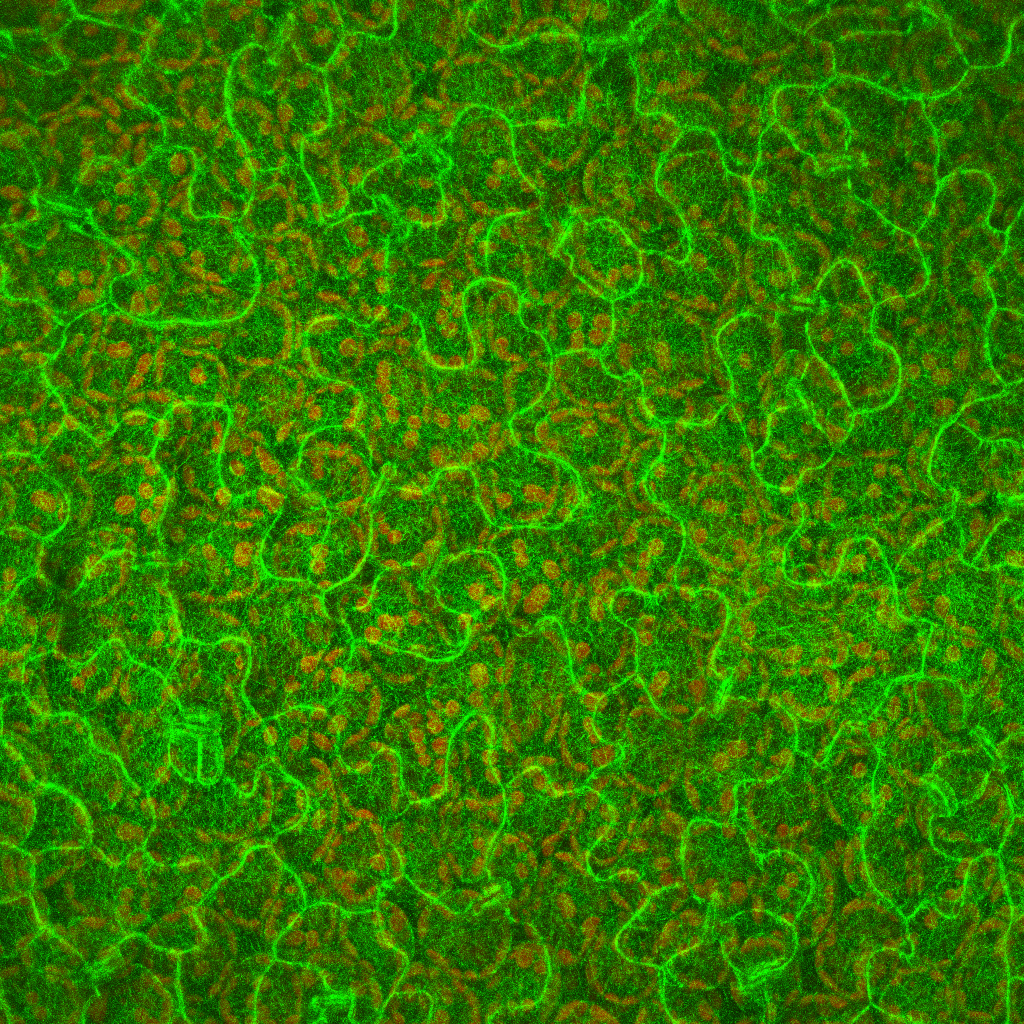

Supplement: Supplementary file 14 — Additional file 14: File S5. Pictures used in this work (raw and analyzed). [file 12915_2022_1495_MOESM14_ESM.xz › Control/C1/T0.png]

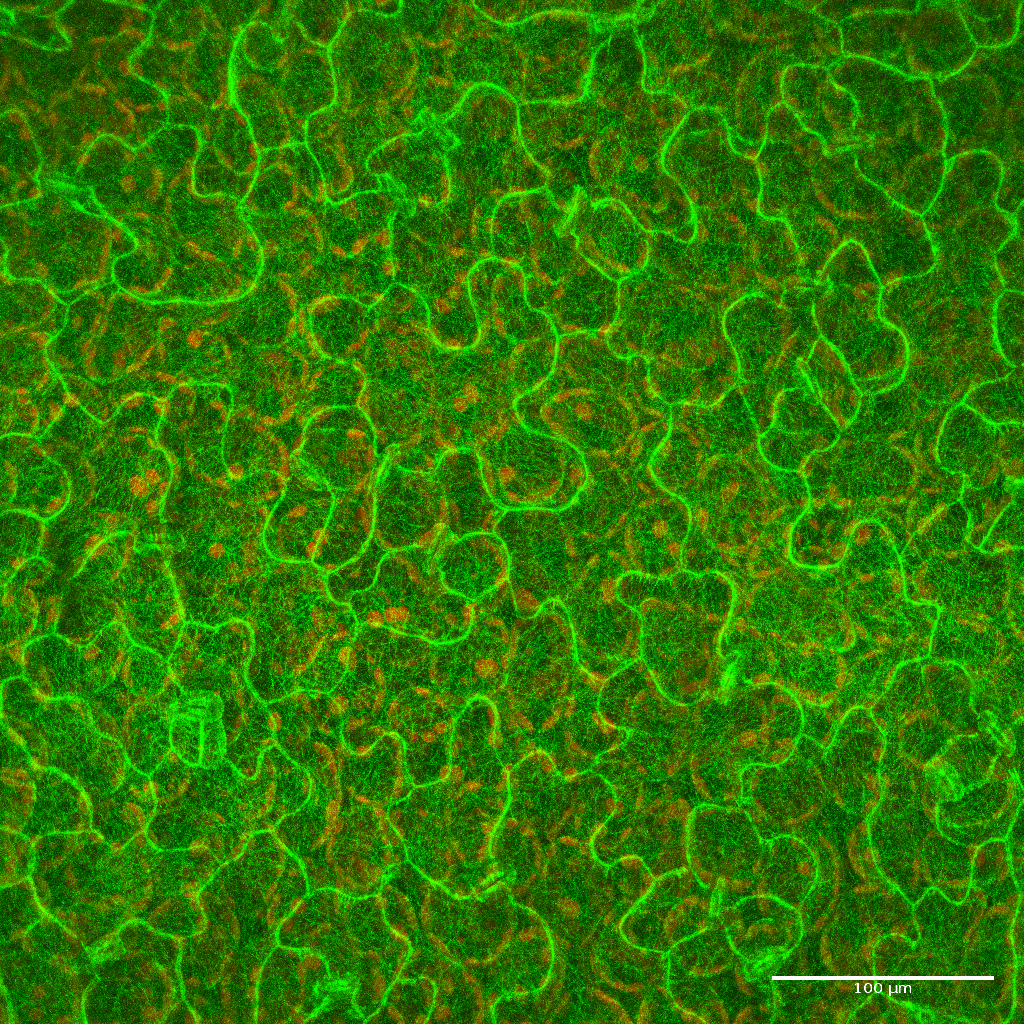

Supplement: Supplementary file 14 — Additional file 14: File S5. Pictures used in this work (raw and analyzed). [file 12915_2022_1495_MOESM14_ESM.xz › Control/C1/T2.png]

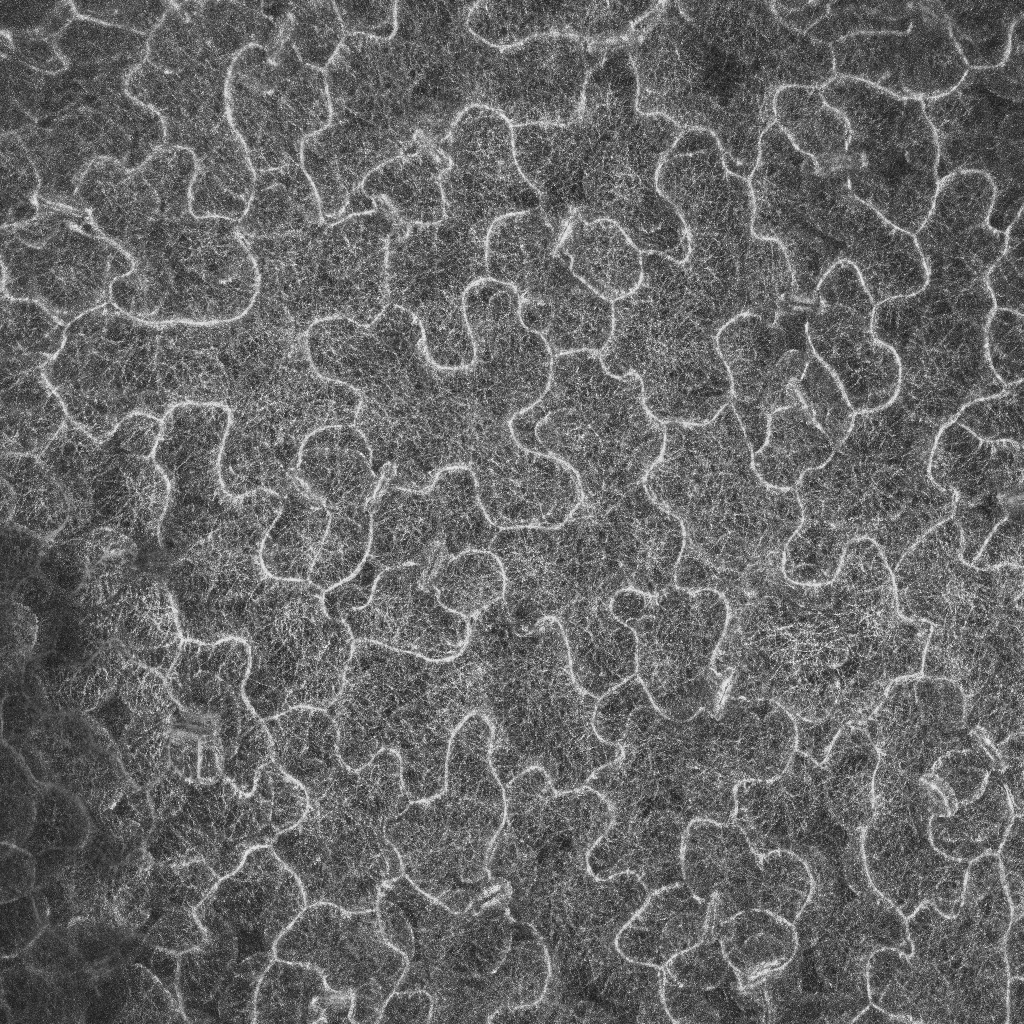

Supplement: Supplementary file 14 — Additional file 14: File S5. Pictures used in this work (raw and analyzed). [file 12915_2022_1495_MOESM14_ESM.xz › Control/C1/0h/grey.tif]

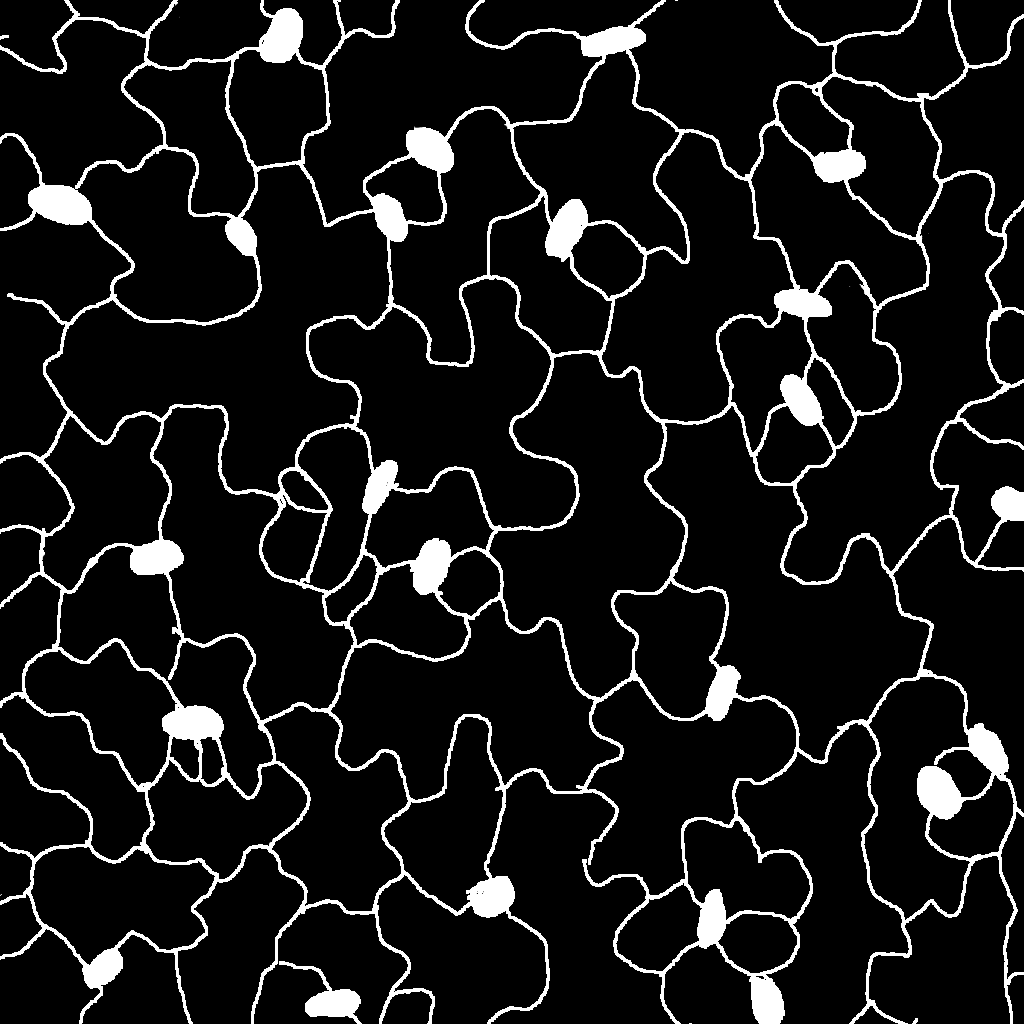

Supplement: Supplementary file 14 — Additional file 14: File S5. Pictures used in this work (raw and analyzed). [file 12915_2022_1495_MOESM14_ESM.xz › Control/C1/0h/mask_checked.tif]

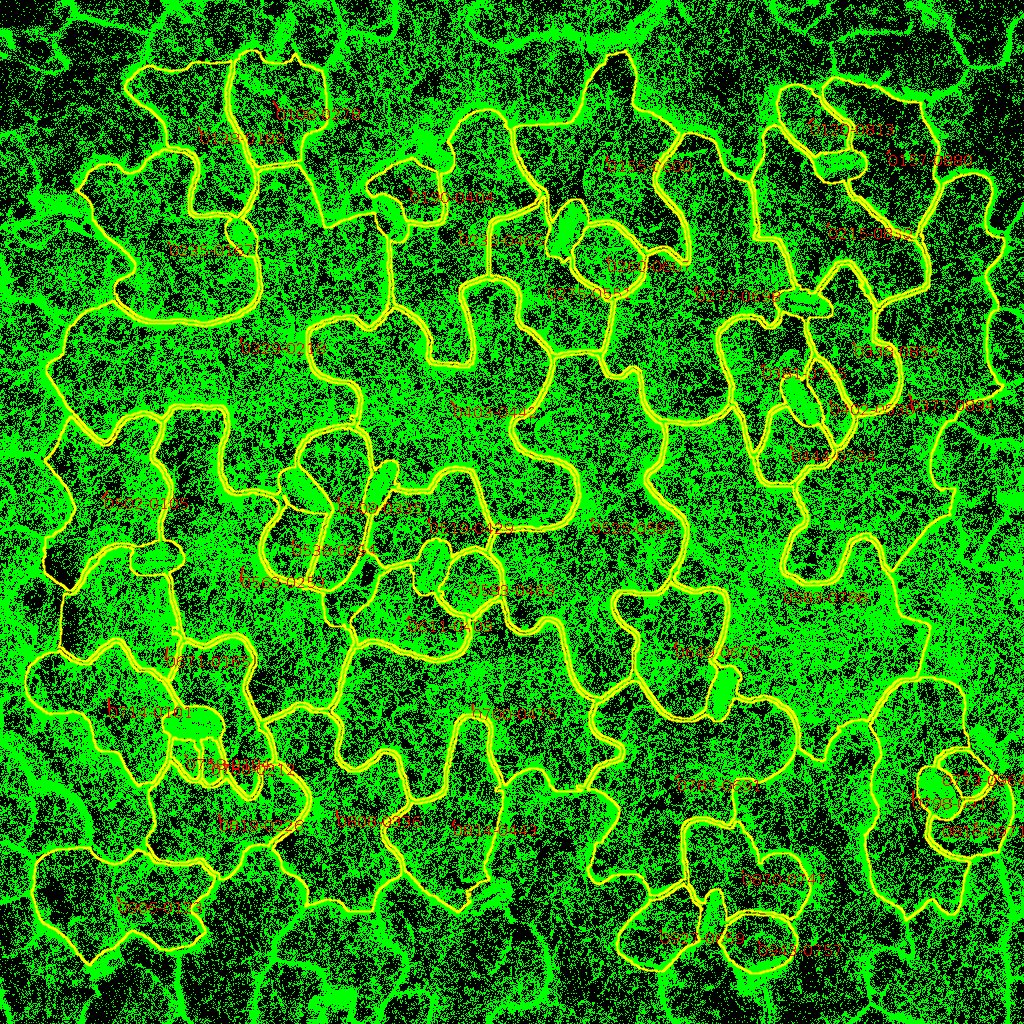

Supplement: Supplementary file 14 — Additional file 14: File S5. Pictures used in this work (raw and analyzed). [file 12915_2022_1495_MOESM14_ESM.xz › Control/C1/0h/T0thres_fib.jpg]

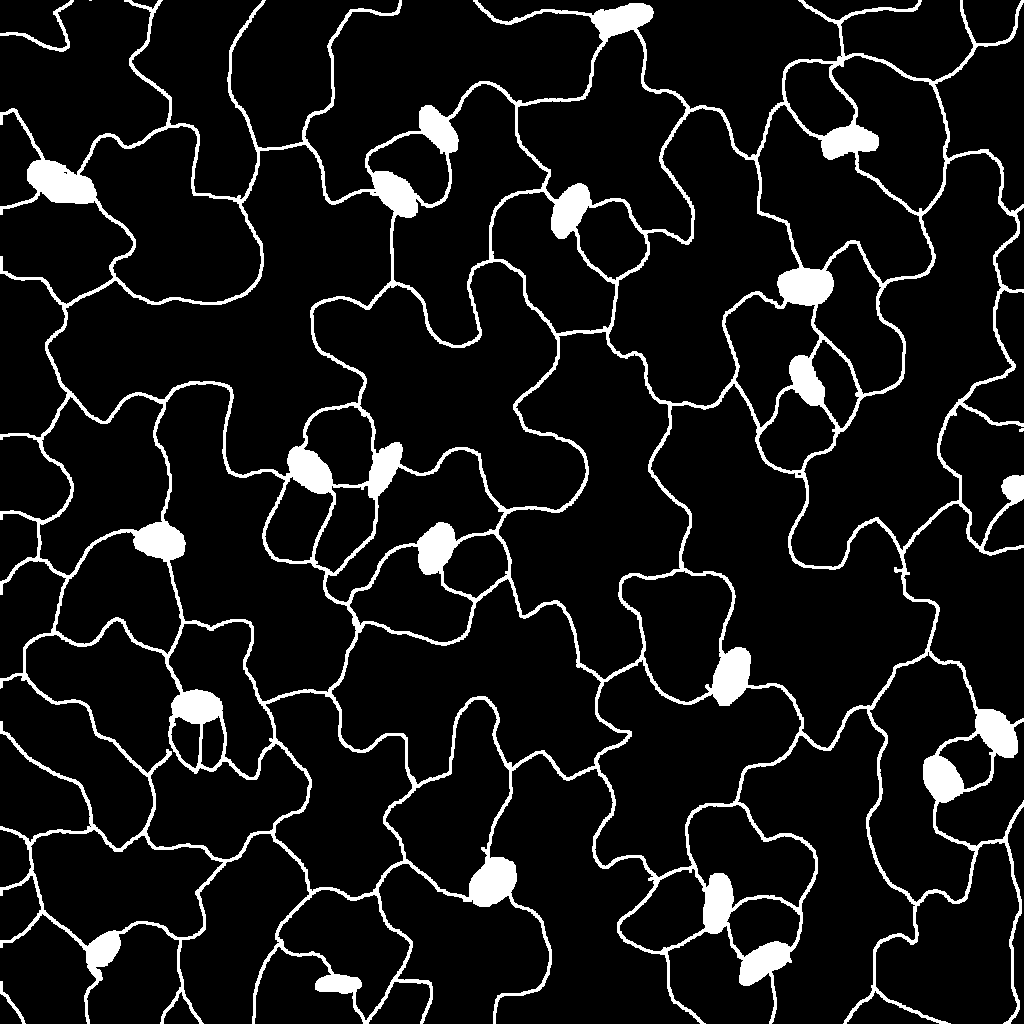

Supplement: Supplementary file 14 — Additional file 14: File S5. Pictures used in this work (raw and analyzed). [file 12915_2022_1495_MOESM14_ESM.xz › Control/C1/2h/mask_checked.tif]

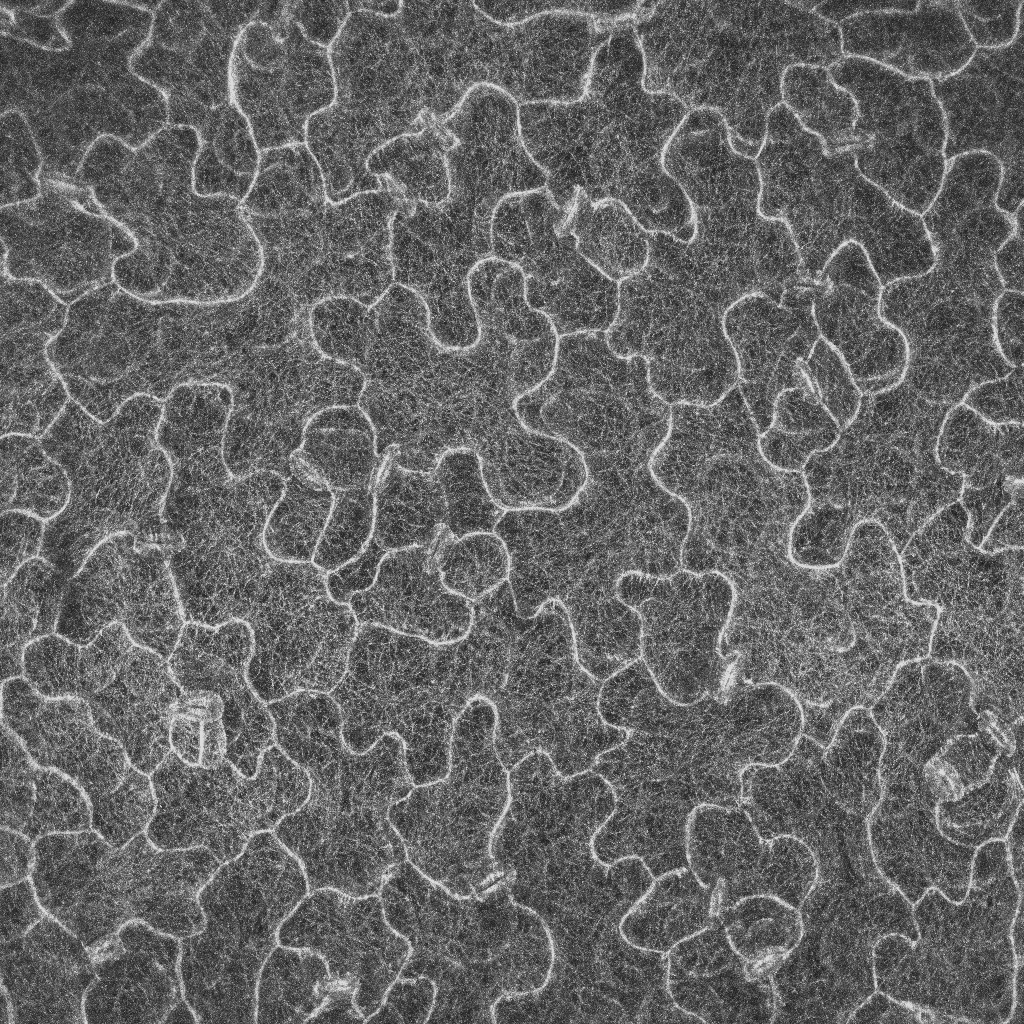

Supplement: Supplementary file 14 — Additional file 14: File S5. Pictures used in this work (raw and analyzed). [file 12915_2022_1495_MOESM14_ESM.xz › Control/C1/2h/grey.tif]

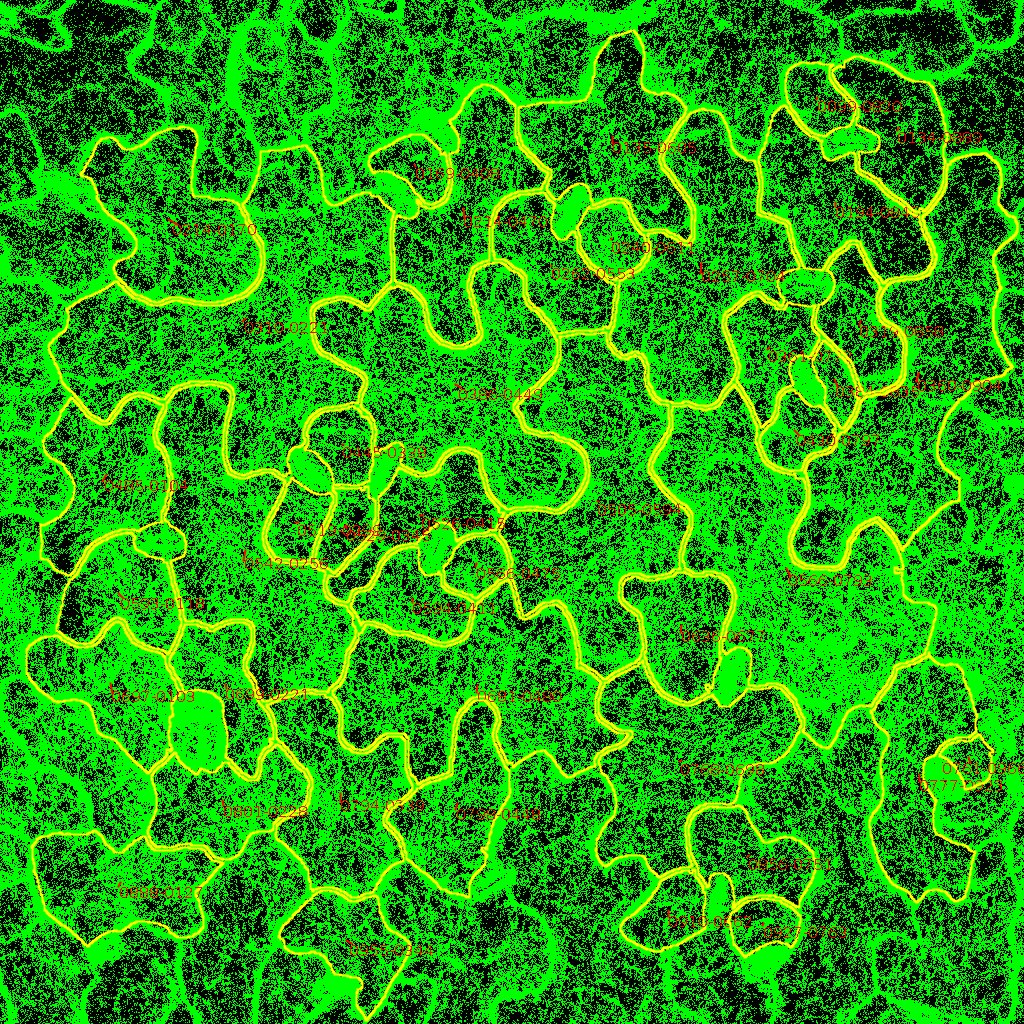

Supplement: Supplementary file 14 — Additional file 14: File S5. Pictures used in this work (raw and analyzed). [file 12915_2022_1495_MOESM14_ESM.xz › Control/C1/2h/T1thres_fib.jpg]

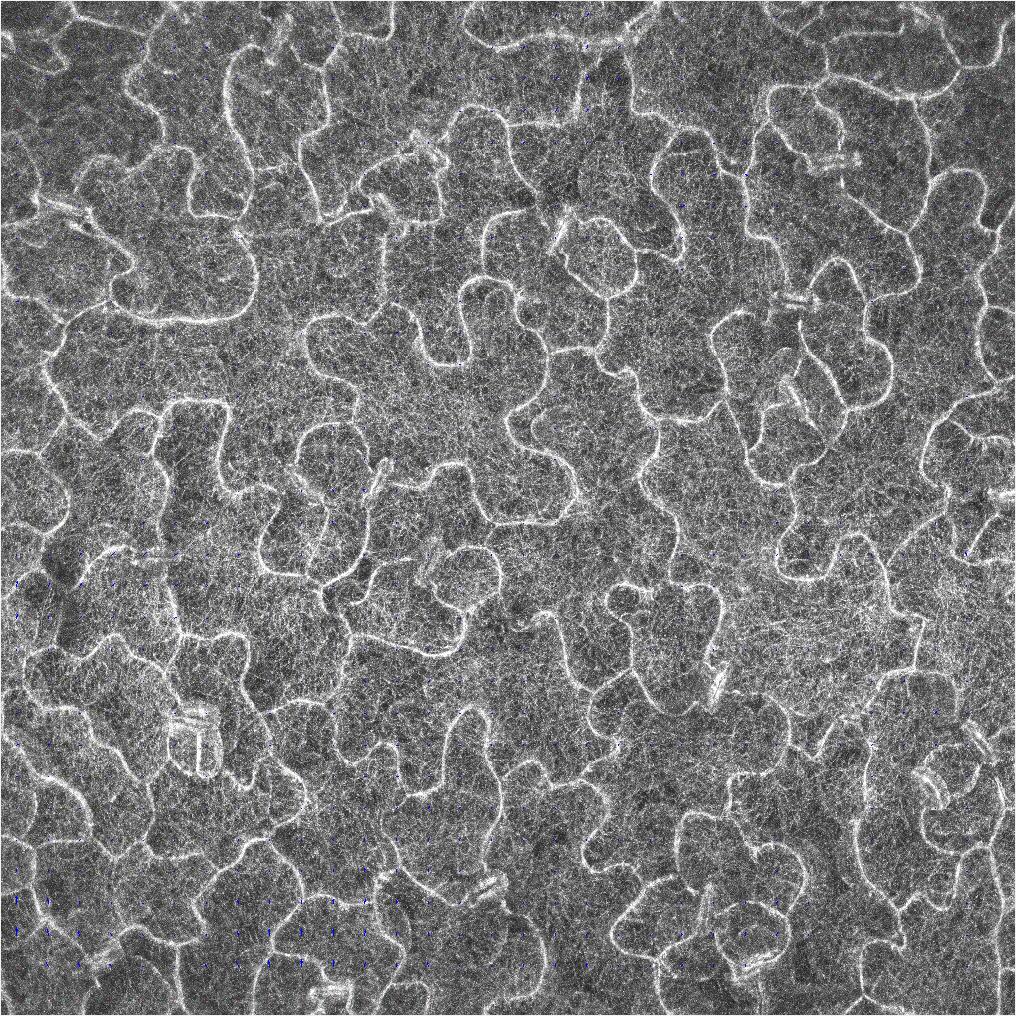

Supplement: Supplementary file 14 — Additional file 14: File S5. Pictures used in this work (raw and analyzed). [file 12915_2022_1495_MOESM14_ESM.xz › Control/C1/KLT_T0_T1/res.jpg]

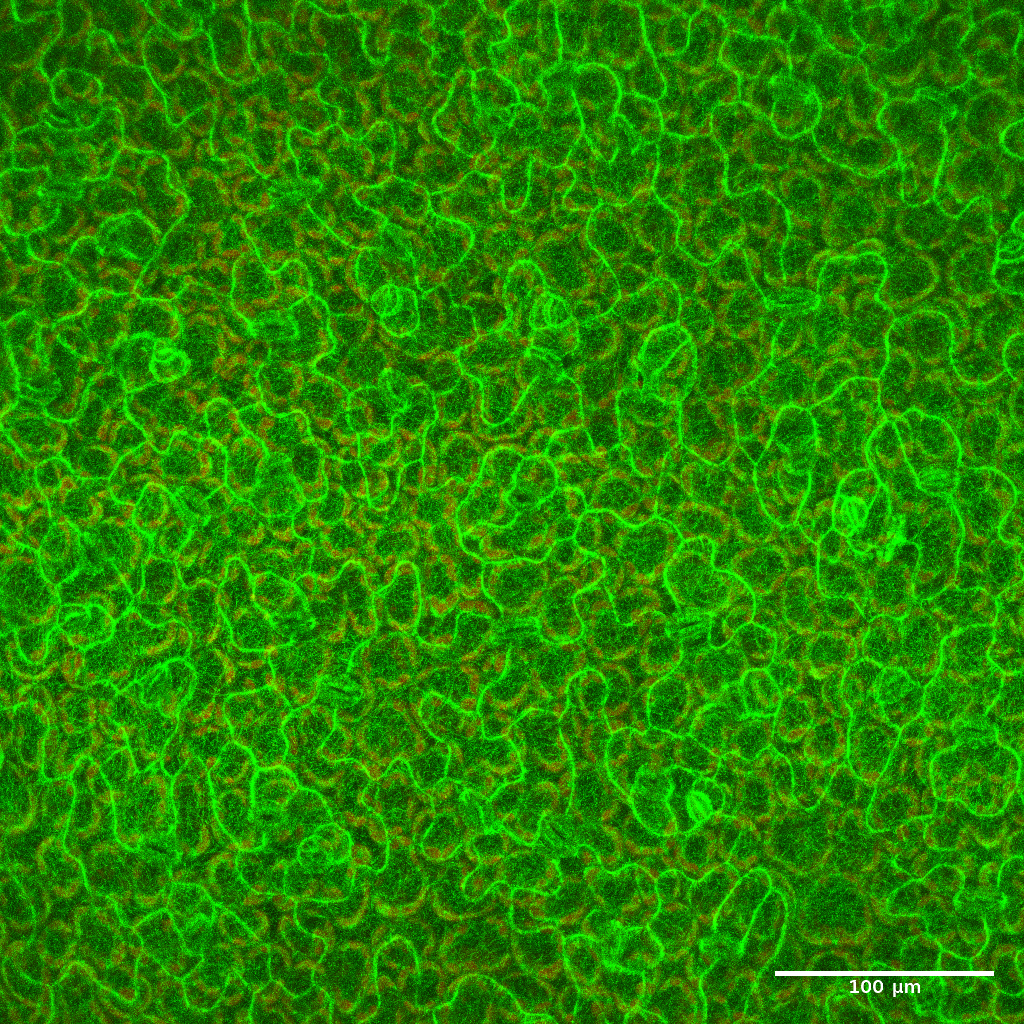

Supplement: Supplementary file 14 — Additional file 14: File S5. Pictures used in this work (raw and analyzed). [file 12915_2022_1495_MOESM14_ESM.xz › Control/C3/T0.tif]

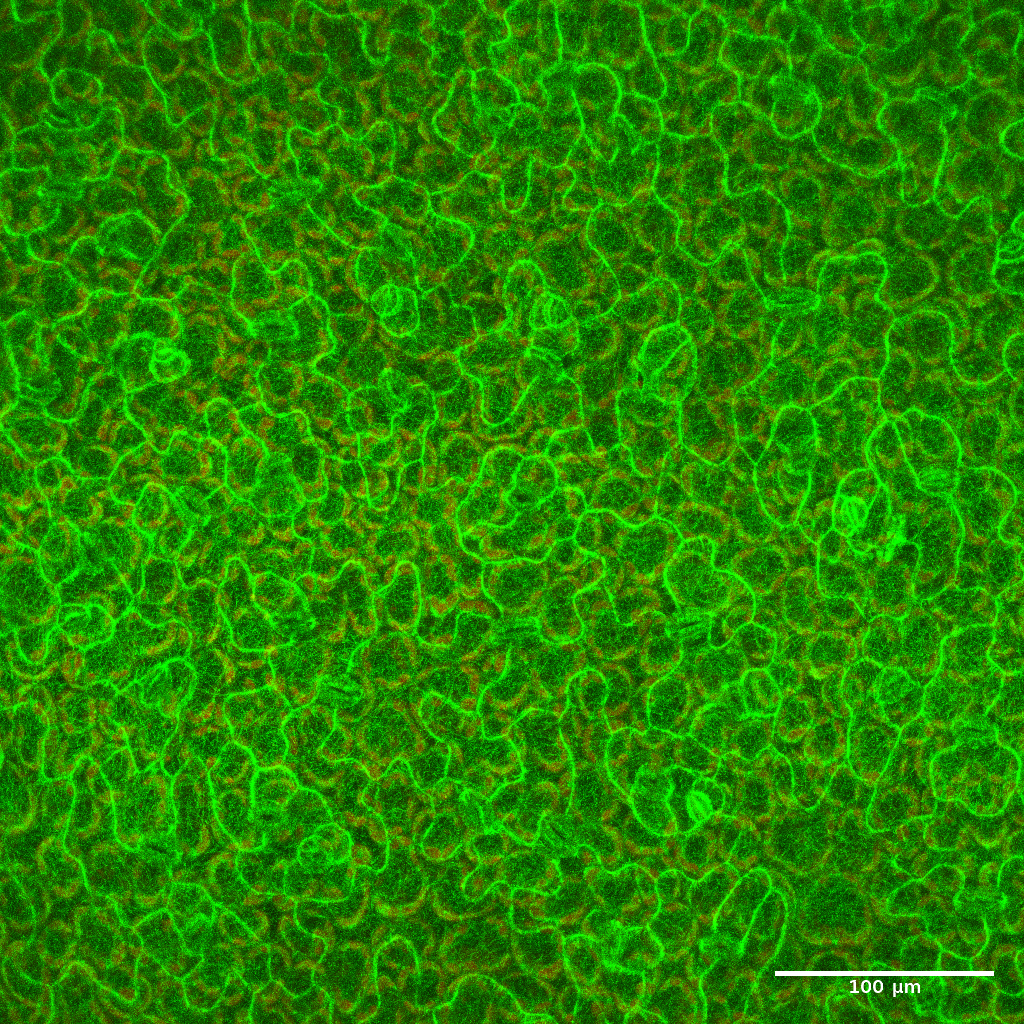

Supplement: Supplementary file 14 — Additional file 14: File S5. Pictures used in this work (raw and analyzed). [file 12915_2022_1495_MOESM14_ESM.xz › Control/C3/T0.png]

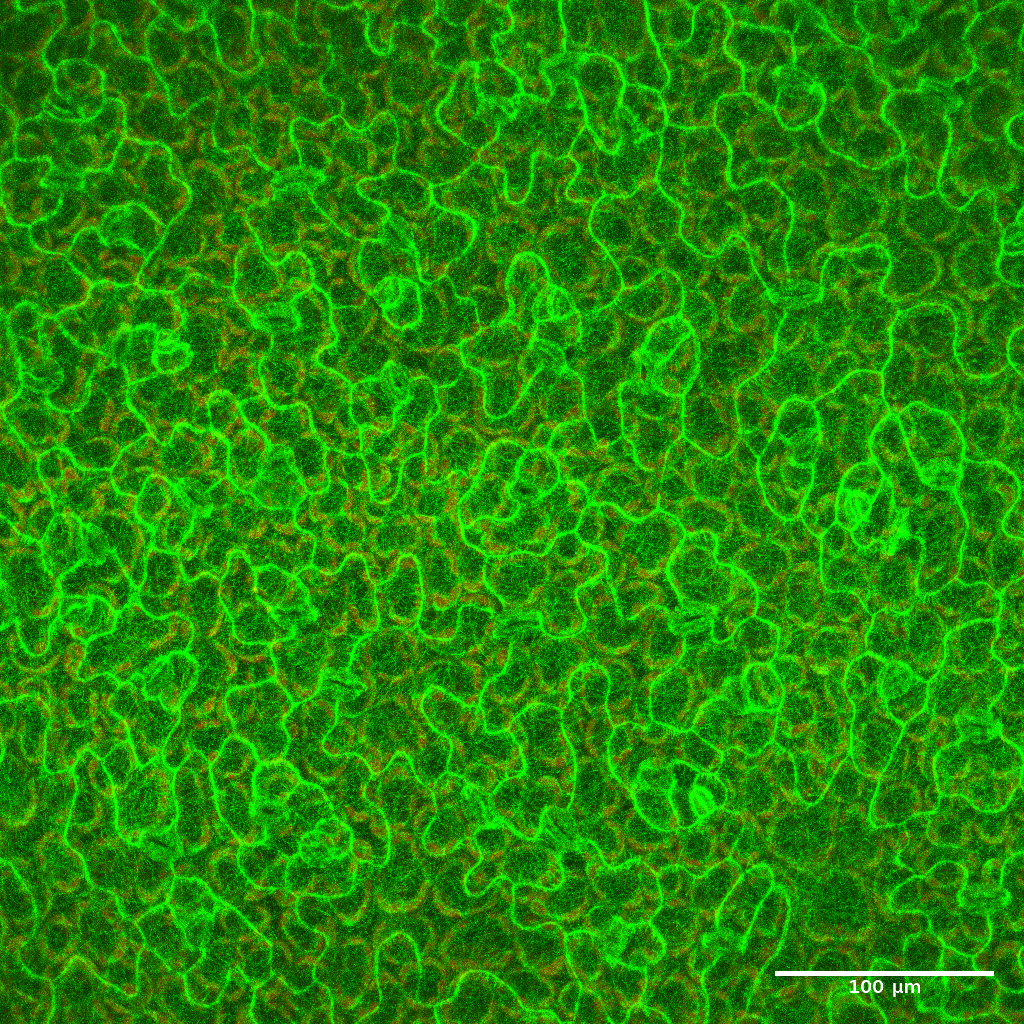

Supplement: Supplementary file 14 — Additional file 14: File S5. Pictures used in this work (raw and analyzed). [file 12915_2022_1495_MOESM14_ESM.xz › Control/C3/T2.png]

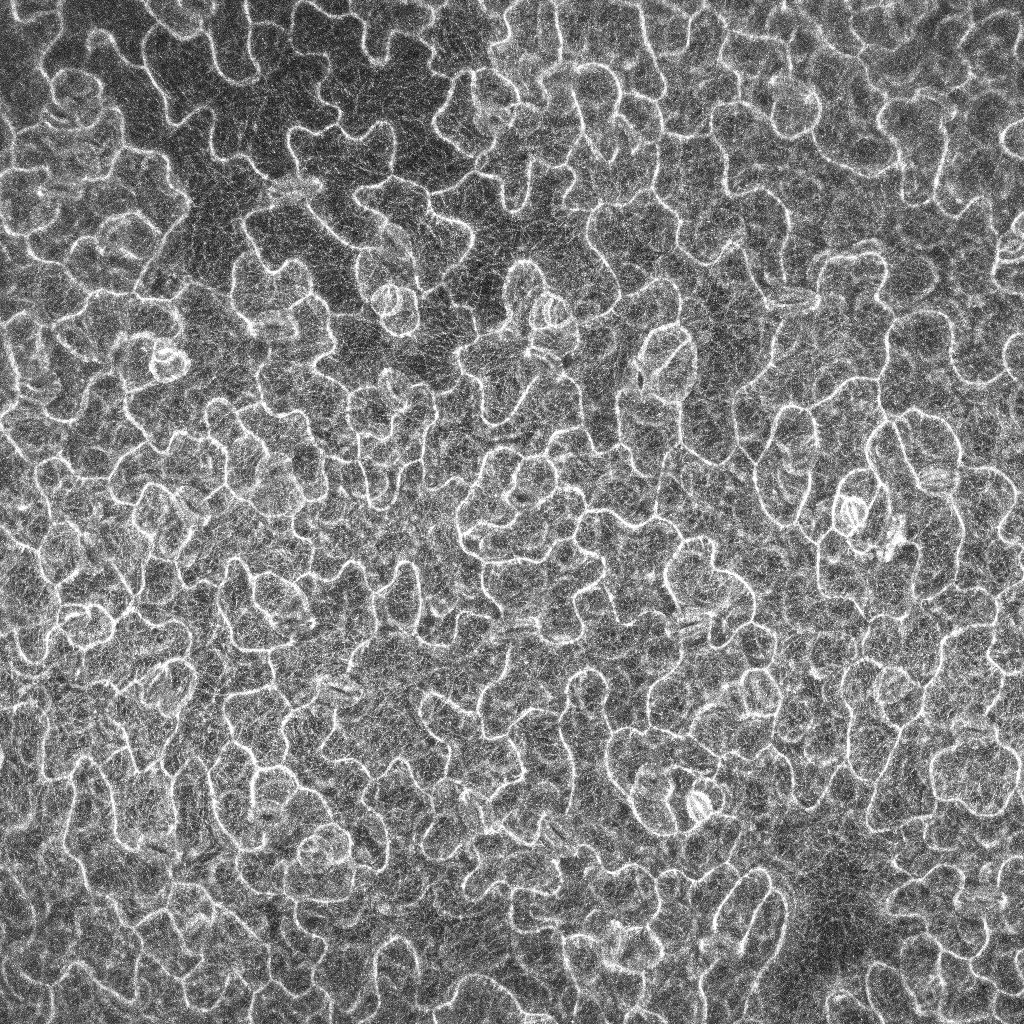

Supplement: Supplementary file 14 — Additional file 14: File S5. Pictures used in this work (raw and analyzed). [file 12915_2022_1495_MOESM14_ESM.xz › Control/C3/0h/grey.tif]

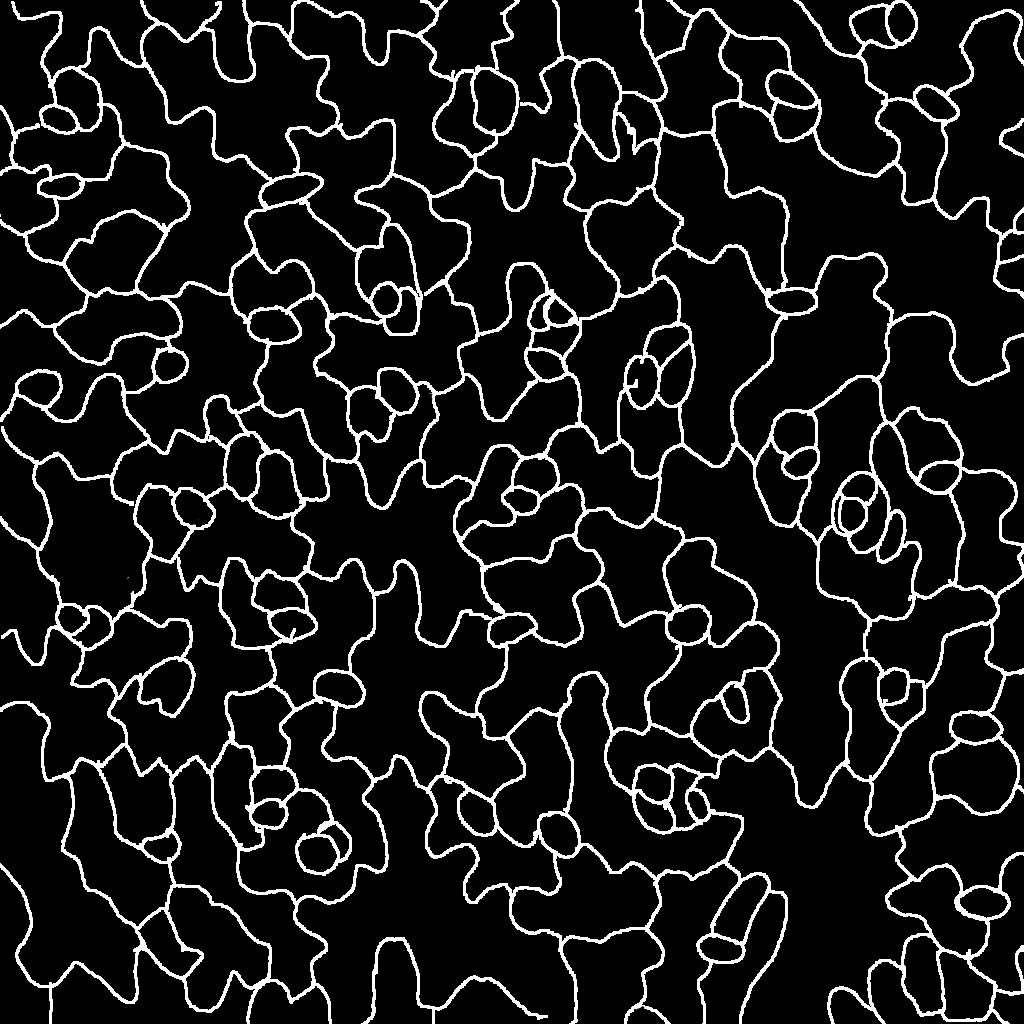

Supplement: Supplementary file 14 — Additional file 14: File S5. Pictures used in this work (raw and analyzed). [file 12915_2022_1495_MOESM14_ESM.xz › Control/C3/0h/mask_checked.tif]

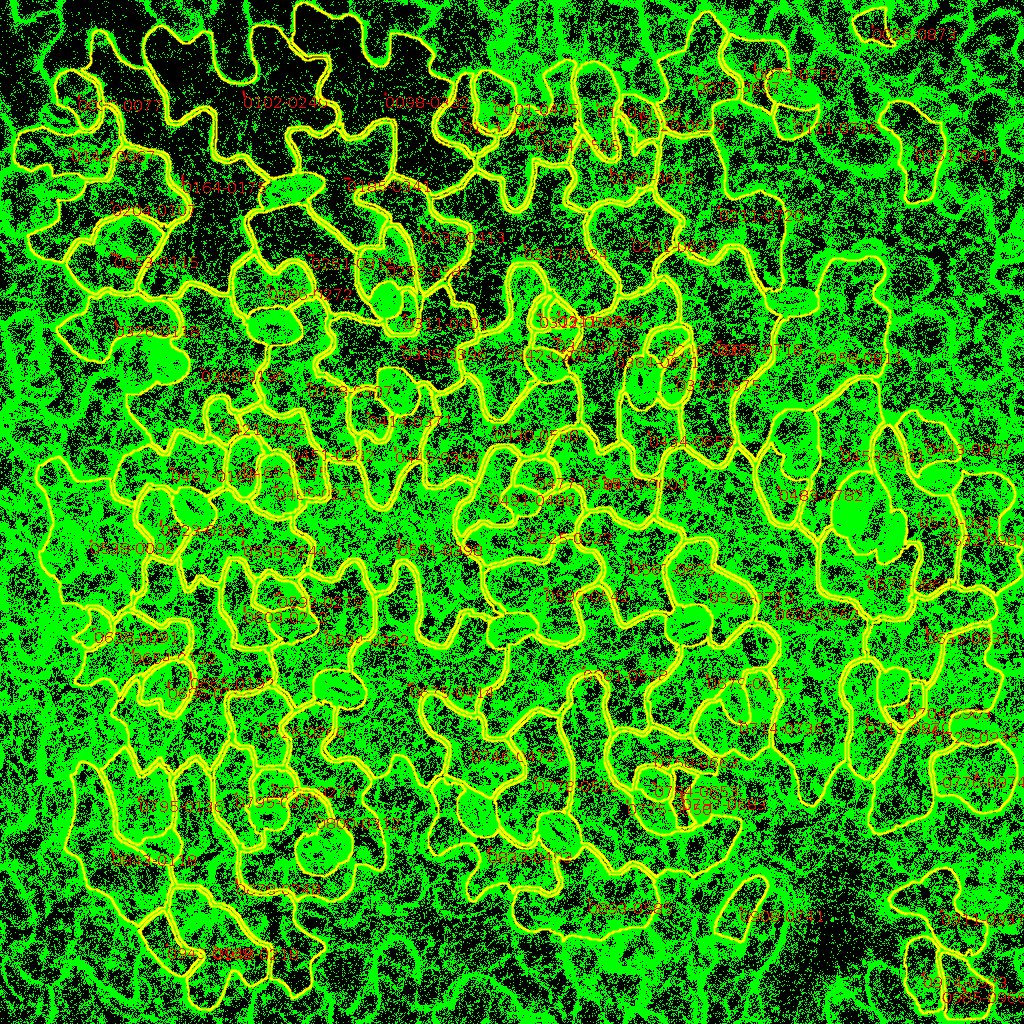

Supplement: Supplementary file 14 — Additional file 14: File S5. Pictures used in this work (raw and analyzed). [file 12915_2022_1495_MOESM14_ESM.xz › Control/C3/0h/T0thres_fib.jpg]

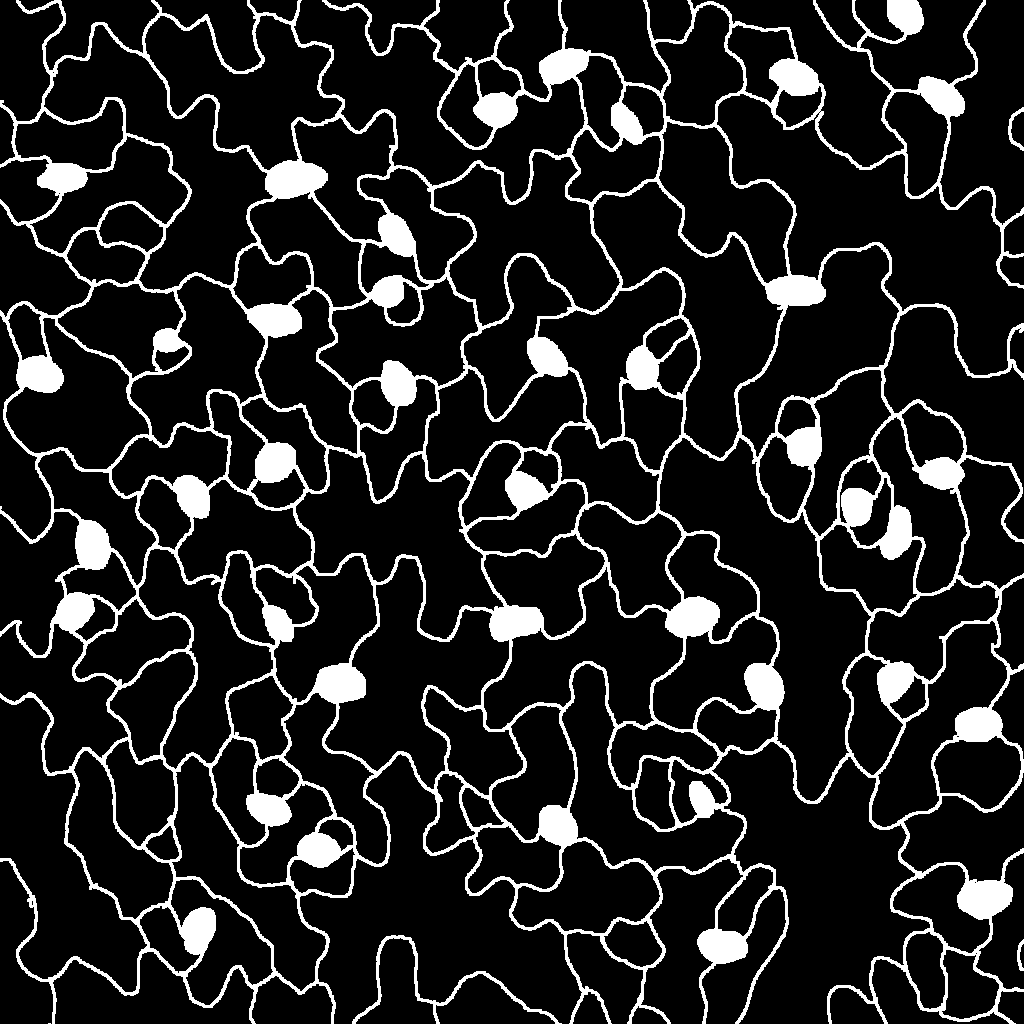

Supplement: Supplementary file 14 — Additional file 14: File S5. Pictures used in this work (raw and analyzed). [file 12915_2022_1495_MOESM14_ESM.xz › Control/C3/2h/mack_checked.tif]

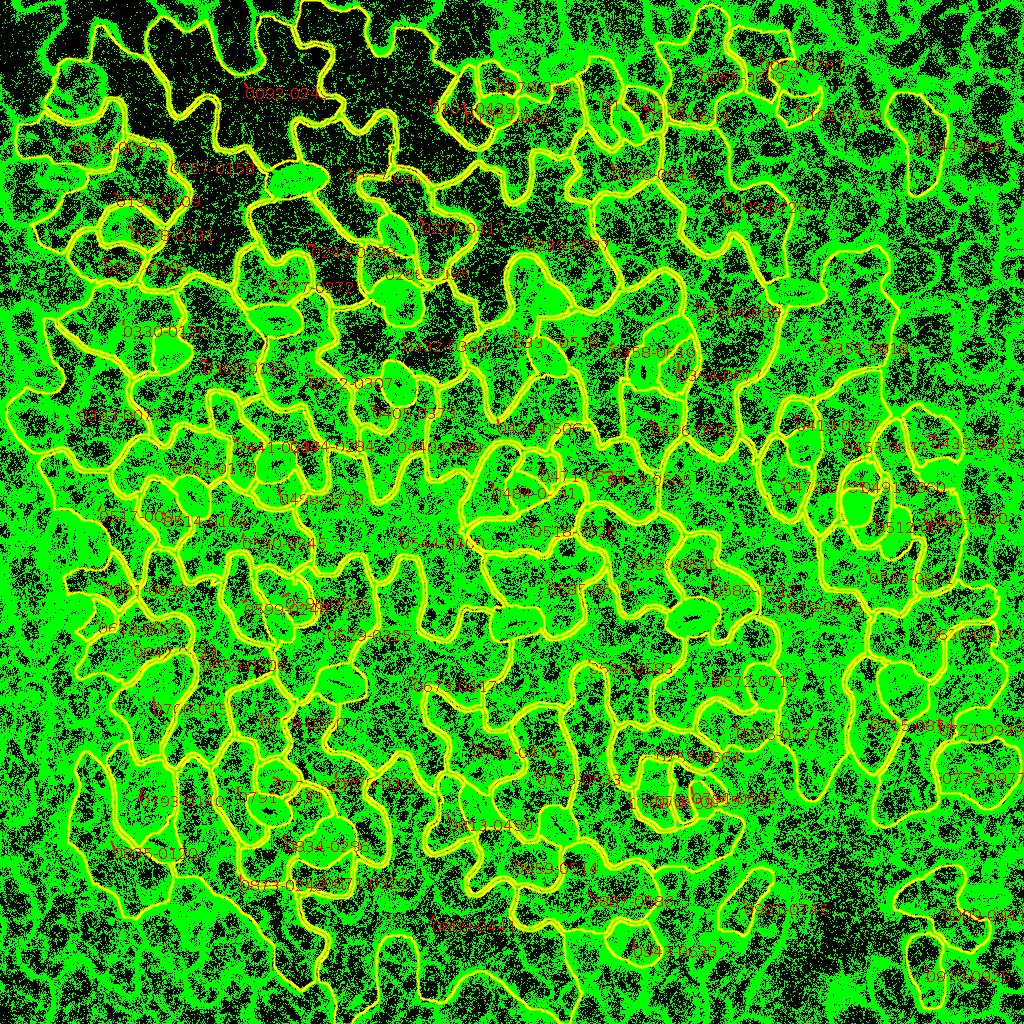

Supplement: Supplementary file 14 — Additional file 14: File S5. Pictures used in this work (raw and analyzed). [file 12915_2022_1495_MOESM14_ESM.xz › Control/C3/2h/T1thres_fib.jpg]

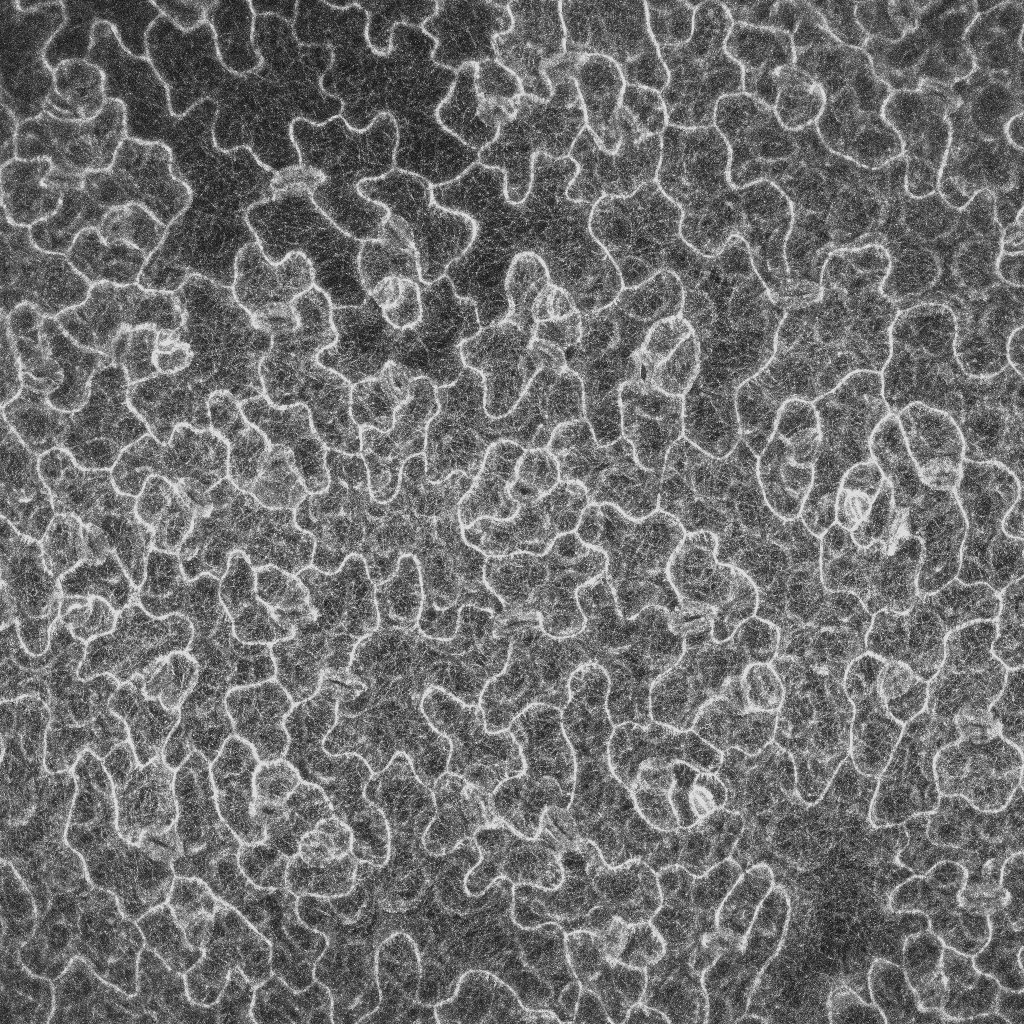

Supplement: Supplementary file 14 — Additional file 14: File S5. Pictures used in this work (raw and analyzed). [file 12915_2022_1495_MOESM14_ESM.xz › Control/C3/2h/grey.tif]

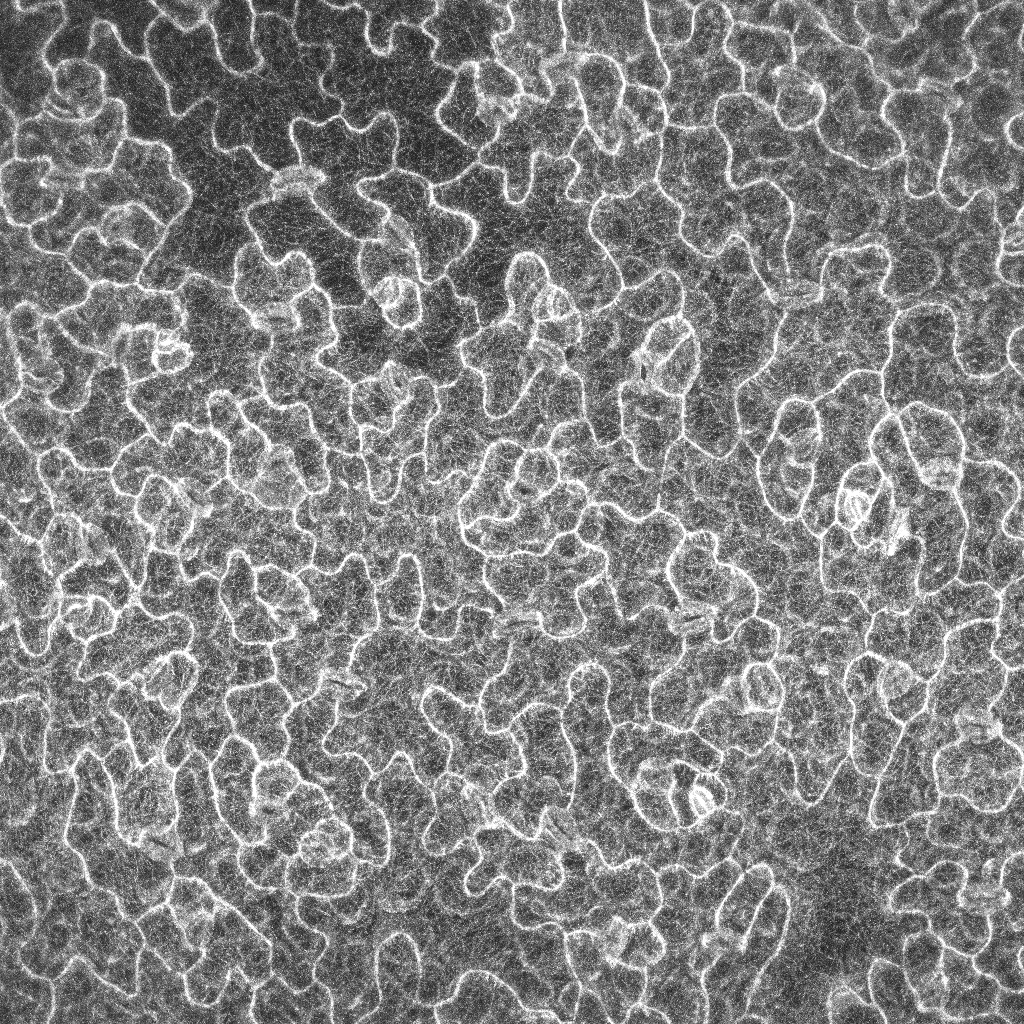

Supplement: Supplementary file 14 — Additional file 14: File S5. Pictures used in this work (raw and analyzed). [file 12915_2022_1495_MOESM14_ESM.xz › Control/C3/KLT_T0_T1/T_1.tif]

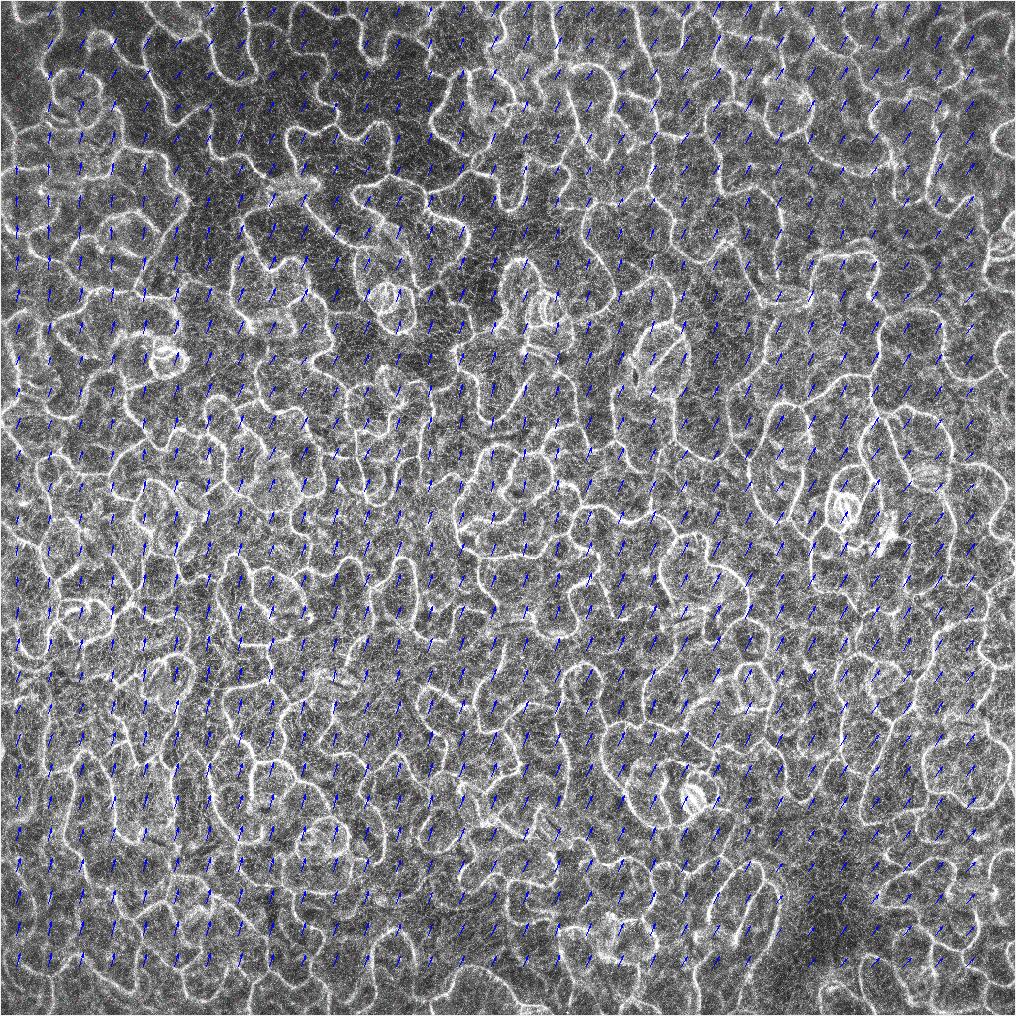

Supplement: Supplementary file 14 — Additional file 14: File S5. Pictures used in this work (raw and analyzed). [file 12915_2022_1495_MOESM14_ESM.xz › Control/C3/KLT_T0_T1/res.jpg]

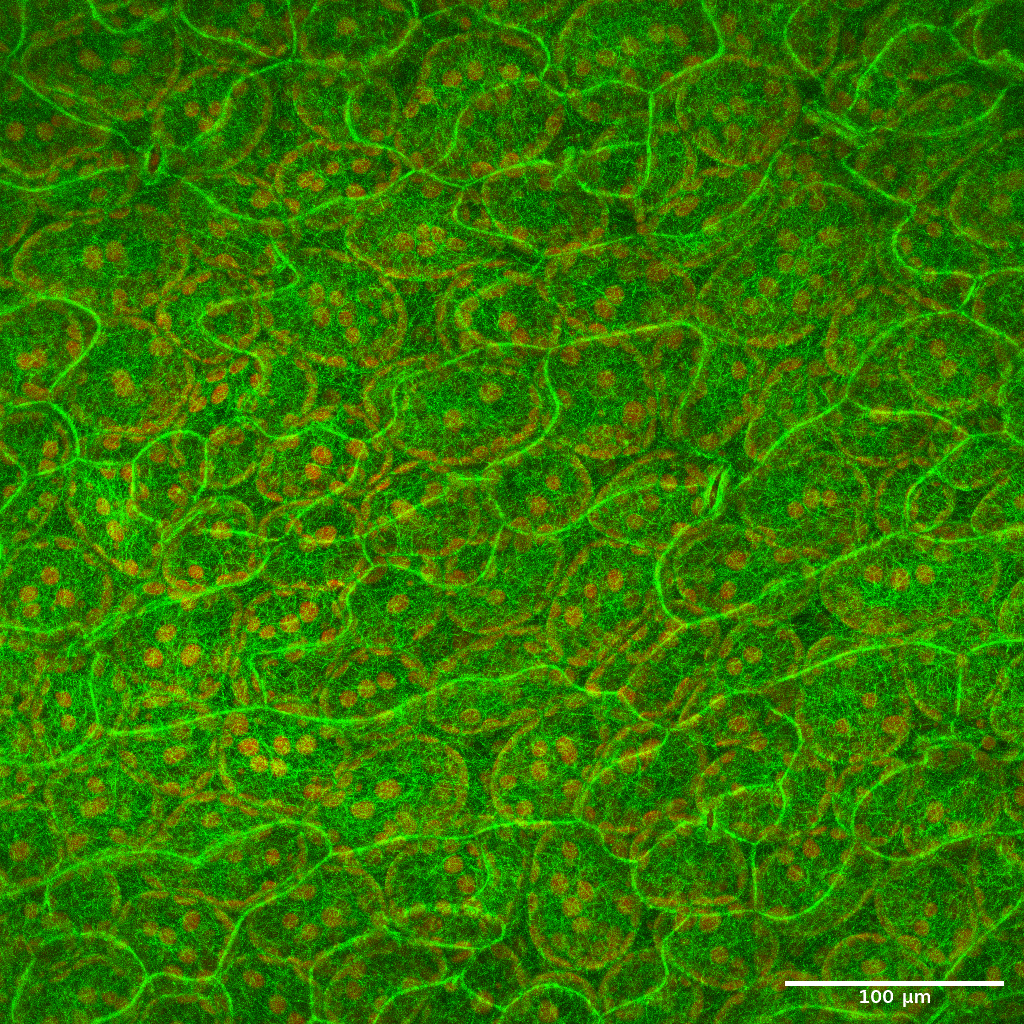

Supplement: Supplementary file 14 — Additional file 14: File S5. Pictures used in this work (raw and analyzed). [file 12915_2022_1495_MOESM14_ESM.xz › Control/C2/T0.tif]

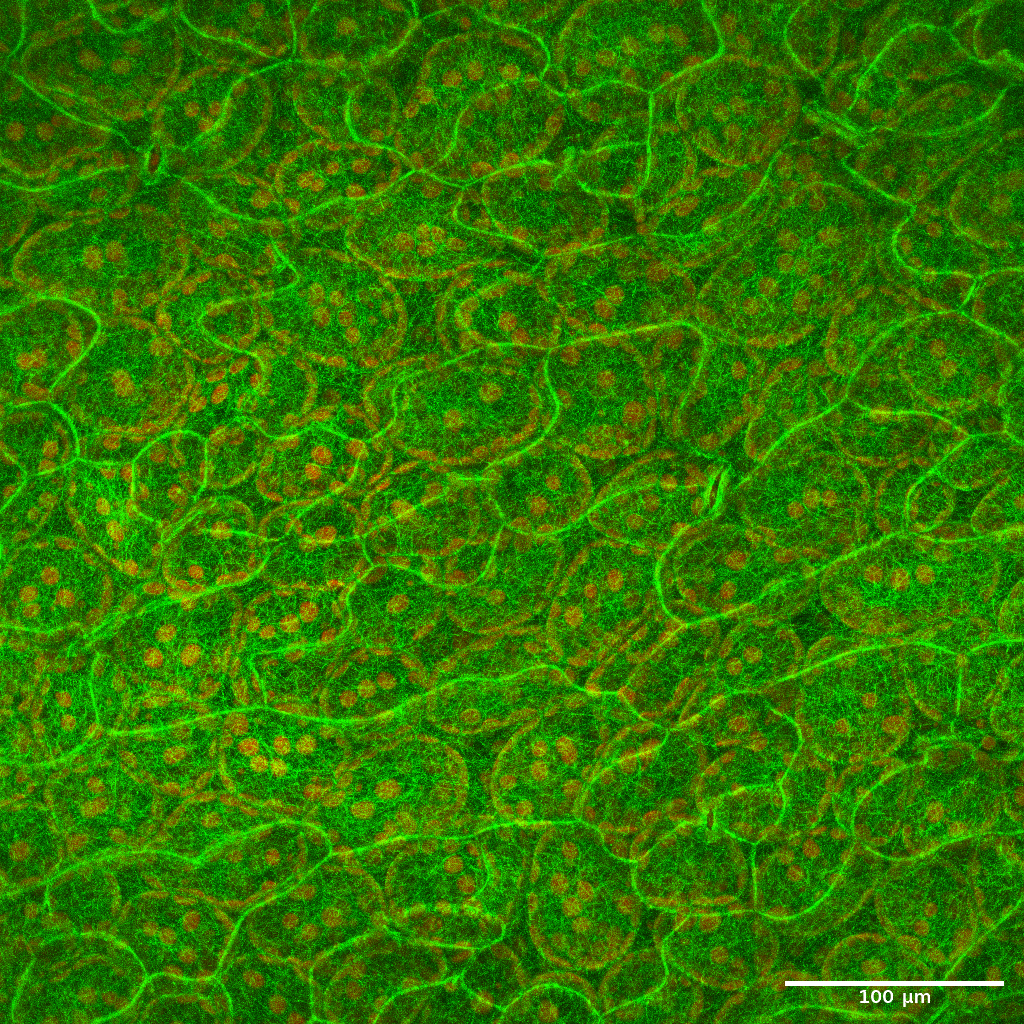

Supplement: Supplementary file 14 — Additional file 14: File S5. Pictures used in this work (raw and analyzed). [file 12915_2022_1495_MOESM14_ESM.xz › Control/C2/T0.png]

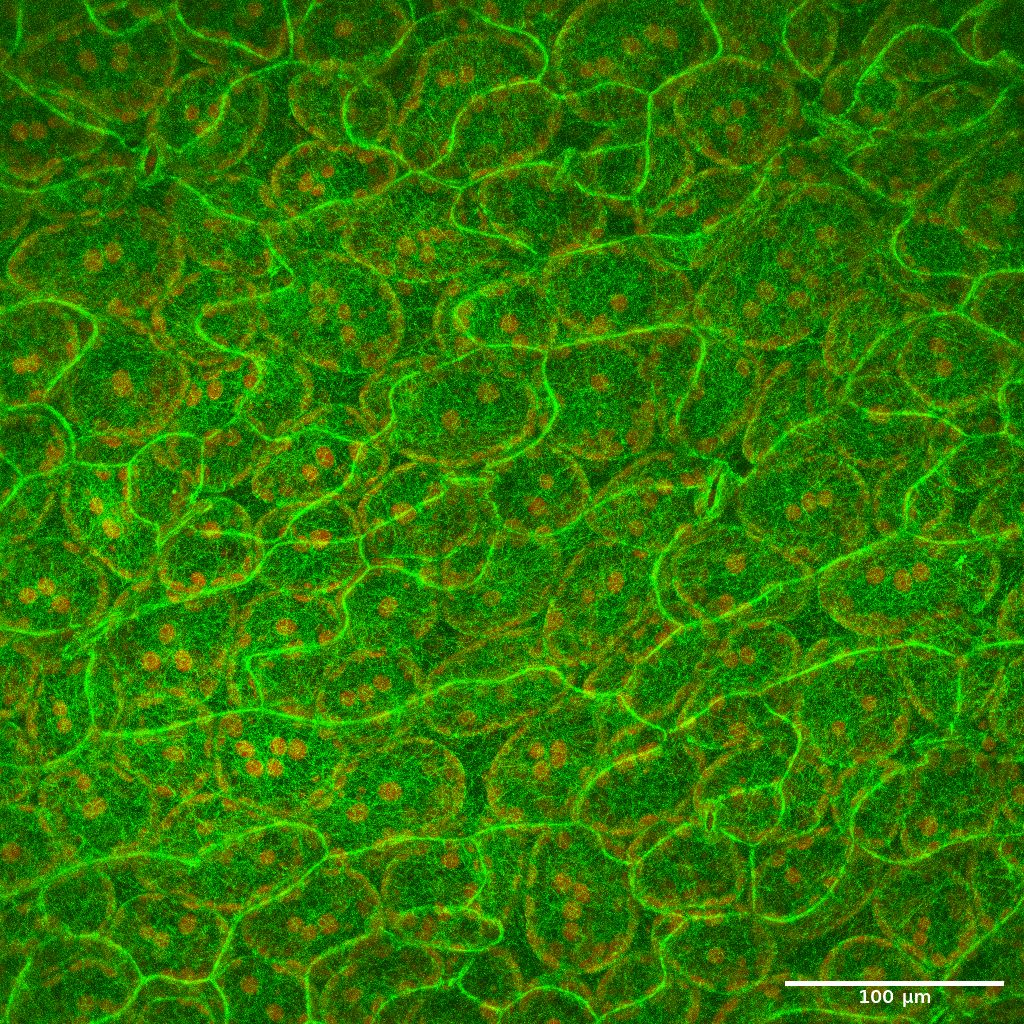

Supplement: Supplementary file 14 — Additional file 14: File S5. Pictures used in this work (raw and analyzed). [file 12915_2022_1495_MOESM14_ESM.xz › Control/C2/T2.png]

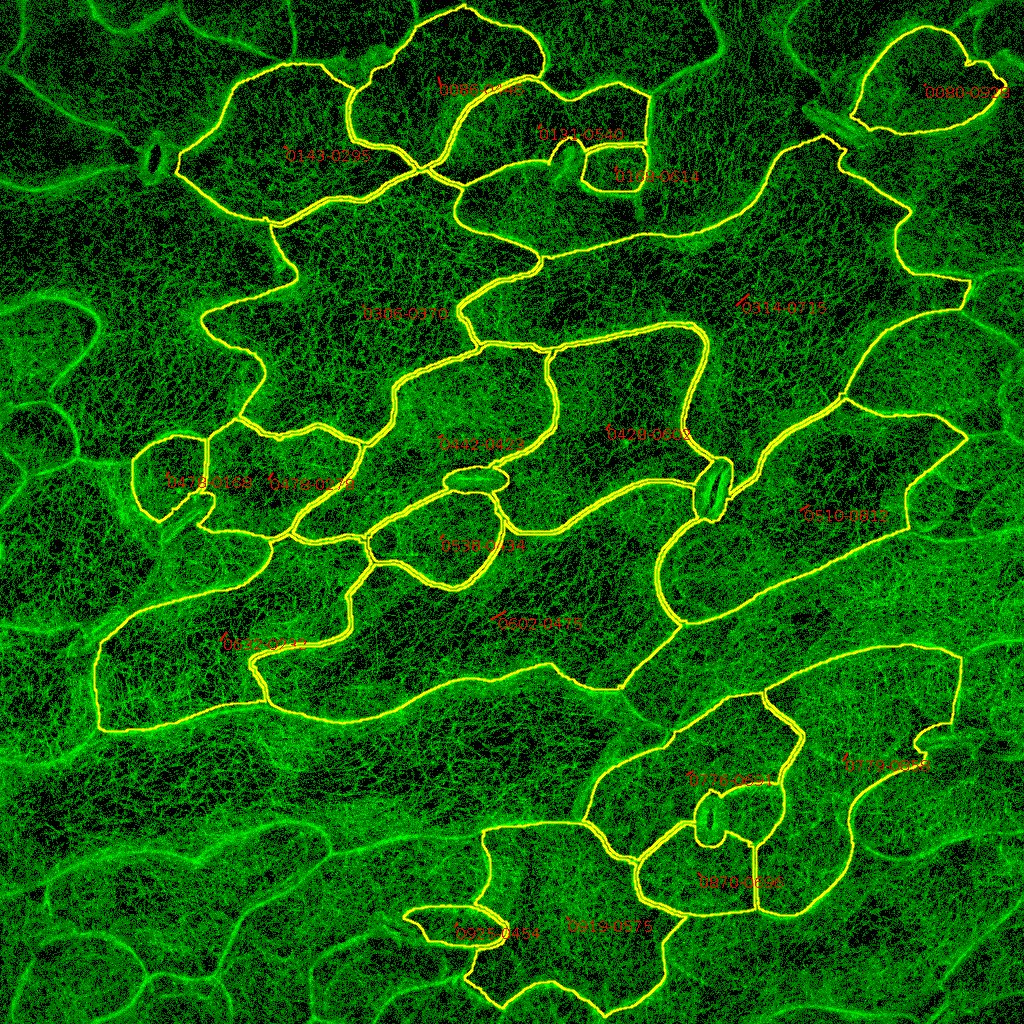

Supplement: Supplementary file 14 — Additional file 14: File S5. Pictures used in this work (raw and analyzed). [file 12915_2022_1495_MOESM14_ESM.xz › Control/C2/0h/T0thres_fib.jpg]

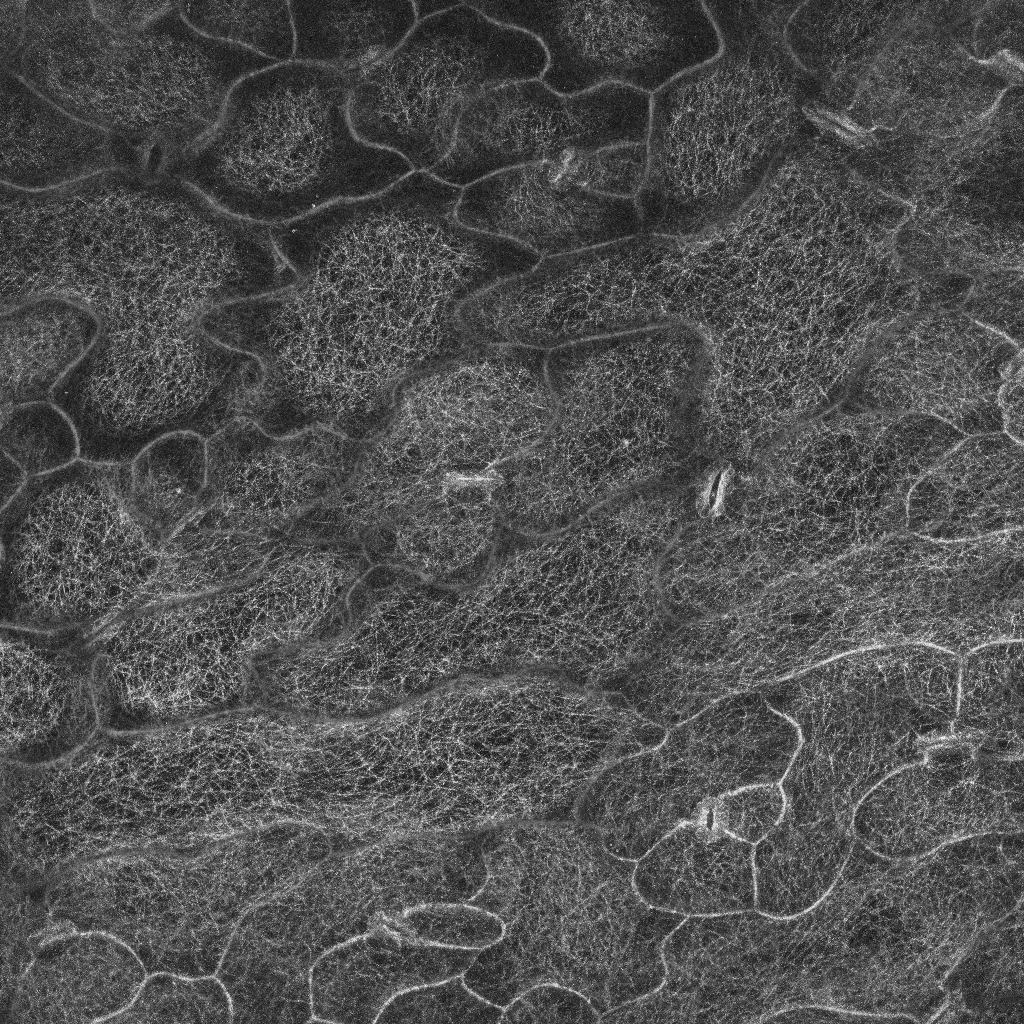

Supplement: Supplementary file 14 — Additional file 14: File S5. Pictures used in this work (raw and analyzed). [file 12915_2022_1495_MOESM14_ESM.xz › Control/C2/0h/grey.tif]

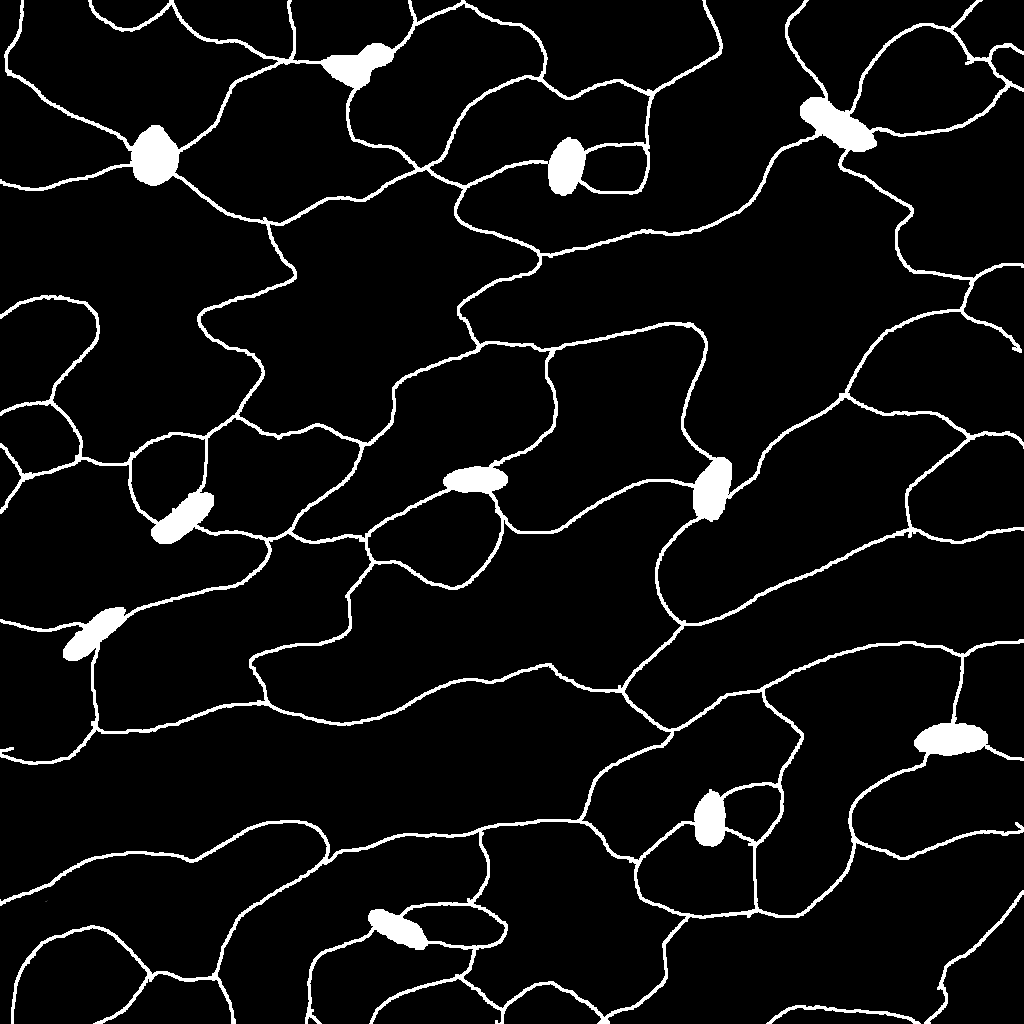

Supplement: Supplementary file 14 — Additional file 14: File S5. Pictures used in this work (raw and analyzed). [file 12915_2022_1495_MOESM14_ESM.xz › Control/C2/0h/mask_checked.tif]

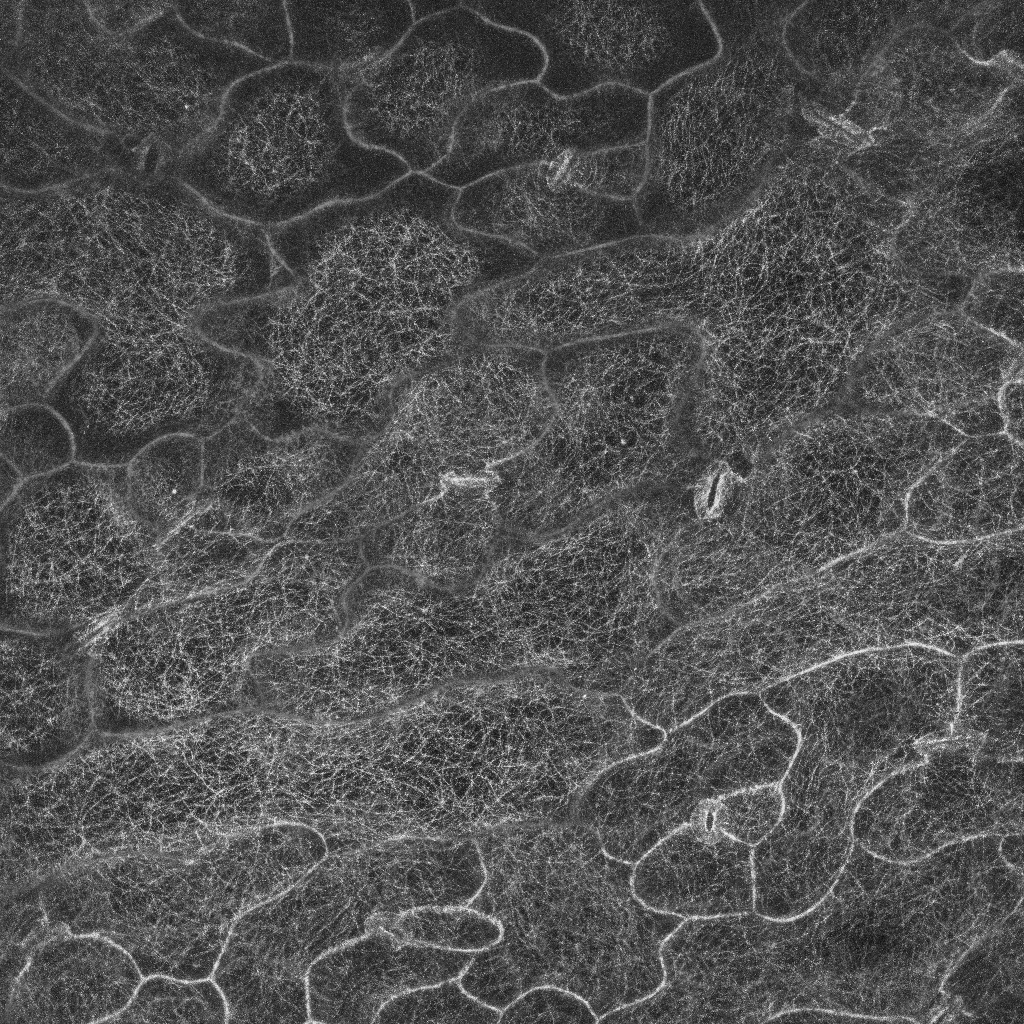

Supplement: Supplementary file 14 — Additional file 14: File S5. Pictures used in this work (raw and analyzed). [file 12915_2022_1495_MOESM14_ESM.xz › Control/C2/2h/grey.tif]

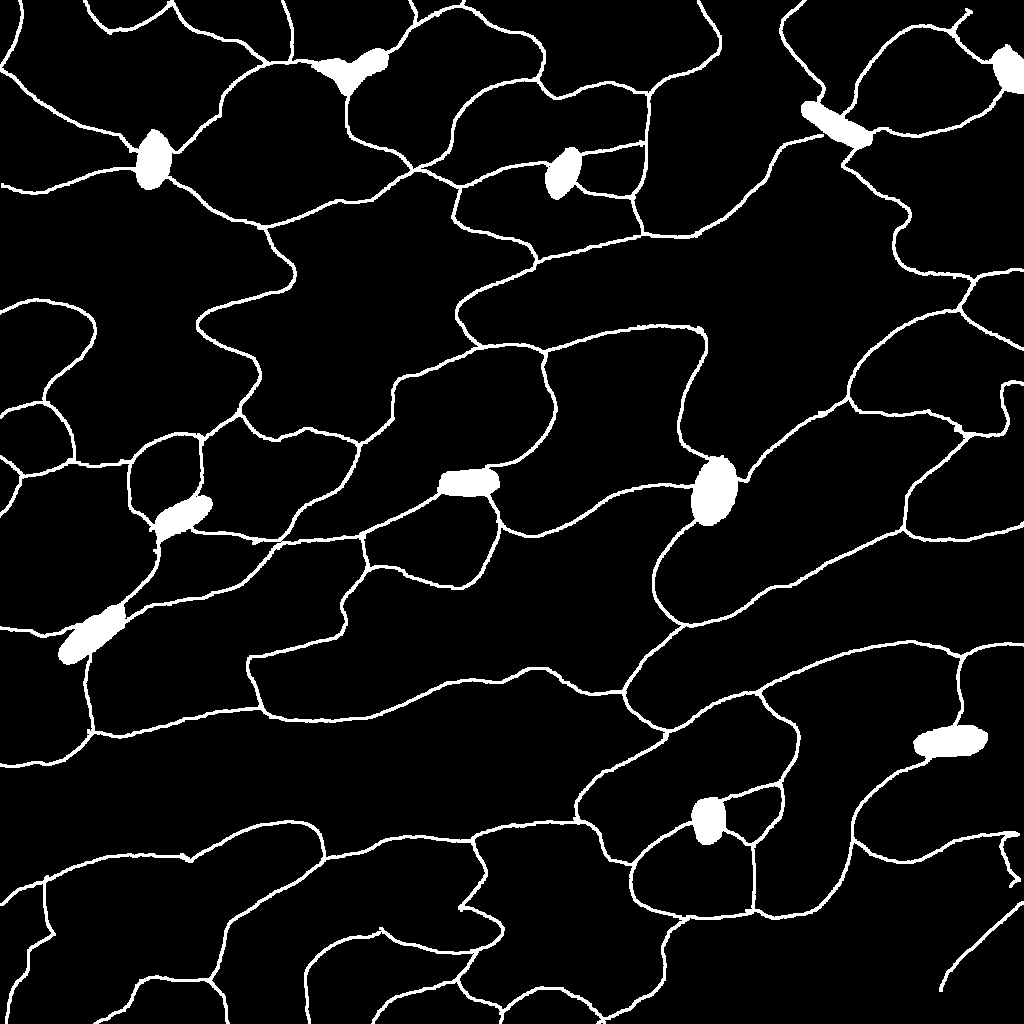

Supplement: Supplementary file 14 — Additional file 14: File S5. Pictures used in this work (raw and analyzed). [file 12915_2022_1495_MOESM14_ESM.xz › Control/C2/2h/mask_checked.tif]

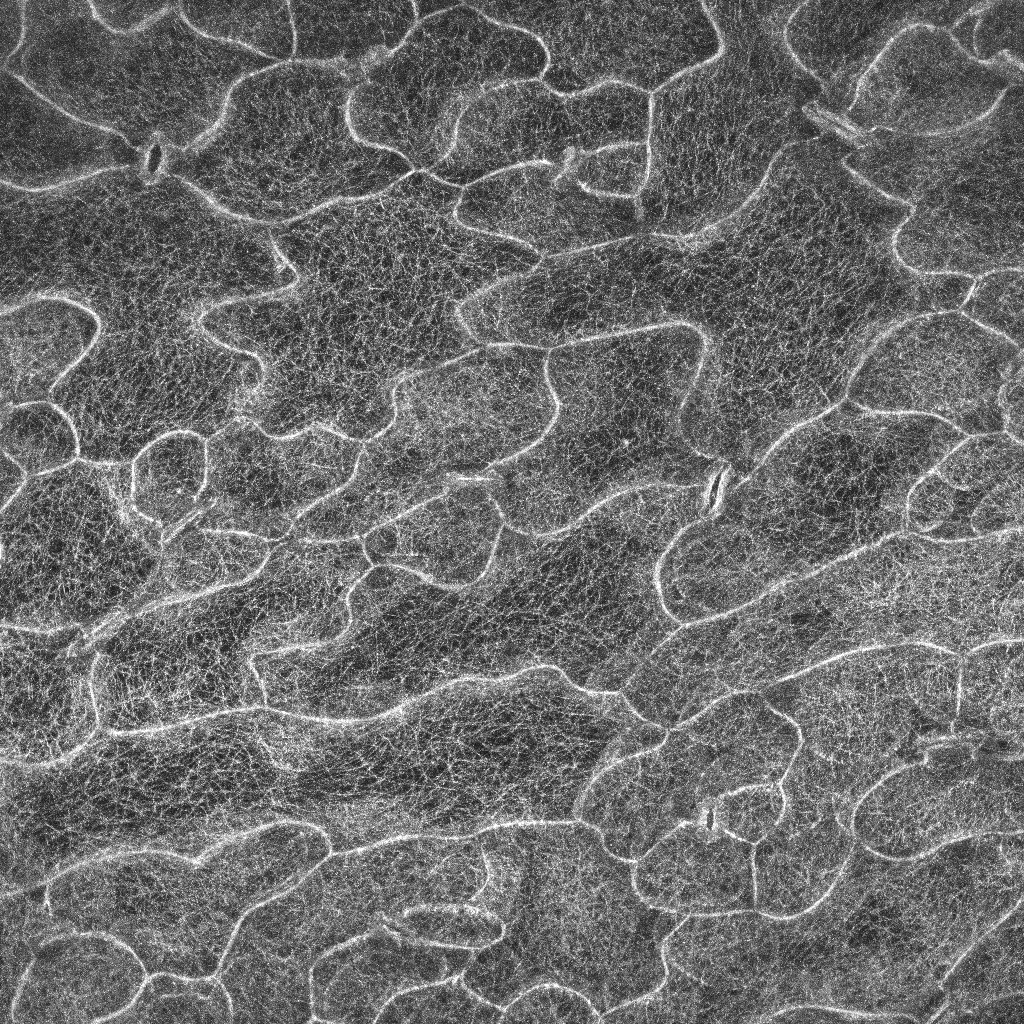

Supplement: Supplementary file 14 — Additional file 14: File S5. Pictures used in this work (raw and analyzed). [file 12915_2022_1495_MOESM14_ESM.xz › Control/C2/KLT_T0_T1/T_0.tif]

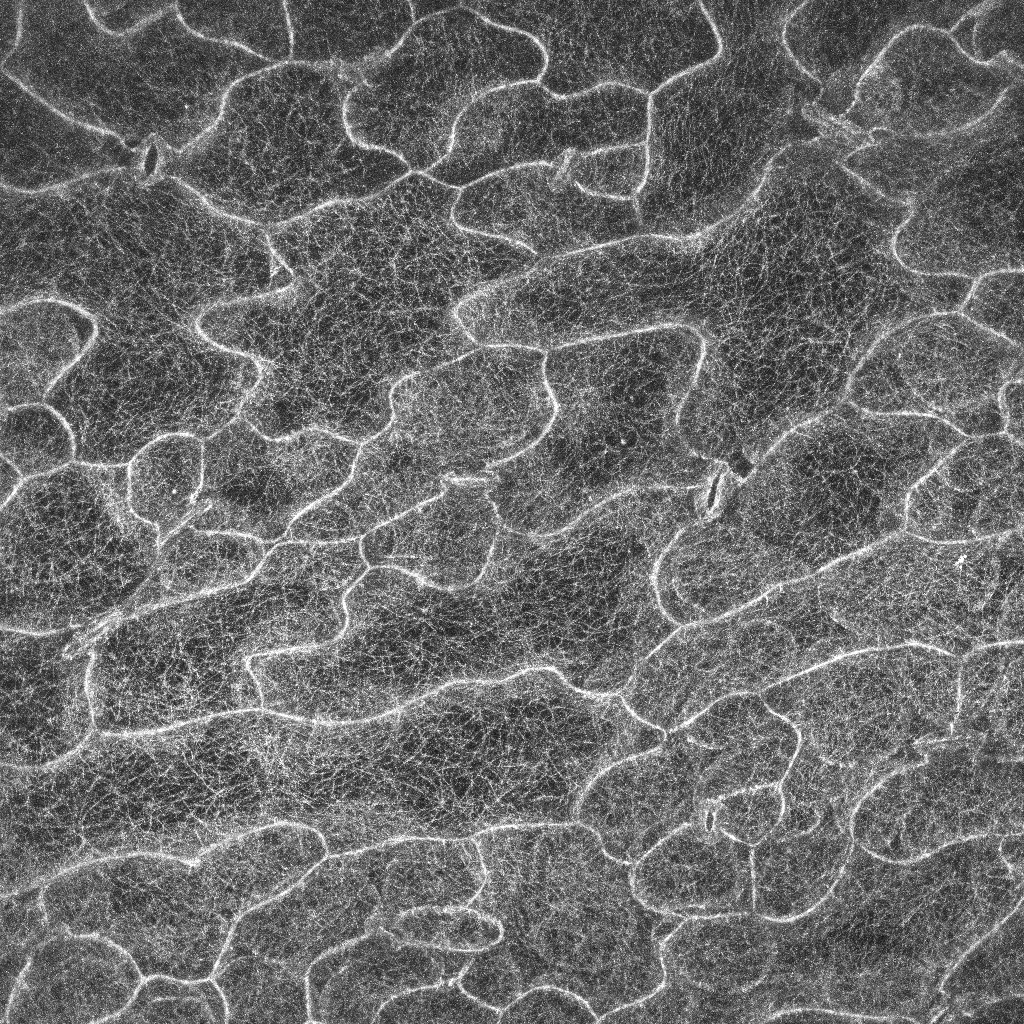

Supplement: Supplementary file 14 — Additional file 14: File S5. Pictures used in this work (raw and analyzed). [file 12915_2022_1495_MOESM14_ESM.xz › Control/C2/KLT_T0_T1/T_1.tif]

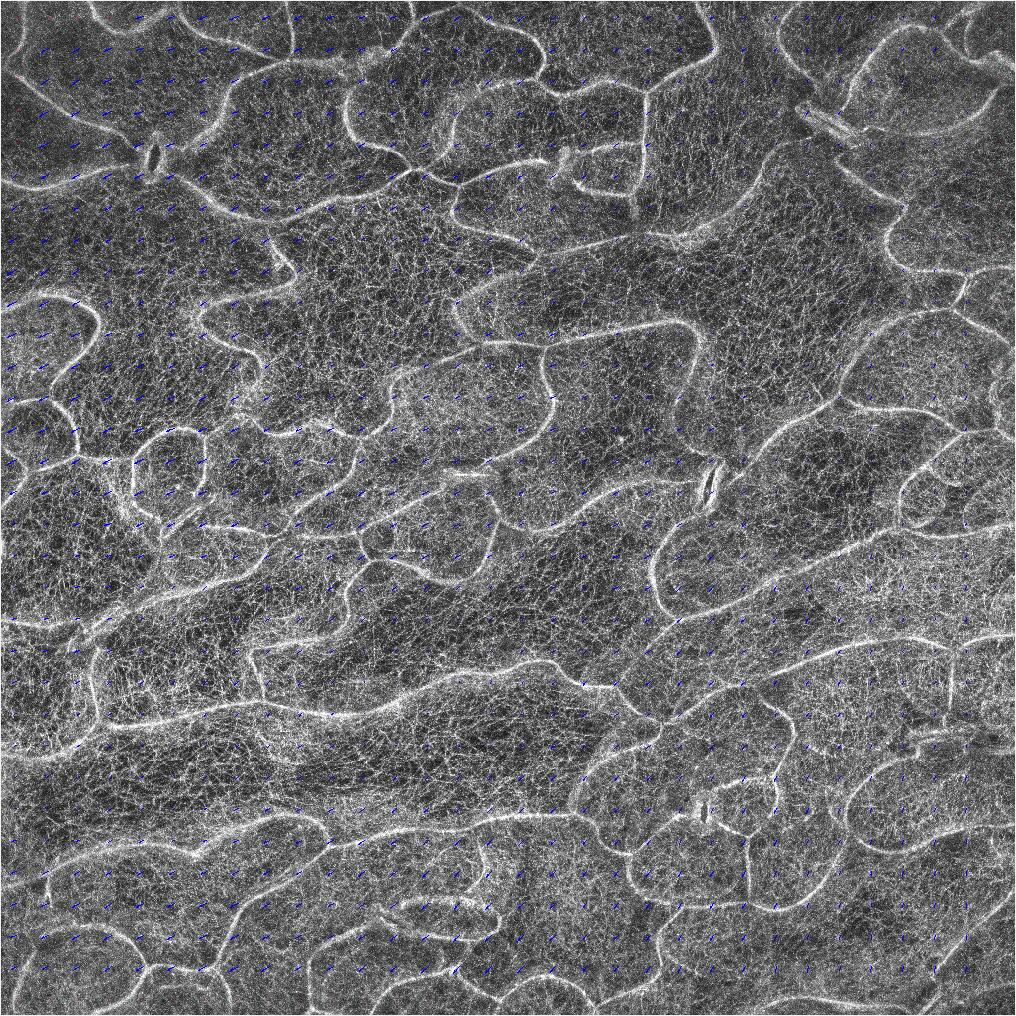

Supplement: Supplementary file 14 — Additional file 14: File S5. Pictures used in this work (raw and analyzed). [file 12915_2022_1495_MOESM14_ESM.xz › Control/C2/KLT_T0_T1/res.jpg]

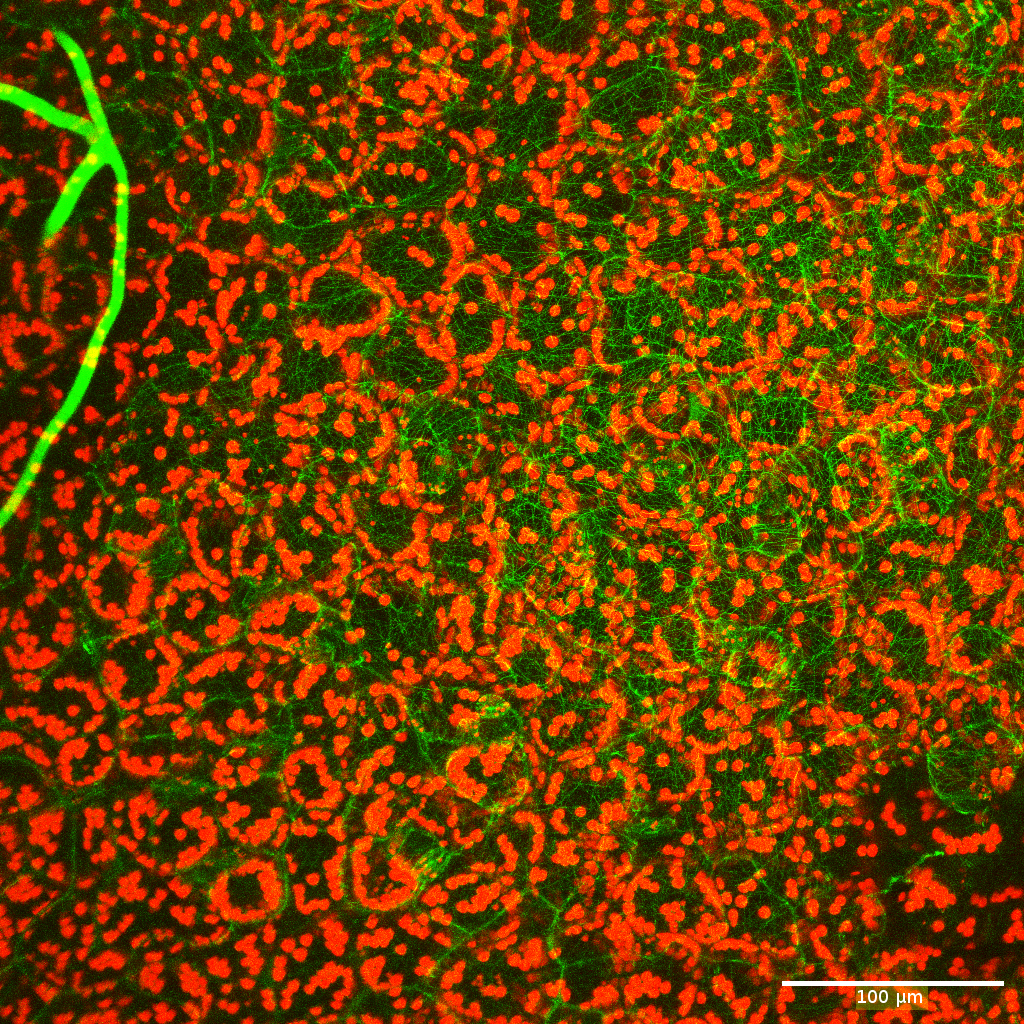

Supplement: Supplementary file 14 — Additional file 14: File S5. Pictures used in this work (raw and analyzed). [file 12915_2022_1495_MOESM14_ESM.xz › Infection/M1/T0.tif]

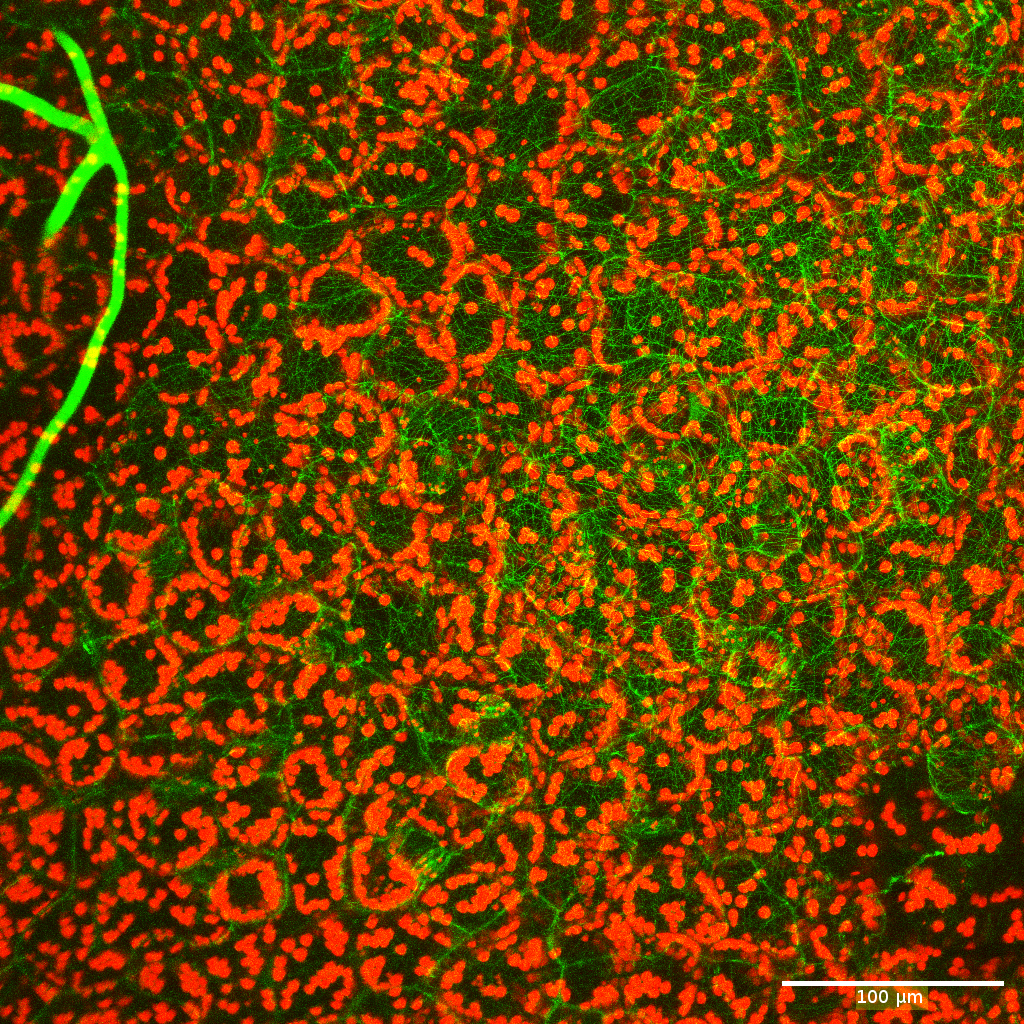

Supplement: Supplementary file 14 — Additional file 14: File S5. Pictures used in this work (raw and analyzed). [file 12915_2022_1495_MOESM14_ESM.xz › Infection/M1/T0.png]

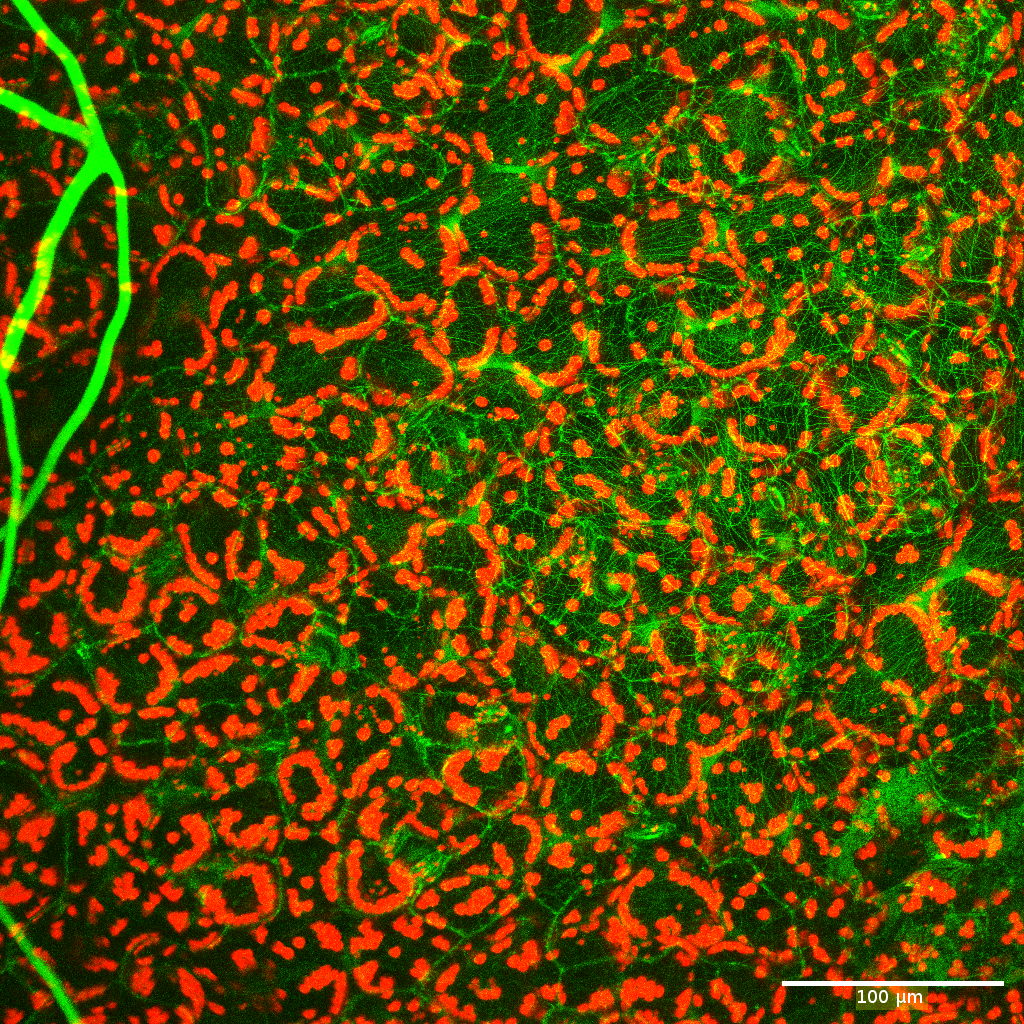

Supplement: Supplementary file 14 — Additional file 14: File S5. Pictures used in this work (raw and analyzed). [file 12915_2022_1495_MOESM14_ESM.xz › Infection/M1/T2.png]

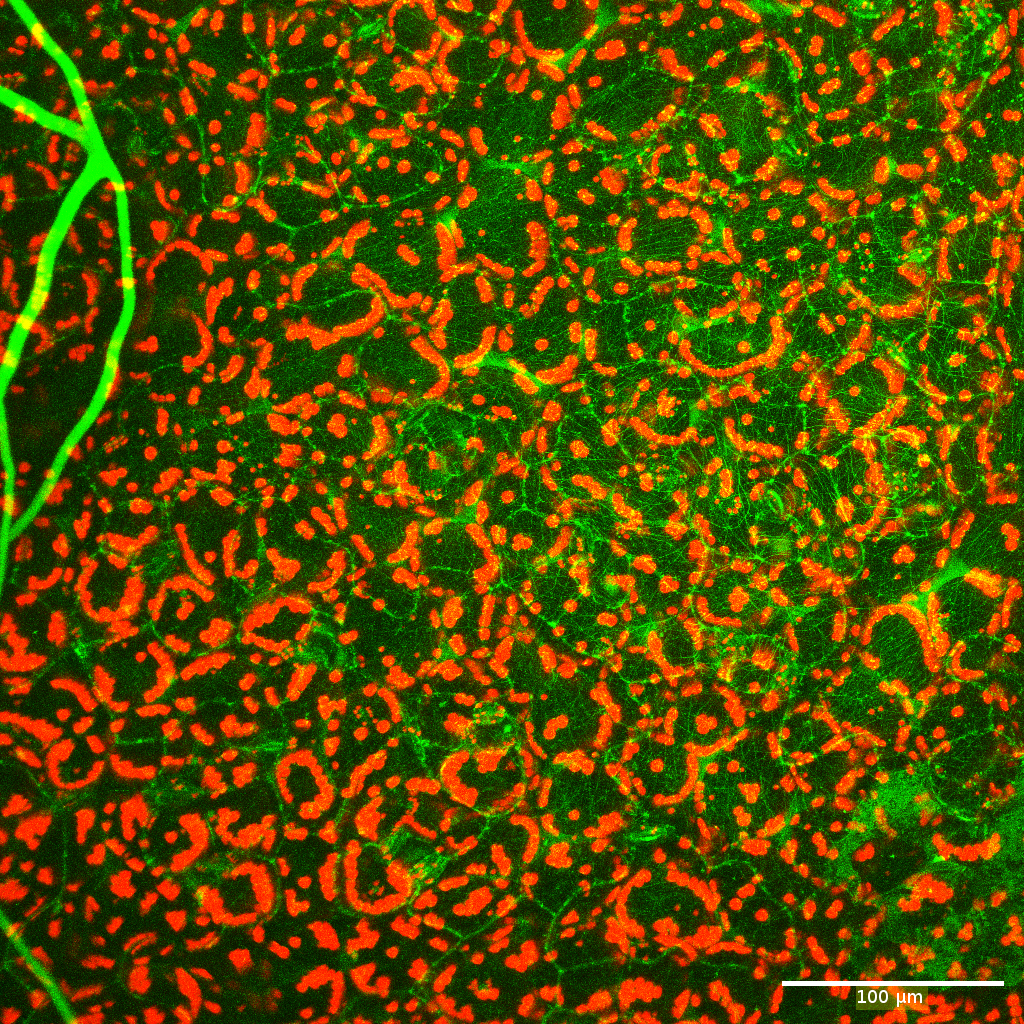

Supplement: Supplementary file 14 — Additional file 14: File S5. Pictures used in this work (raw and analyzed). [file 12915_2022_1495_MOESM14_ESM.xz › Infection/M1/T6.png]

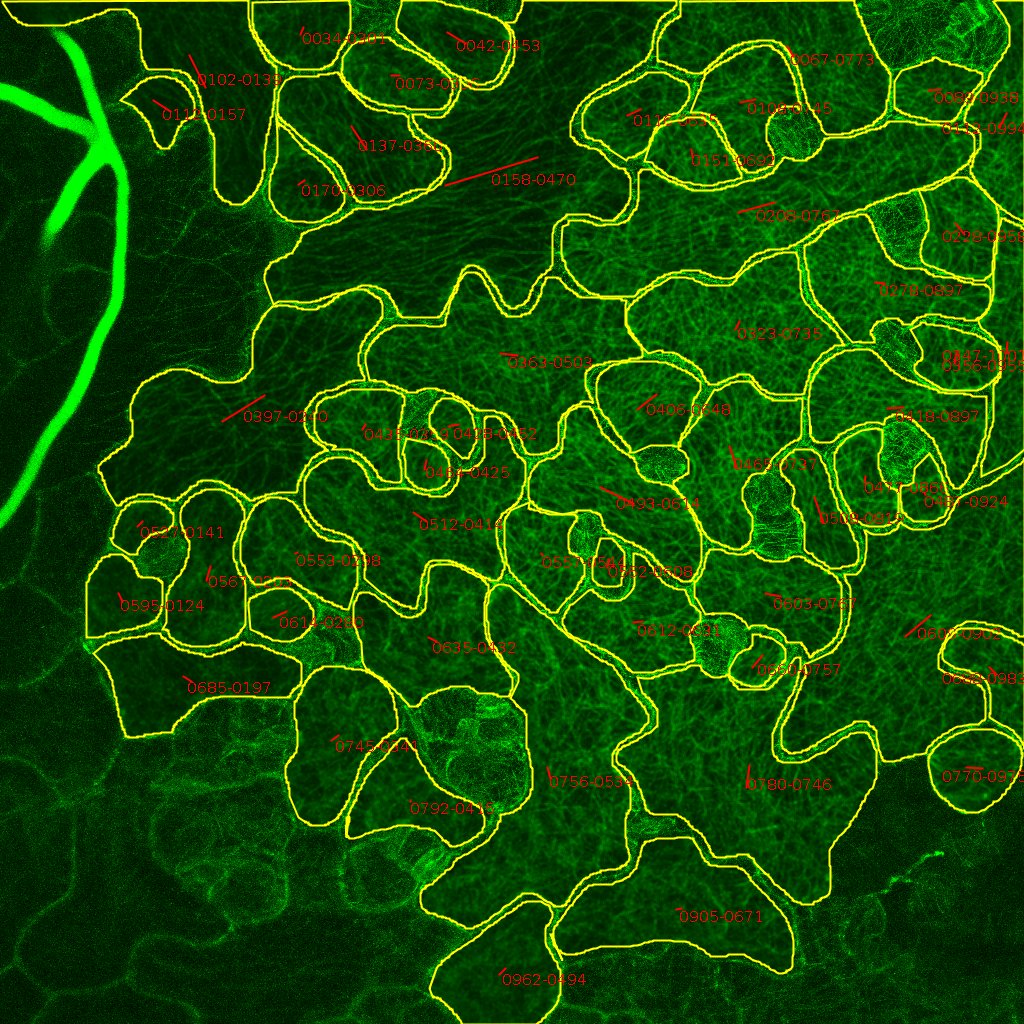

Supplement: Supplementary file 14 — Additional file 14: File S5. Pictures used in this work (raw and analyzed). [file 12915_2022_1495_MOESM14_ESM.xz › Infection/M1/24hpi/T00_fib.jpg]

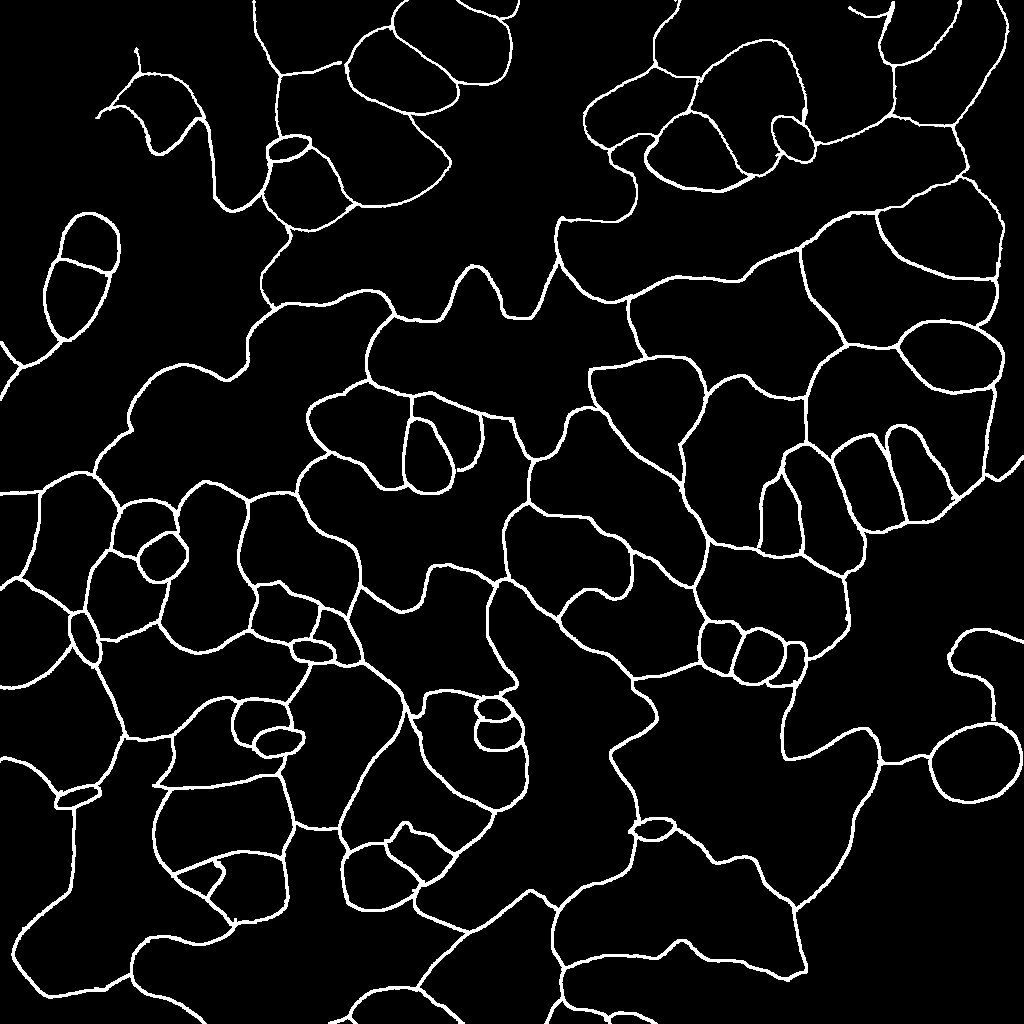

Supplement: Supplementary file 14 — Additional file 14: File S5. Pictures used in this work (raw and analyzed). [file 12915_2022_1495_MOESM14_ESM.xz › Infection/M1/24hpi/mask_checked.tif]

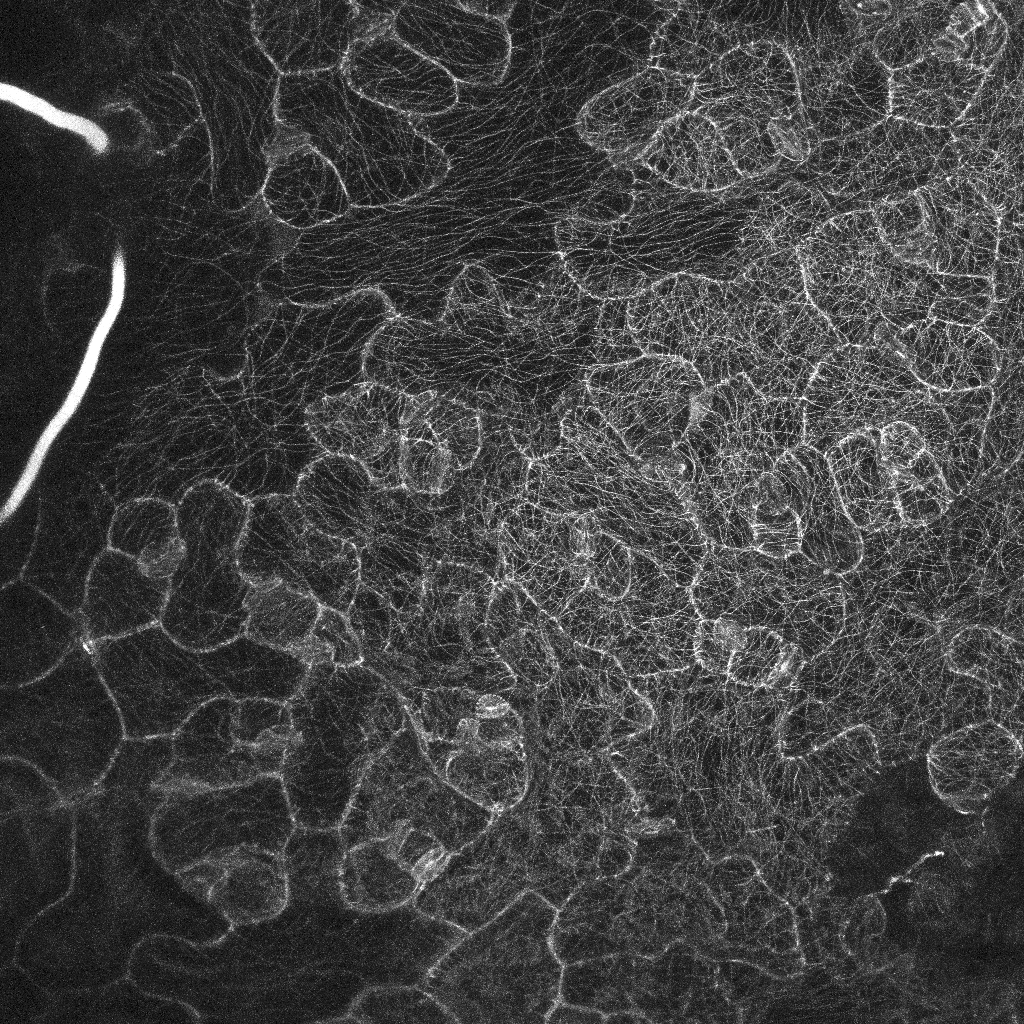

Supplement: Supplementary file 14 — Additional file 14: File S5. Pictures used in this work (raw and analyzed). [file 12915_2022_1495_MOESM14_ESM.xz › Infection/M1/24hpi/grey.tif]

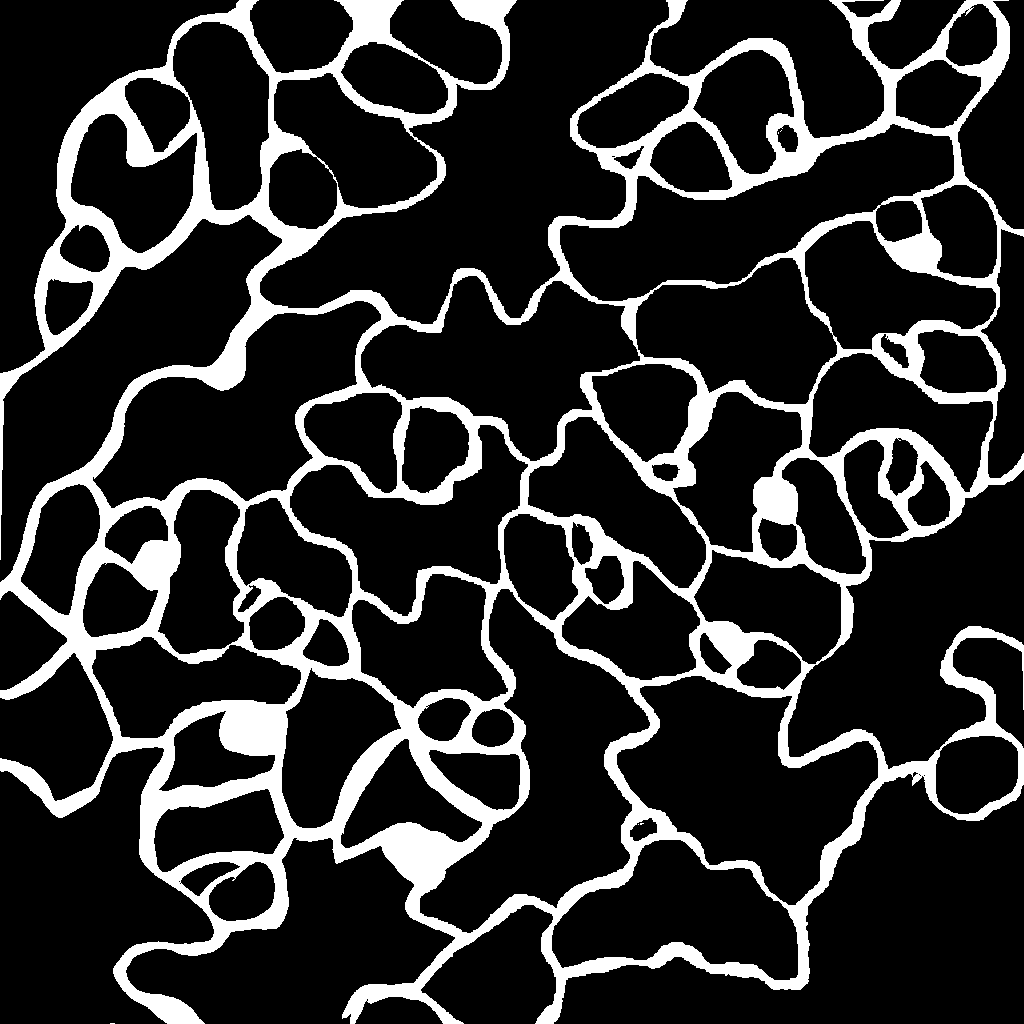

Supplement: Supplementary file 14 — Additional file 14: File S5. Pictures used in this work (raw and analyzed). [file 12915_2022_1495_MOESM14_ESM.xz › Infection/M1/26hpi/mask.tif]

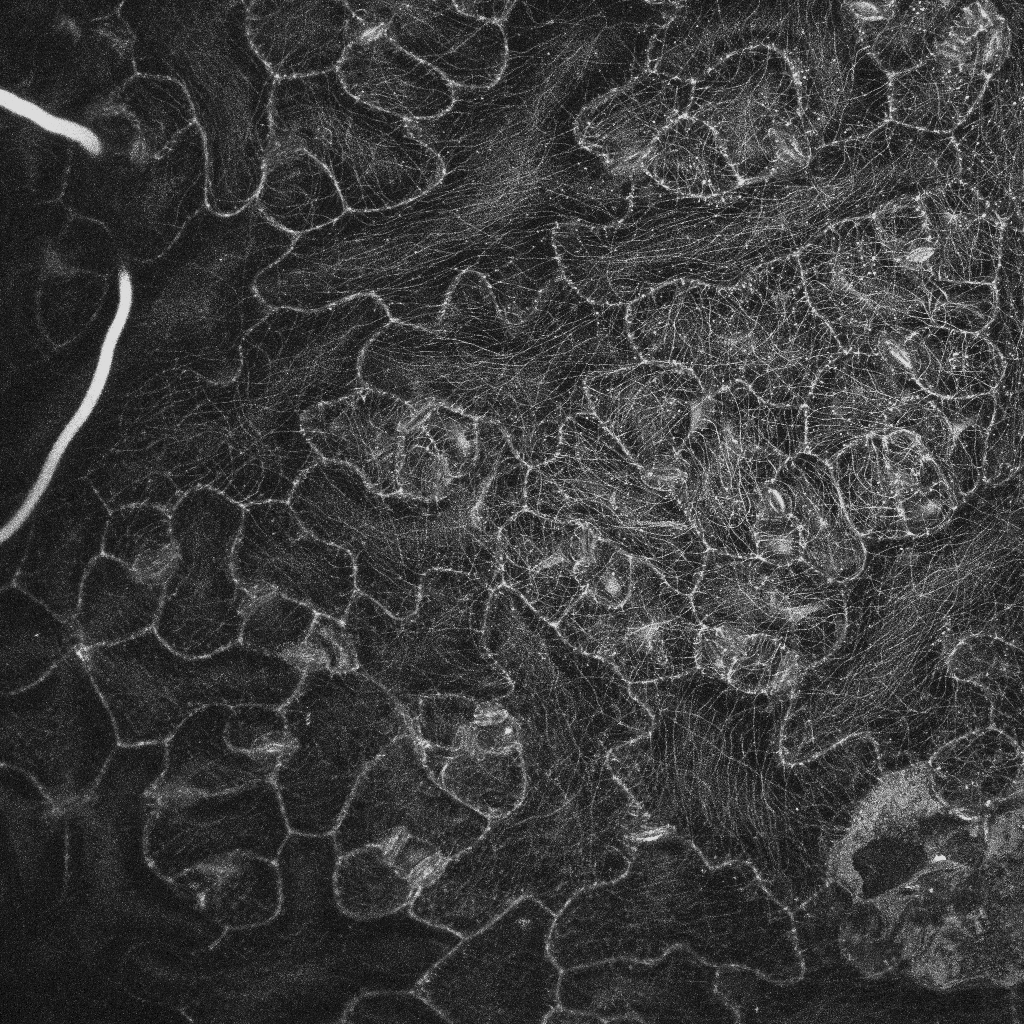

Supplement: Supplementary file 14 — Additional file 14: File S5. Pictures used in this work (raw and analyzed). [file 12915_2022_1495_MOESM14_ESM.xz › Infection/M1/26hpi/grey.tif]

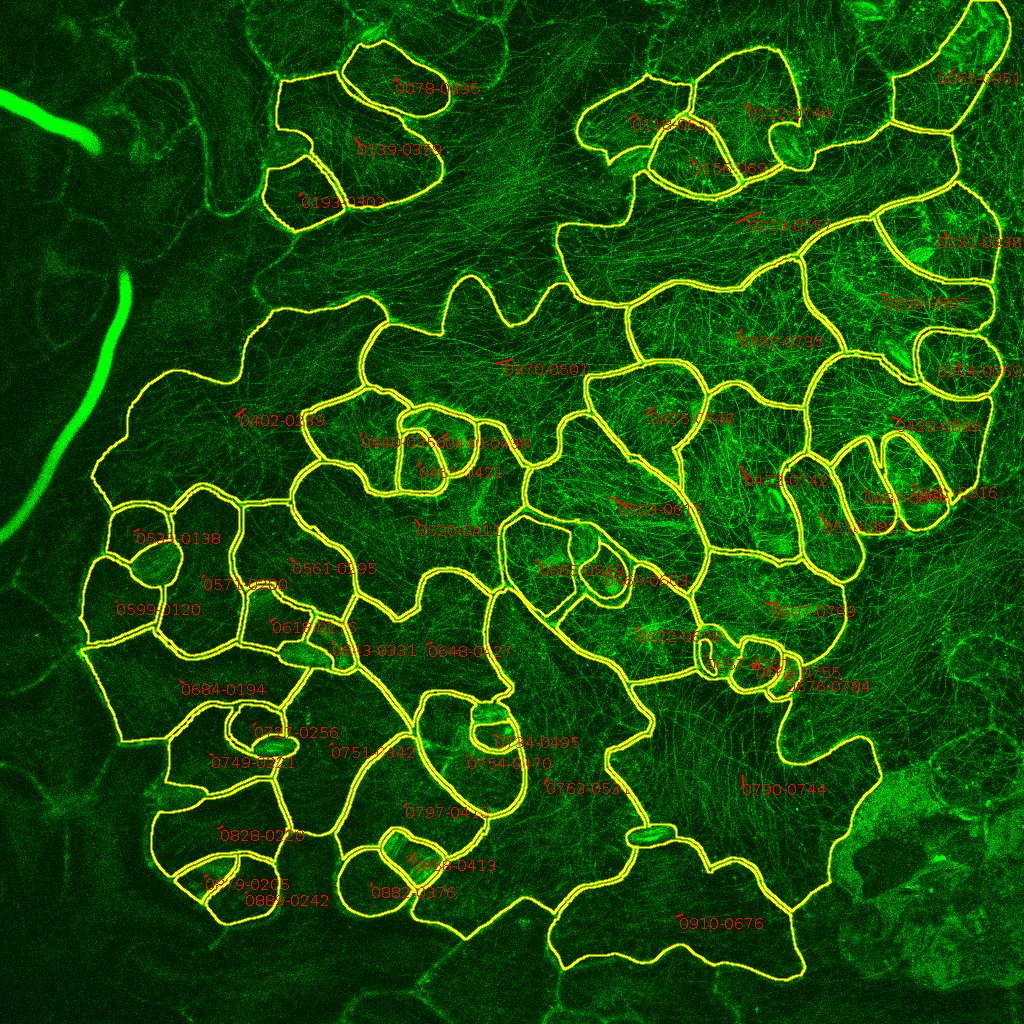

Supplement: Supplementary file 14 — Additional file 14: File S5. Pictures used in this work (raw and analyzed). [file 12915_2022_1495_MOESM14_ESM.xz › Infection/M1/26hpi/T8_fib.jpg]

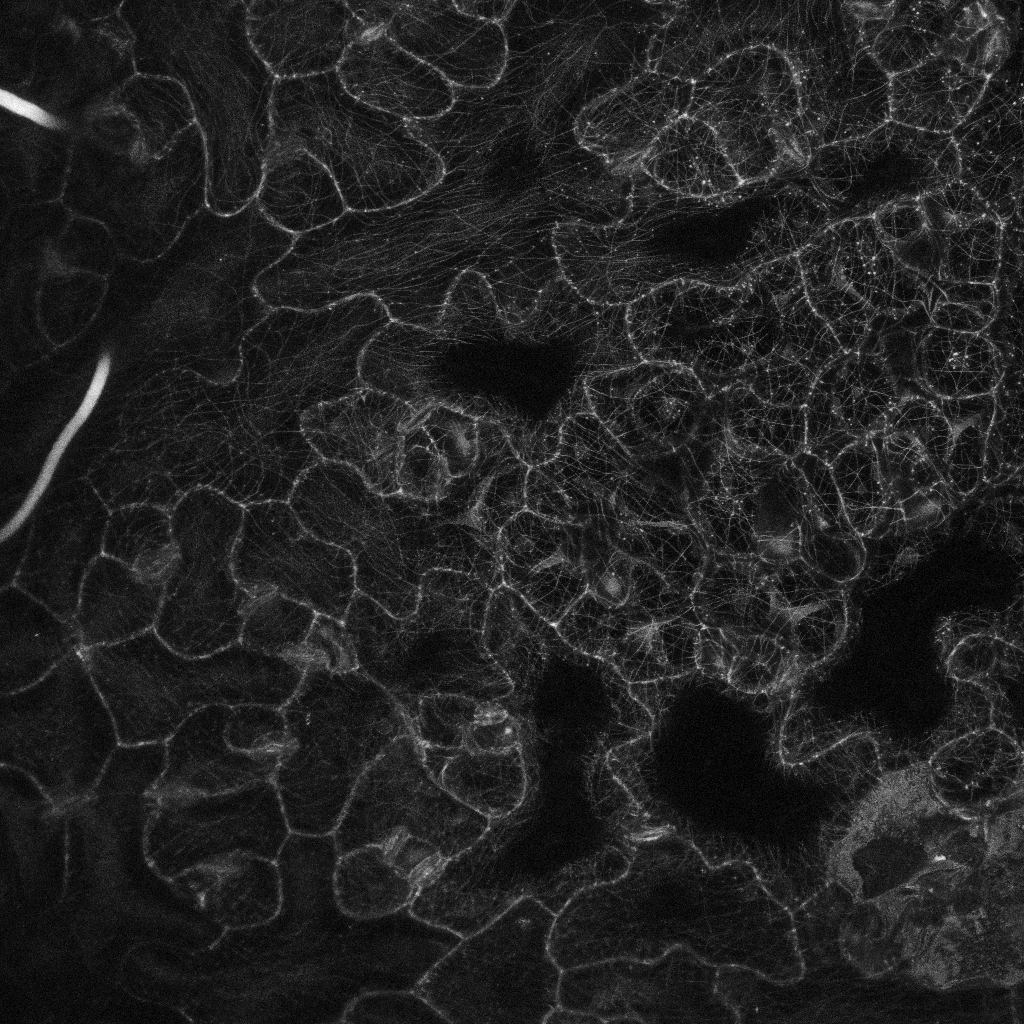

Supplement: Supplementary file 14 — Additional file 14: File S5. Pictures used in this work (raw and analyzed). [file 12915_2022_1495_MOESM14_ESM.xz › Infection/M1/KLT_T0_T2/t_8.tif]

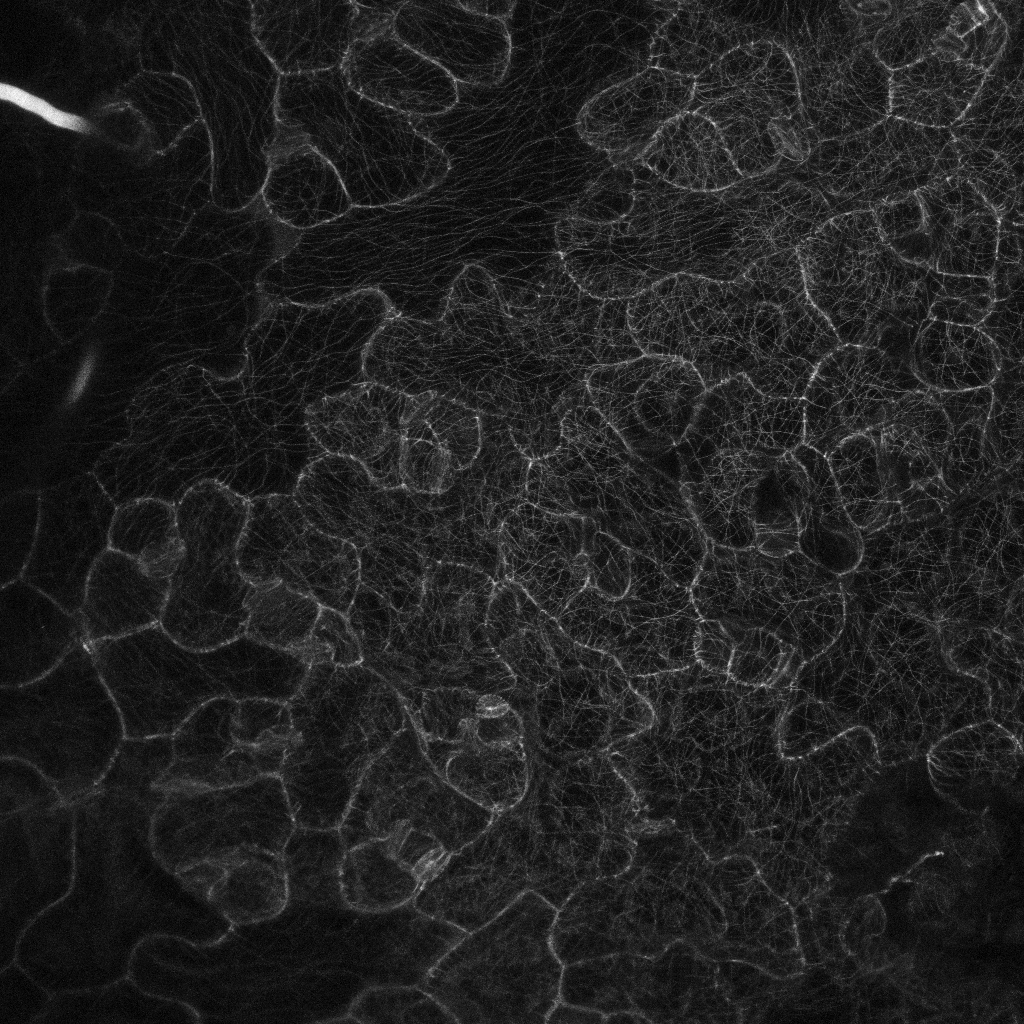

Supplement: Supplementary file 14 — Additional file 14: File S5. Pictures used in this work (raw and analyzed). [file 12915_2022_1495_MOESM14_ESM.xz › Infection/M1/KLT_T0_T2/t_0.tif]

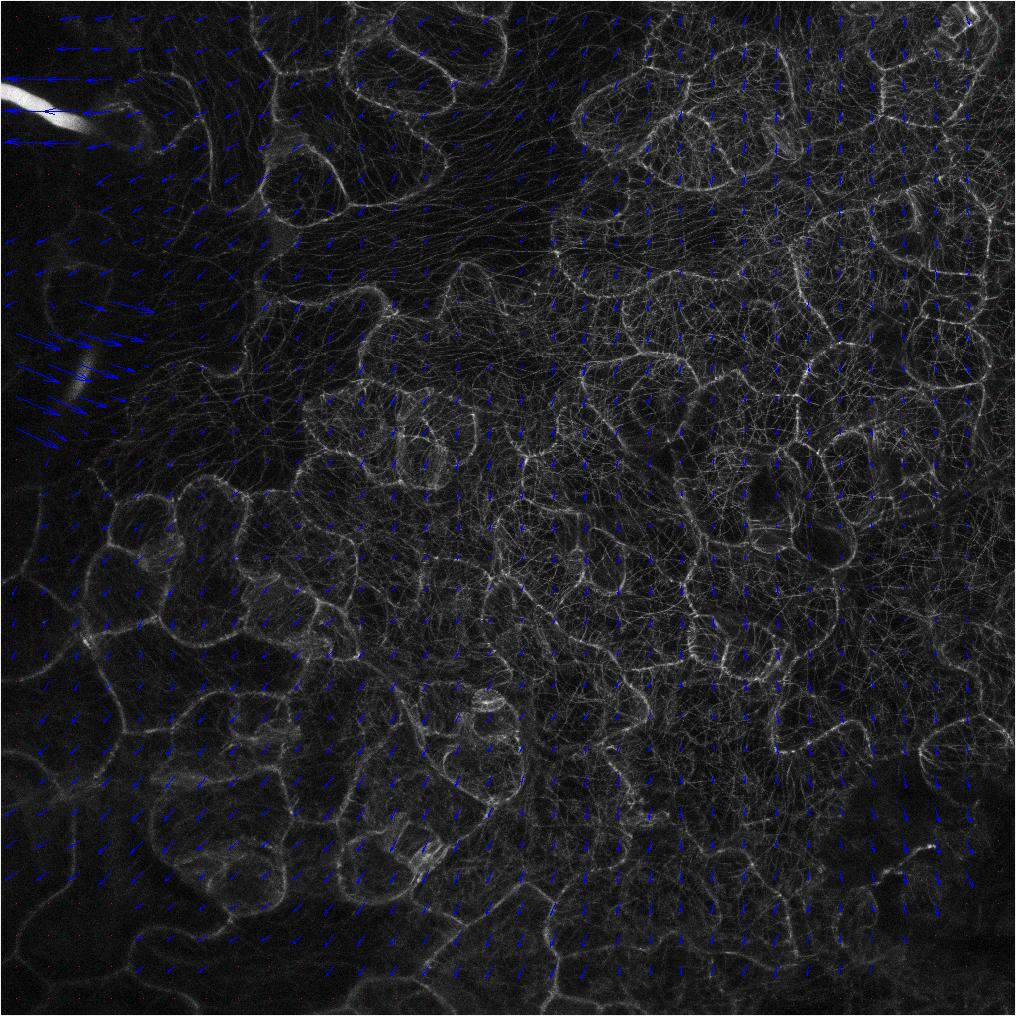

Supplement: Supplementary file 14 — Additional file 14: File S5. Pictures used in this work (raw and analyzed). [file 12915_2022_1495_MOESM14_ESM.xz › Infection/M1/KLT_T0_T2/res.jpg]

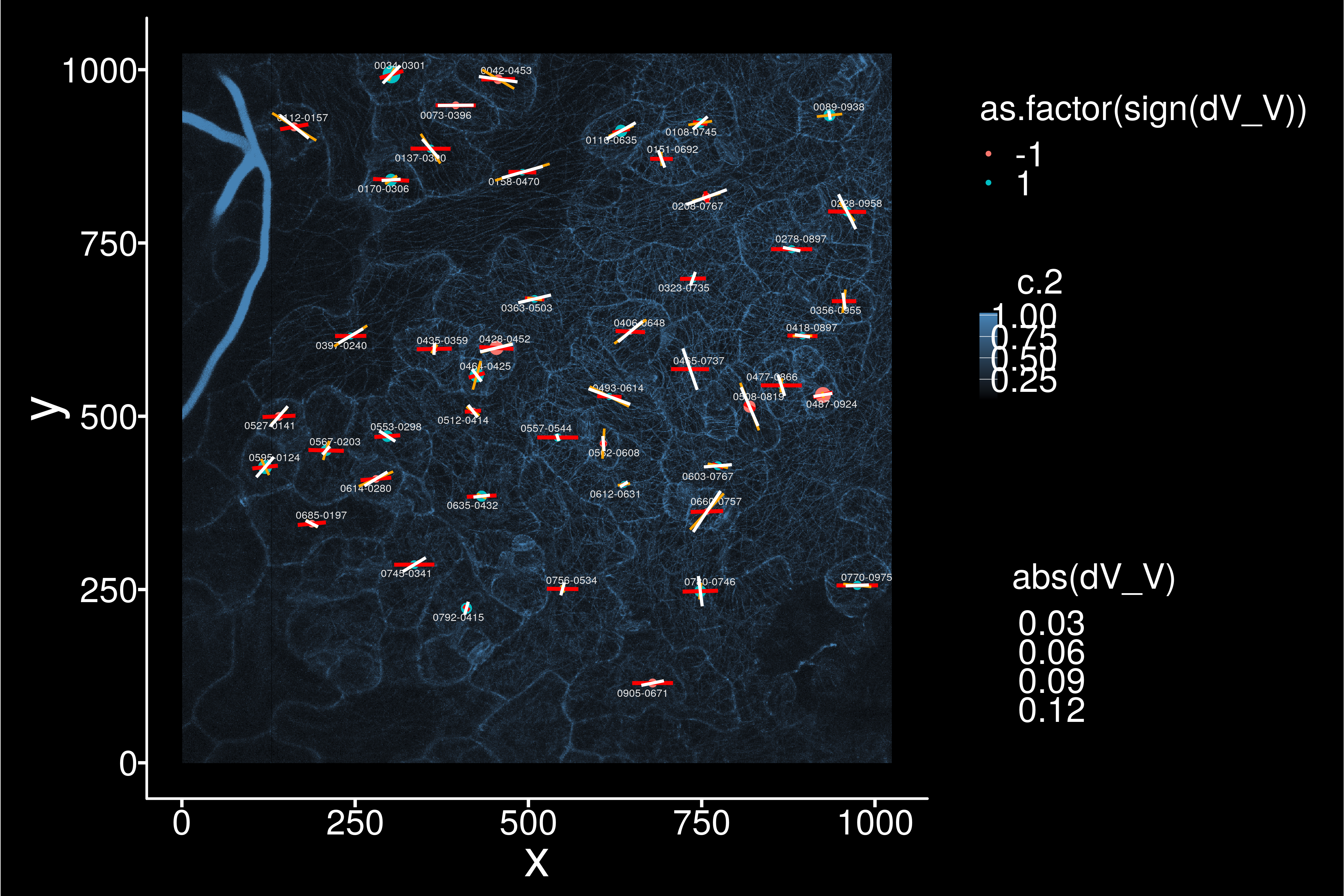

Supplement: Supplementary file 14 — Additional file 14: File S5. Pictures used in this work (raw and analyzed). [file 12915_2022_1495_MOESM14_ESM.xz › Infection/M1/KLT_T0_T2/old/all.tiff]

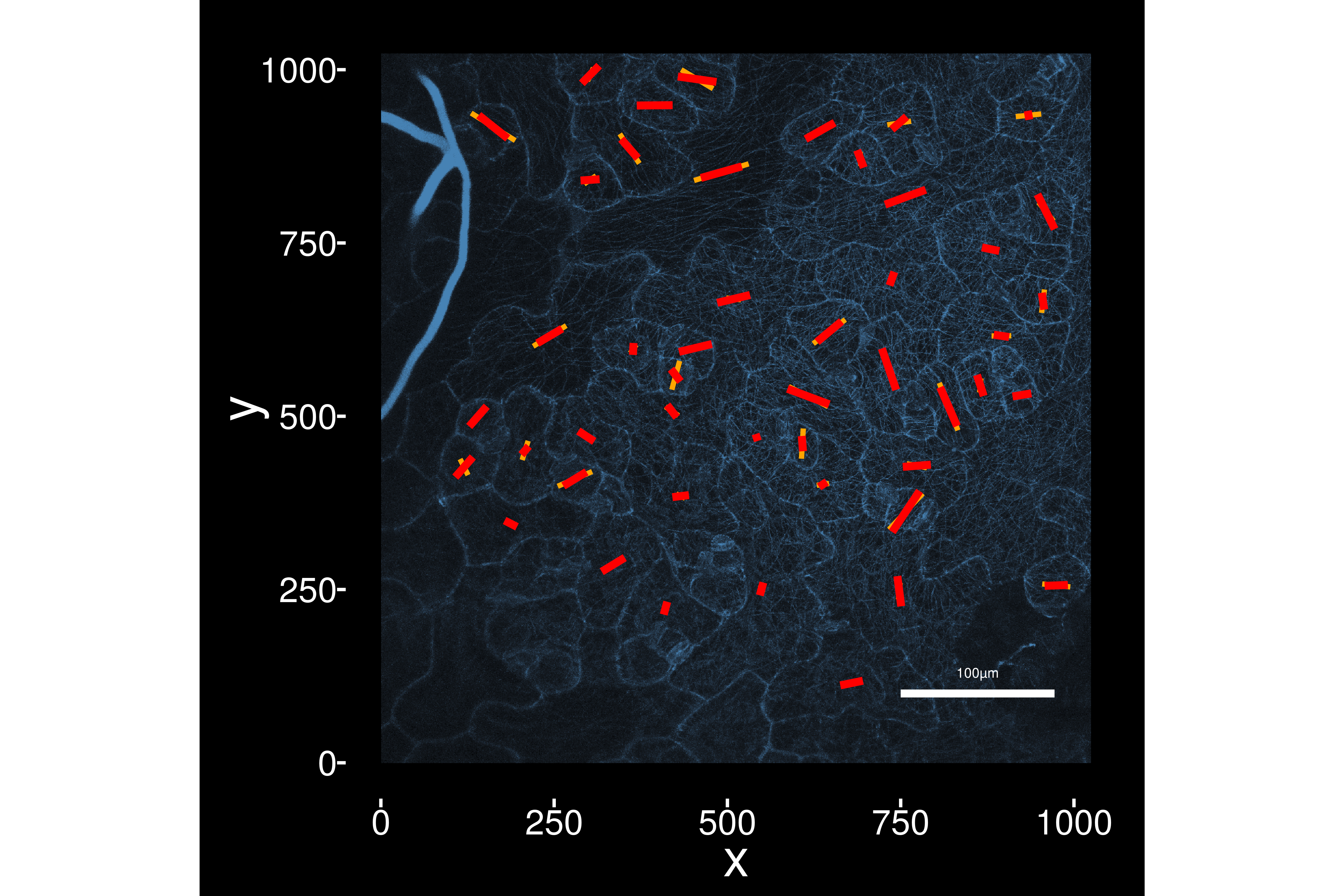

Supplement: Supplementary file 14 — Additional file 14: File S5. Pictures used in this work (raw and analyzed). [file 12915_2022_1495_MOESM14_ESM.xz › Infection/M1/KLT_T0_T2/old/all_ophelie.tiff]

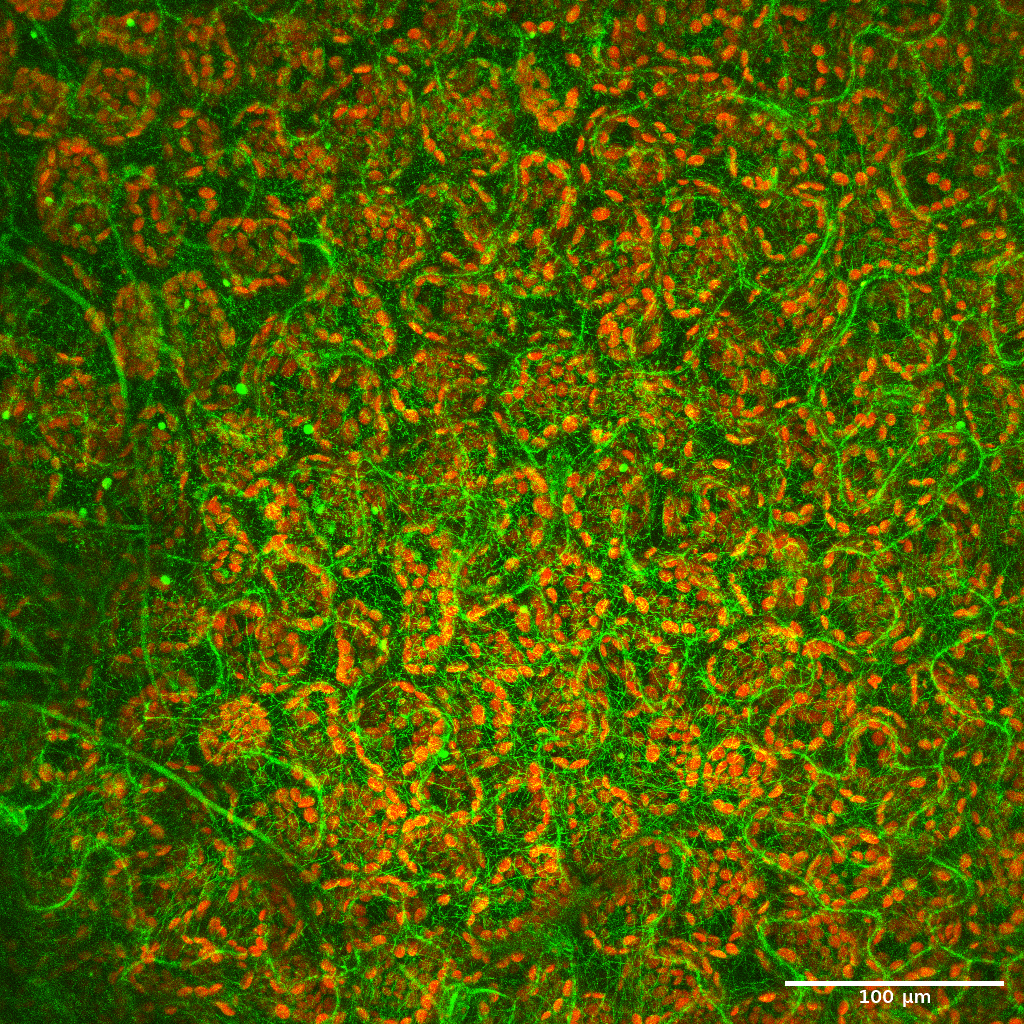

Supplement: Supplementary file 14 — Additional file 14: File S5. Pictures used in this work (raw and analyzed). [file 12915_2022_1495_MOESM14_ESM.xz › Infection/M2/T0.tif]

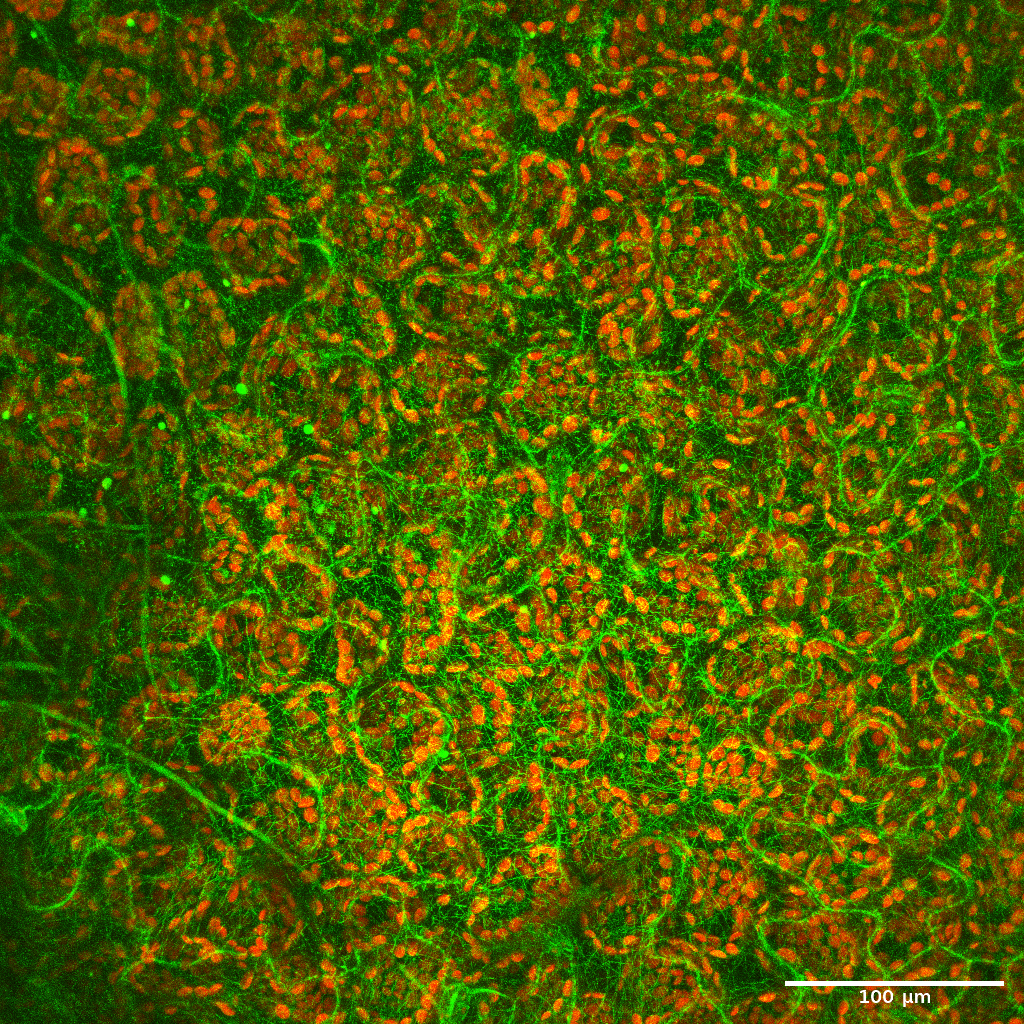

Supplement: Supplementary file 14 — Additional file 14: File S5. Pictures used in this work (raw and analyzed). [file 12915_2022_1495_MOESM14_ESM.xz › Infection/M2/T0.png]

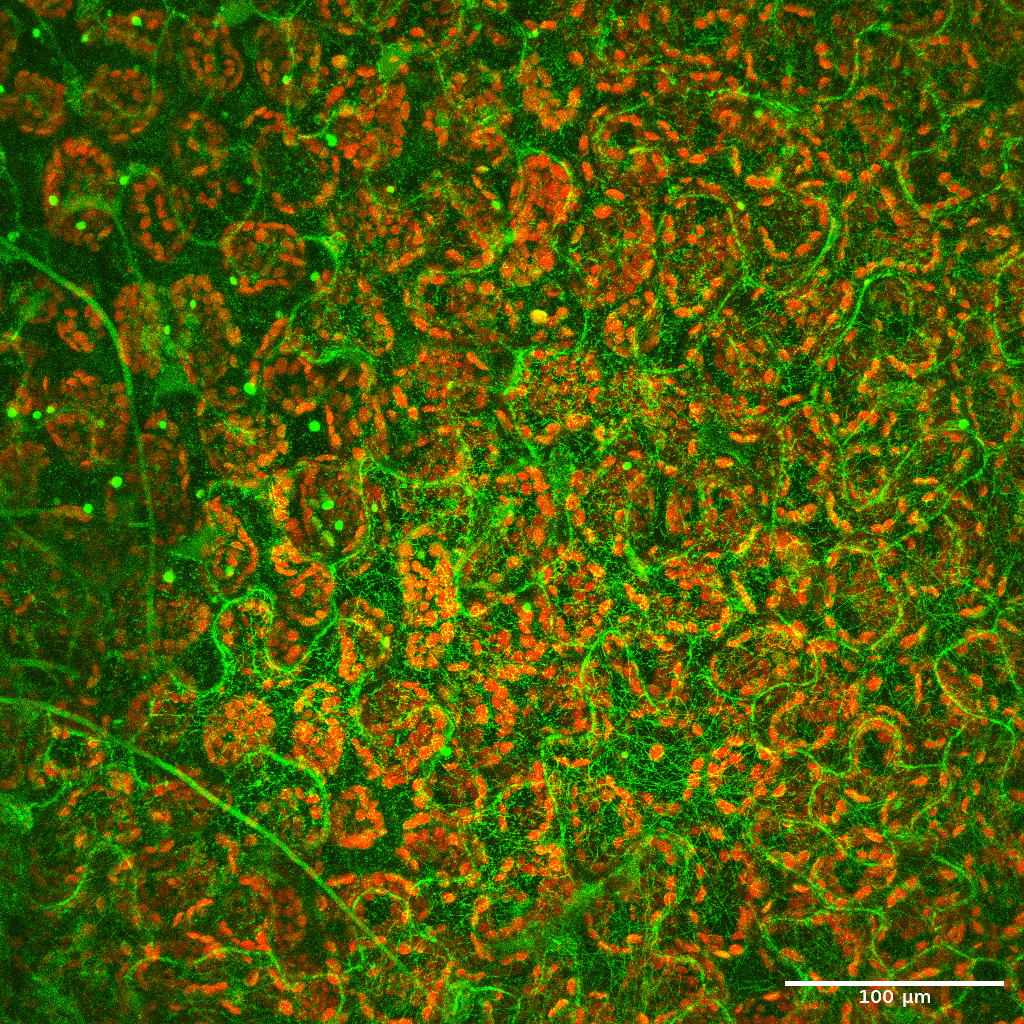

Supplement: Supplementary file 14 — Additional file 14: File S5. Pictures used in this work (raw and analyzed). [file 12915_2022_1495_MOESM14_ESM.xz › Infection/M2/T2.png]

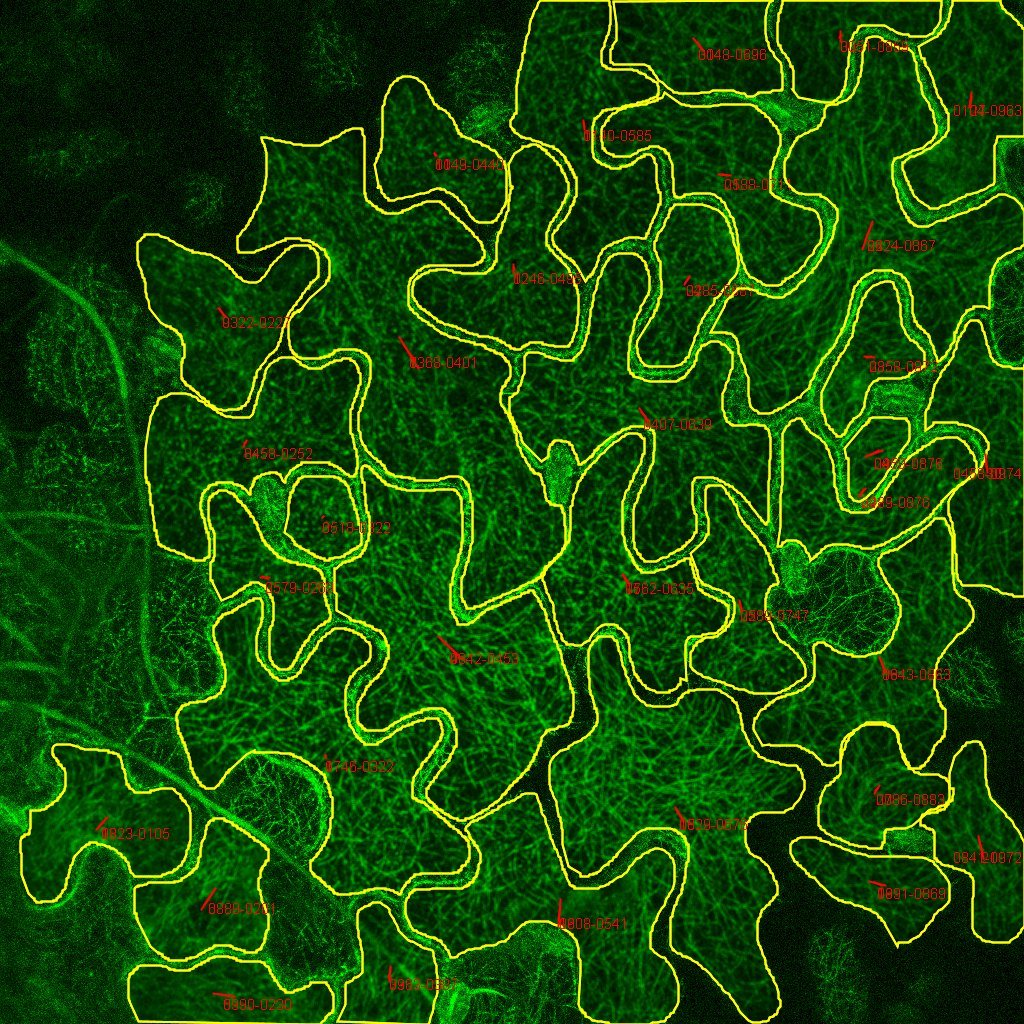

Supplement: Supplementary file 14 — Additional file 14: File S5. Pictures used in this work (raw and analyzed). [file 12915_2022_1495_MOESM14_ESM.xz › Infection/M2/24hpi/T00_fib.jpg]

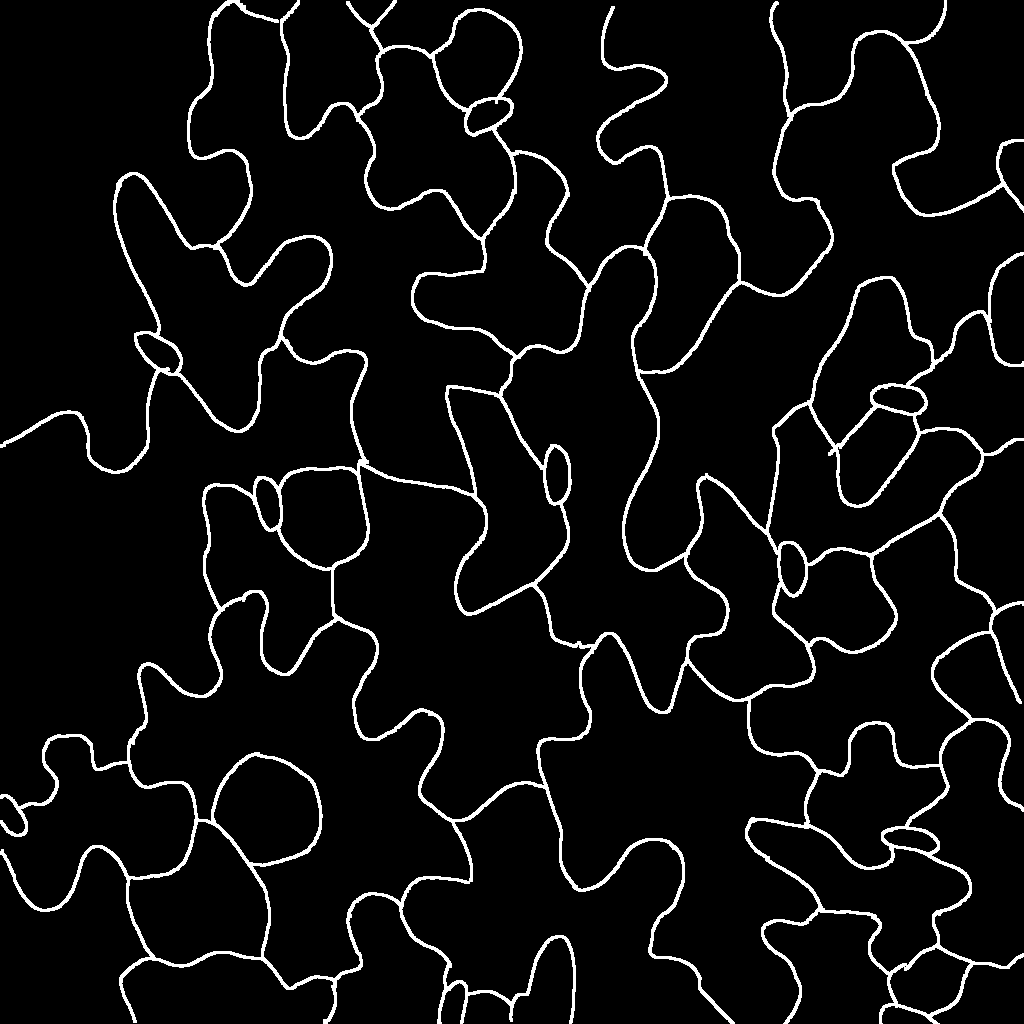

Supplement: Supplementary file 14 — Additional file 14: File S5. Pictures used in this work (raw and analyzed). [file 12915_2022_1495_MOESM14_ESM.xz › Infection/M2/24hpi/mask_checked.tif]

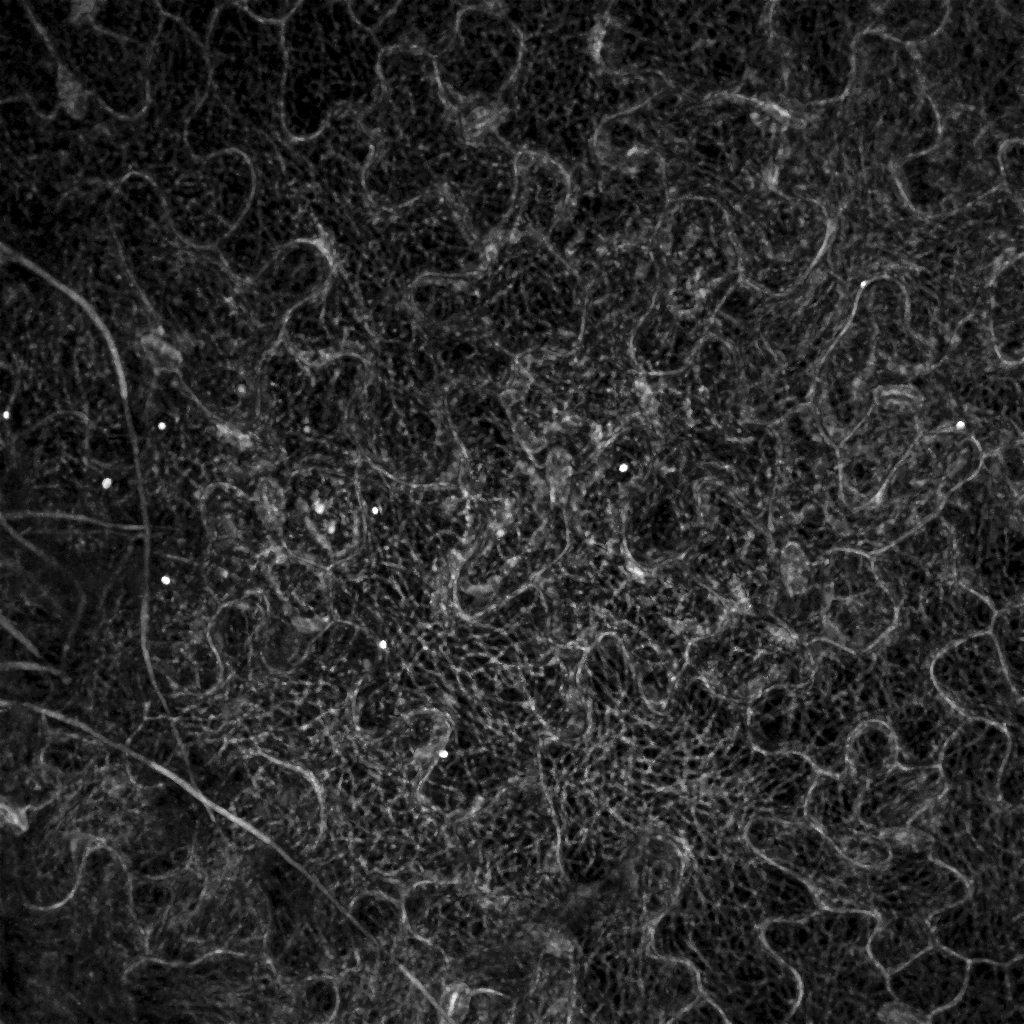

Supplement: Supplementary file 14 — Additional file 14: File S5. Pictures used in this work (raw and analyzed). [file 12915_2022_1495_MOESM14_ESM.xz › Infection/M2/24hpi/grey.tif]

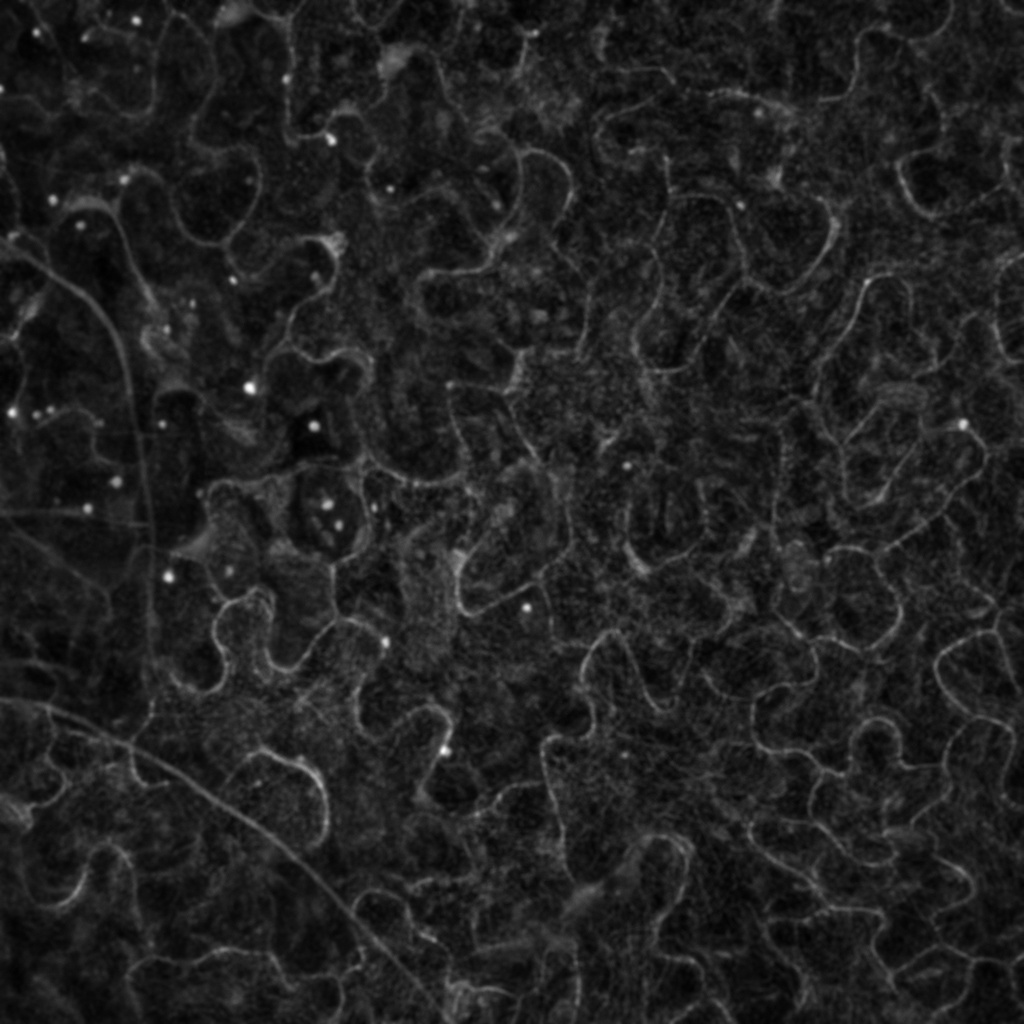

Supplement: Supplementary file 14 — Additional file 14: File S5. Pictures used in this work (raw and analyzed). [file 12915_2022_1495_MOESM14_ESM.xz › Infection/M2/24hpi/KLT_T0_T2/T2.tif]

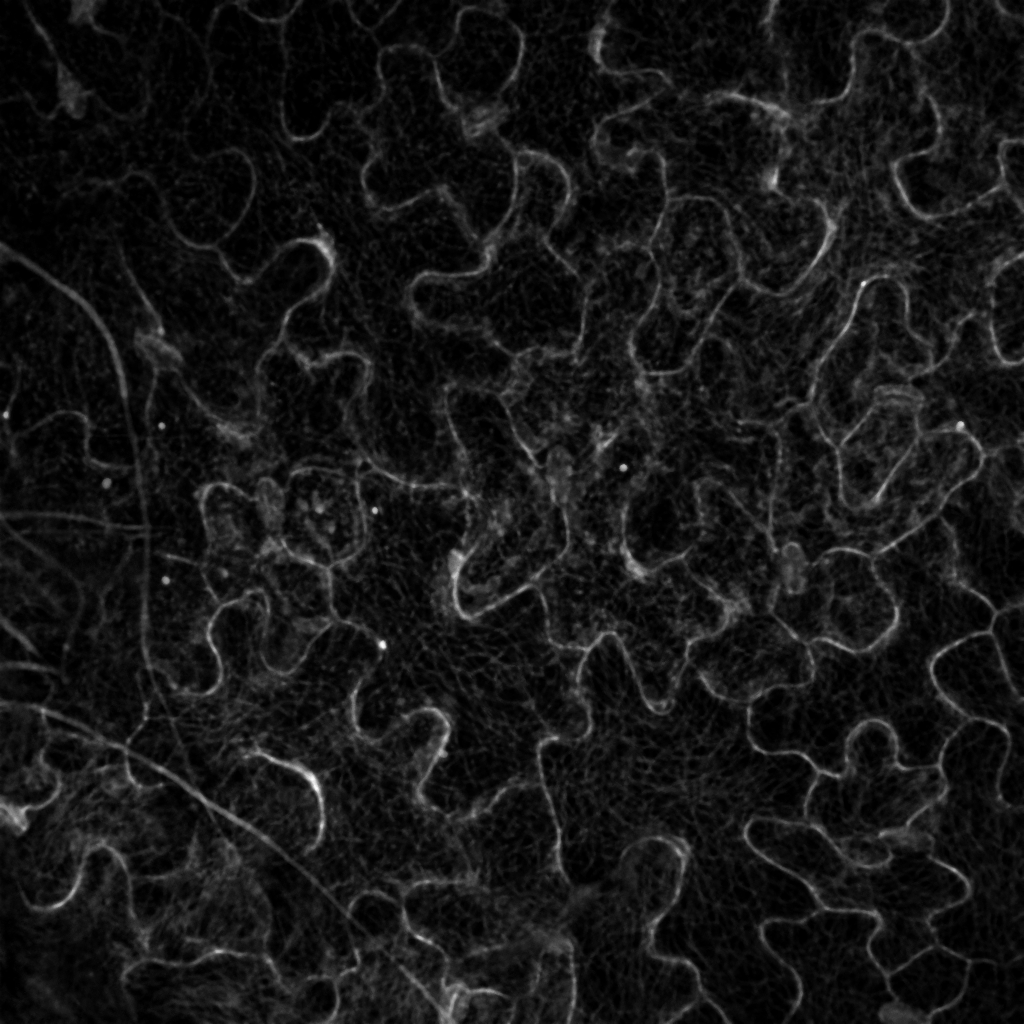

Supplement: Supplementary file 14 — Additional file 14: File S5. Pictures used in this work (raw and analyzed). [file 12915_2022_1495_MOESM14_ESM.xz › Infection/M2/24hpi/KLT_T0_T2/T0.tif]

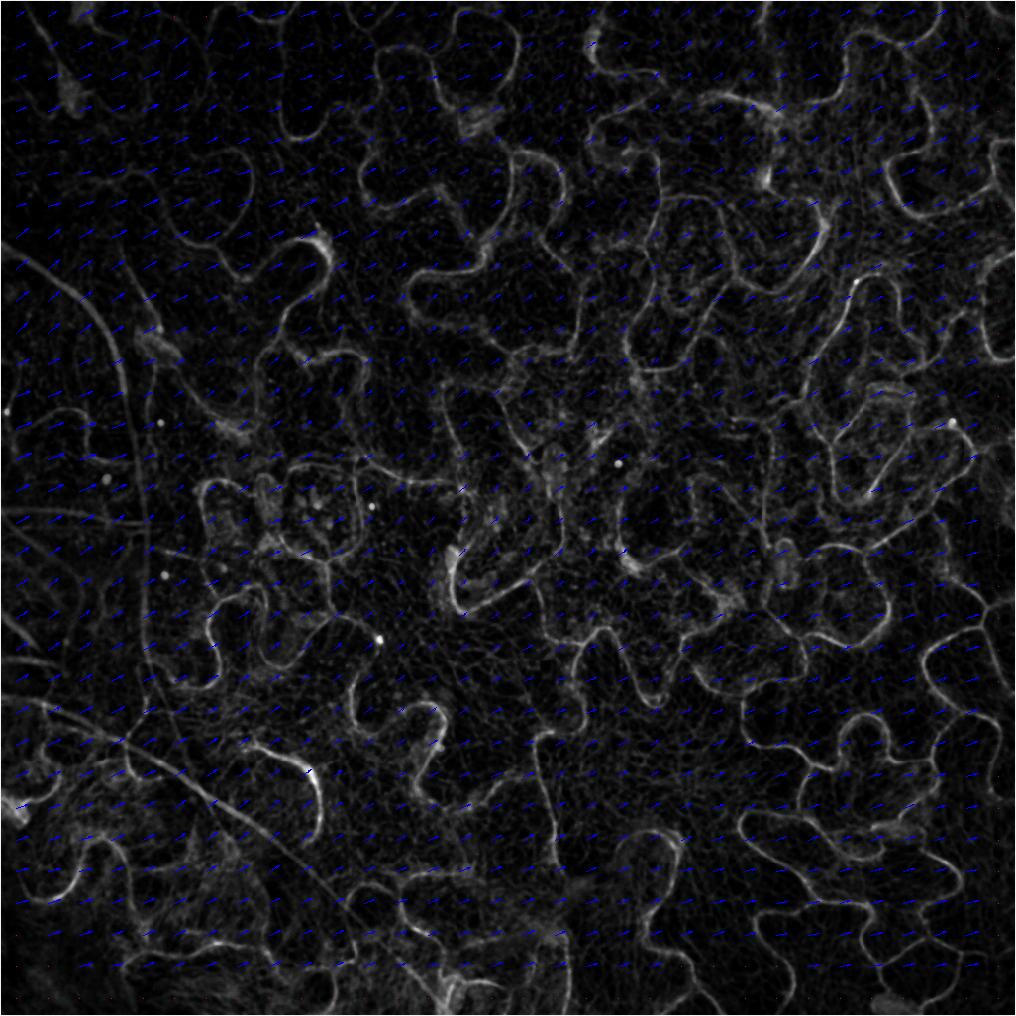

Supplement: Supplementary file 14 — Additional file 14: File S5. Pictures used in this work (raw and analyzed). [file 12915_2022_1495_MOESM14_ESM.xz › Infection/M2/24hpi/KLT_T0_T2/res.jpg]

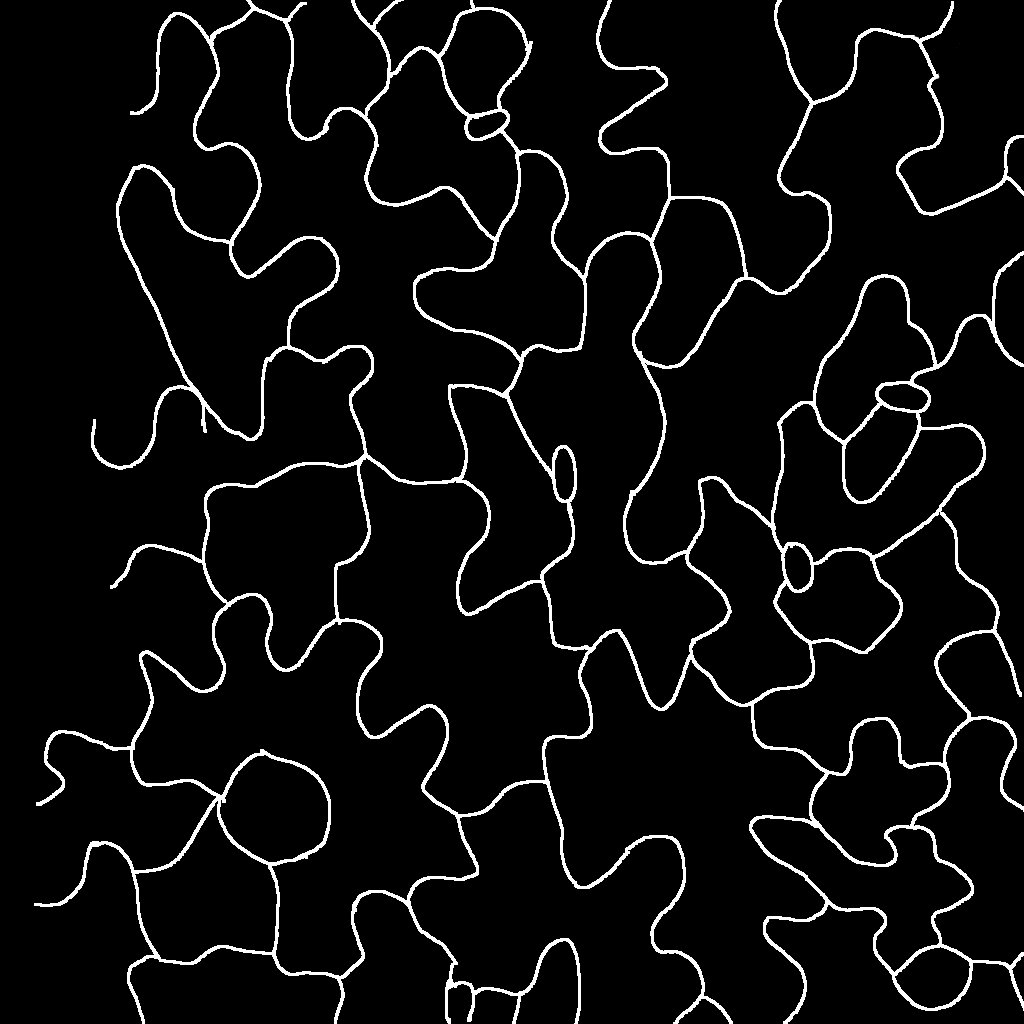

Supplement: Supplementary file 14 — Additional file 14: File S5. Pictures used in this work (raw and analyzed). [file 12915_2022_1495_MOESM14_ESM.xz › Infection/M2/26hpi/mask_checked.tif]

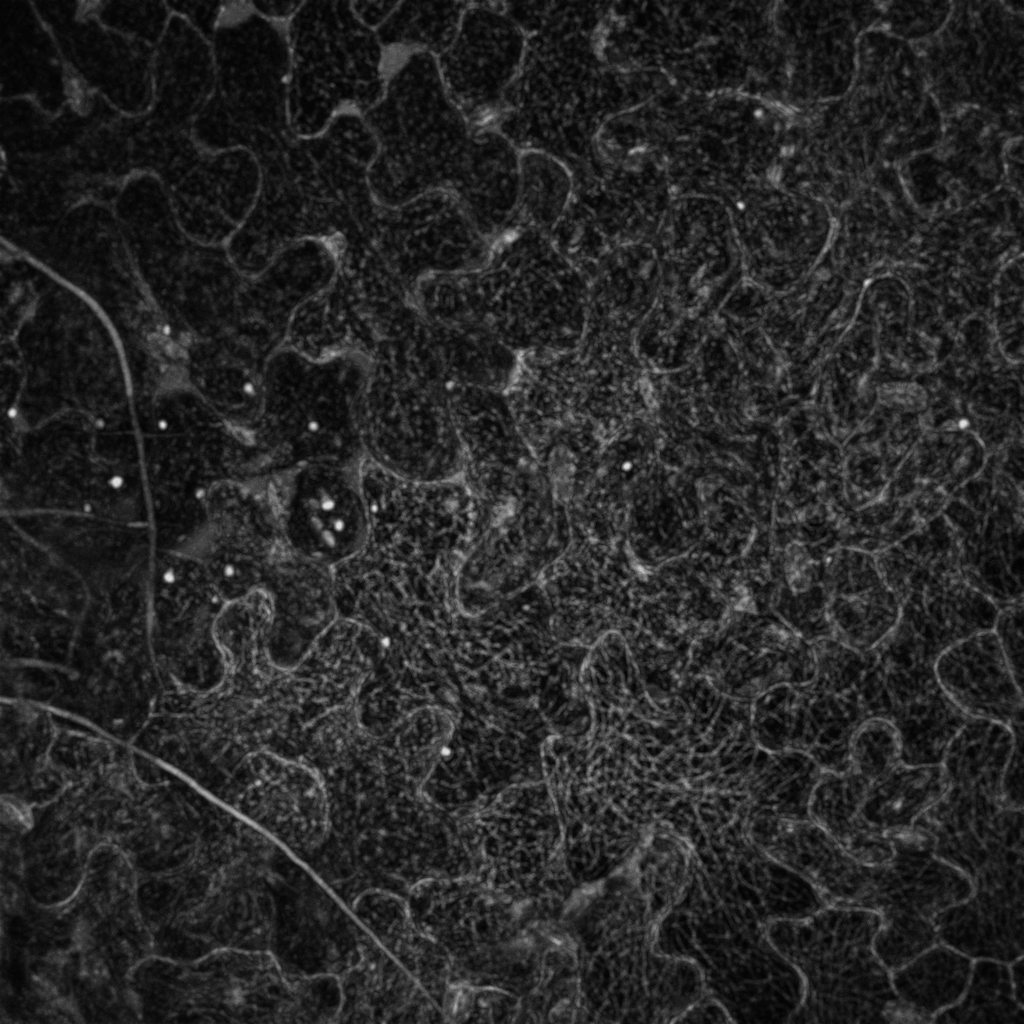

Supplement: Supplementary file 14 — Additional file 14: File S5. Pictures used in this work (raw and analyzed). [file 12915_2022_1495_MOESM14_ESM.xz › Infection/M2/26hpi/grey.tif]

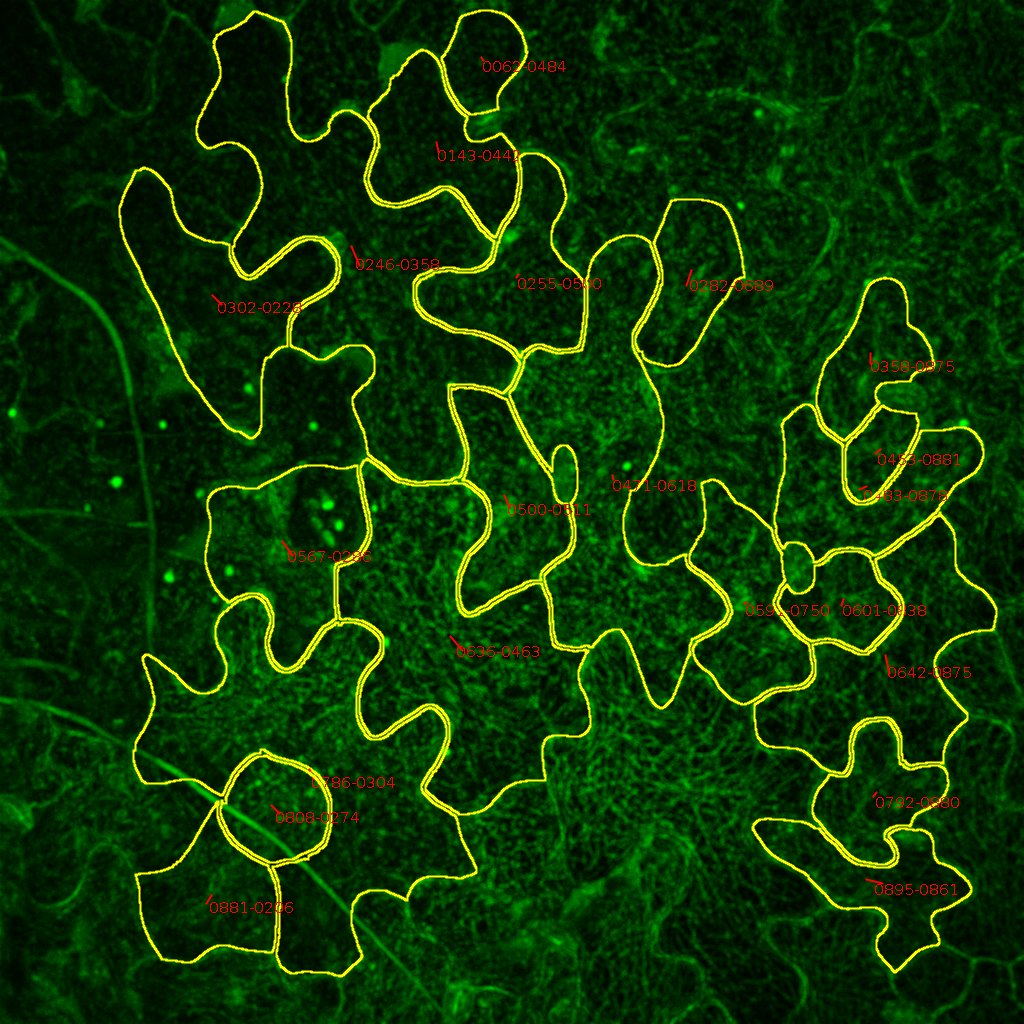

Supplement: Supplementary file 14 — Additional file 14: File S5. Pictures used in this work (raw and analyzed). [file 12915_2022_1495_MOESM14_ESM.xz › Infection/M2/26hpi/T2_fib.jpg]

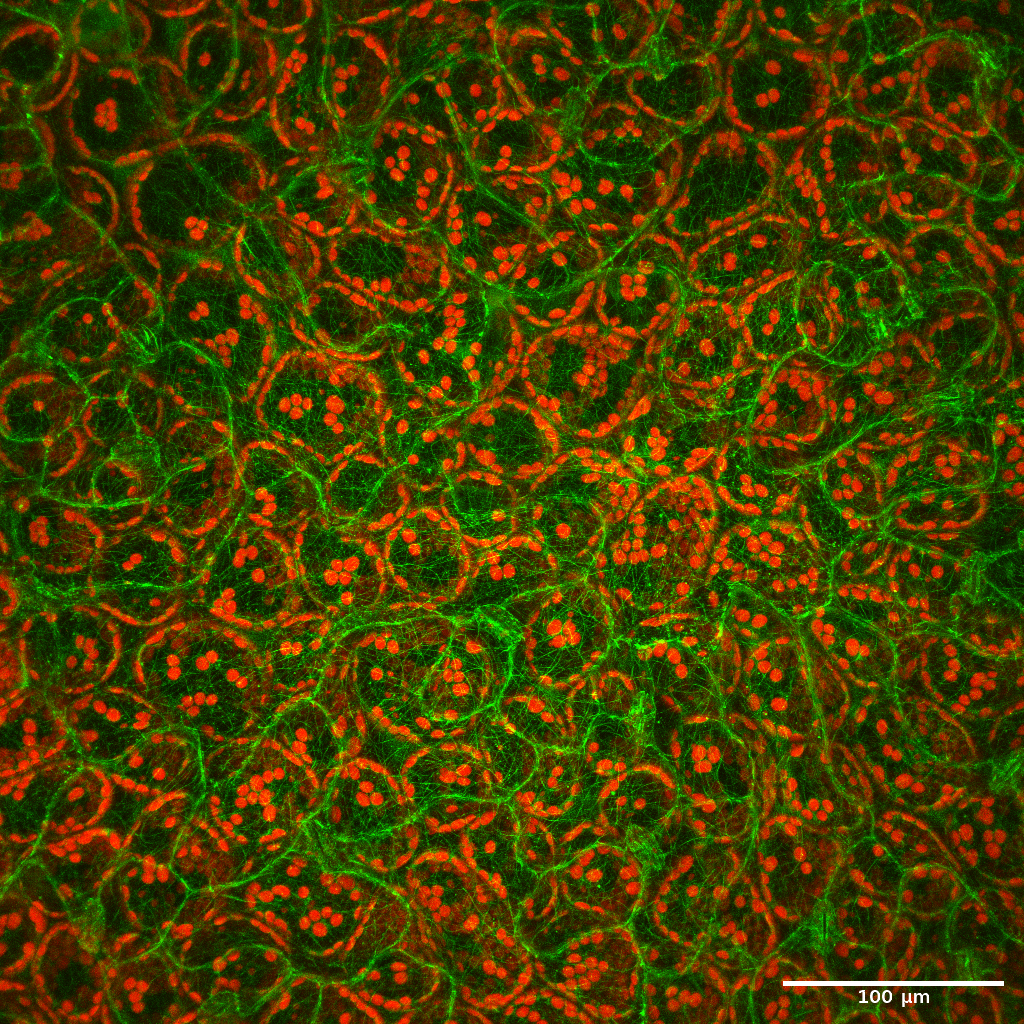

Supplement: Supplementary file 14 — Additional file 14: File S5. Pictures used in this work (raw and analyzed). [file 12915_2022_1495_MOESM14_ESM.xz › Infection/M3/T0.tif]

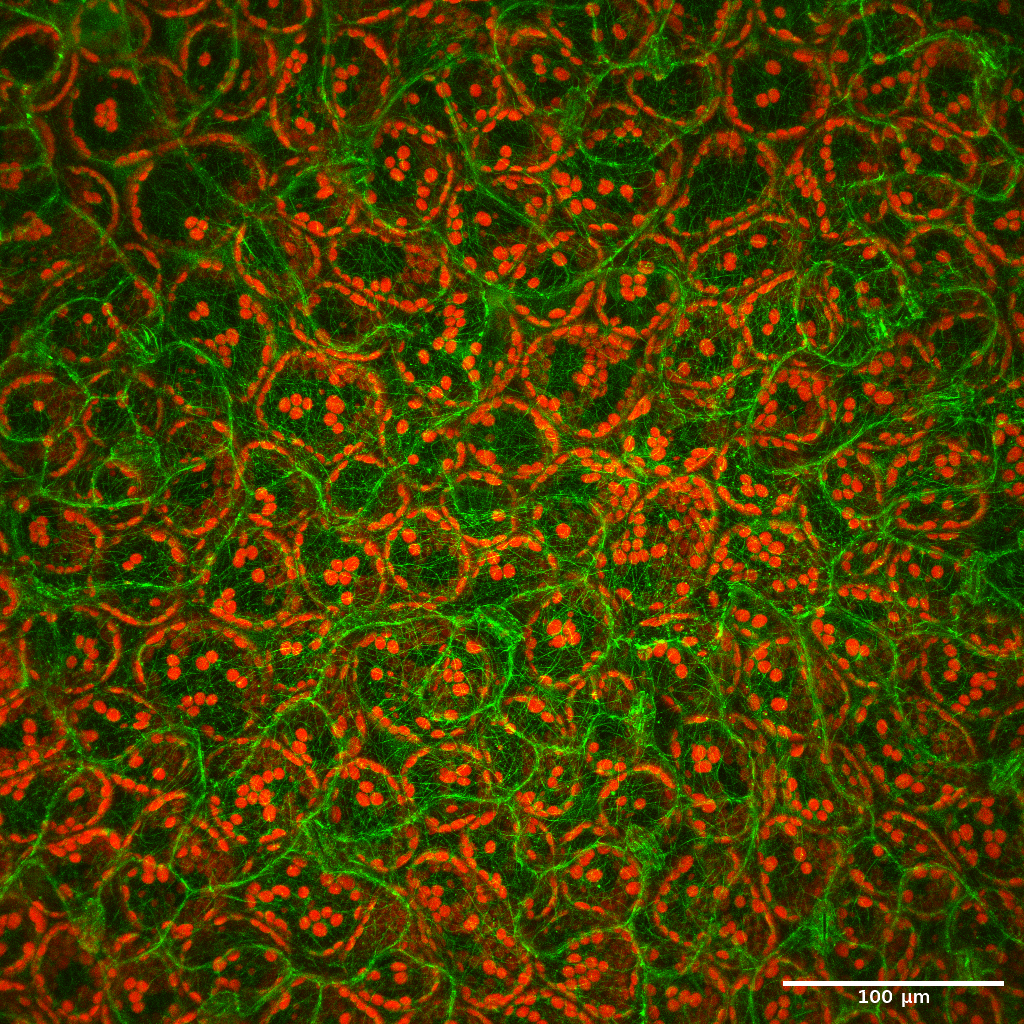

Supplement: Supplementary file 14 — Additional file 14: File S5. Pictures used in this work (raw and analyzed). [file 12915_2022_1495_MOESM14_ESM.xz › Infection/M3/T0.png]

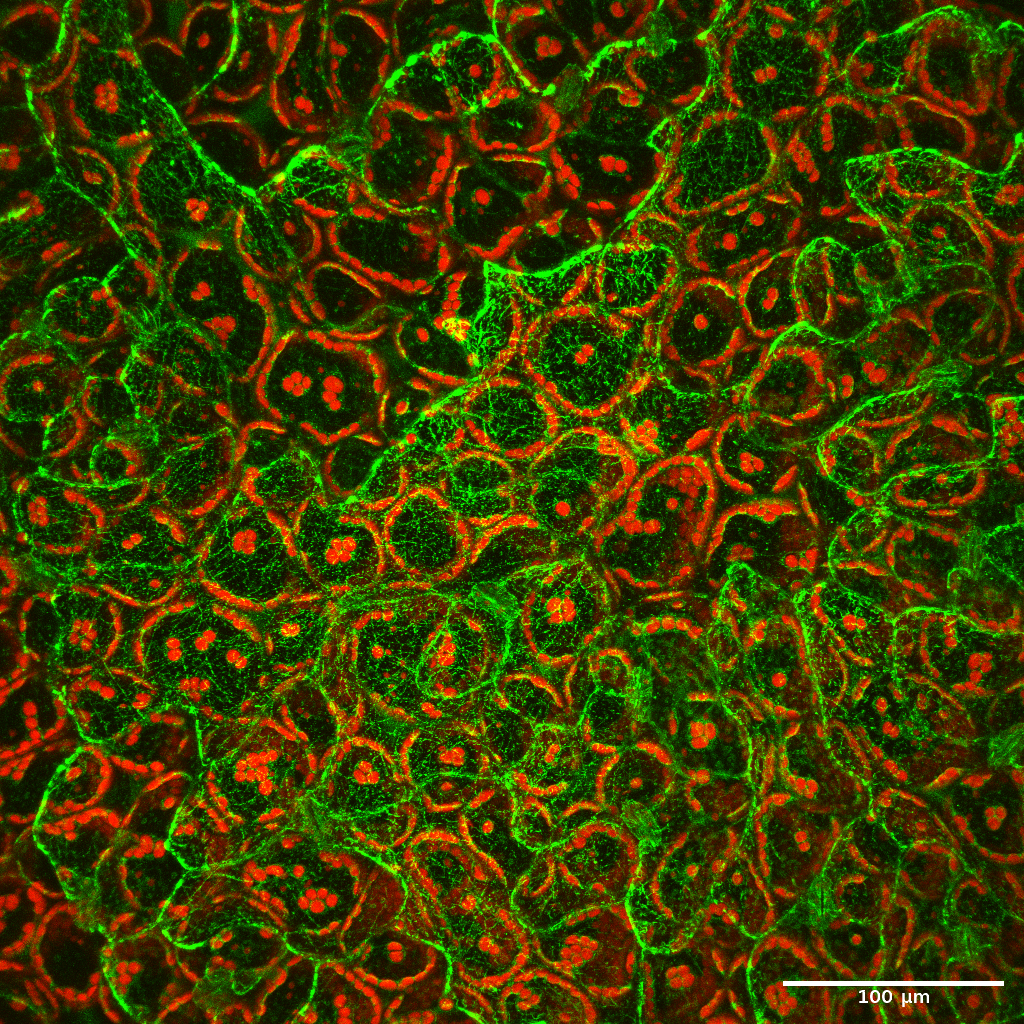

Supplement: Supplementary file 14 — Additional file 14: File S5. Pictures used in this work (raw and analyzed). [file 12915_2022_1495_MOESM14_ESM.xz › Infection/M3/T2.png]

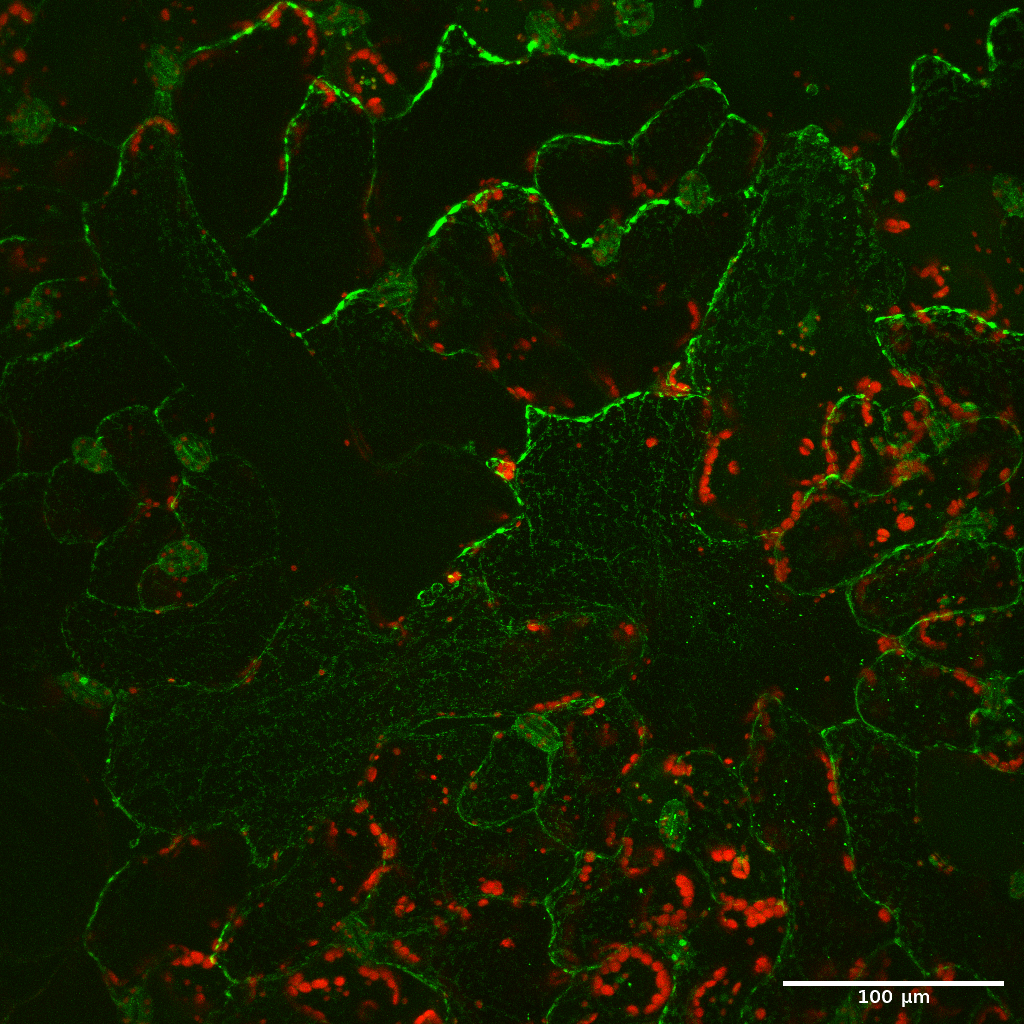

Supplement: Supplementary file 14 — Additional file 14: File S5. Pictures used in this work (raw and analyzed). [file 12915_2022_1495_MOESM14_ESM.xz › Infection/M3/T6.png]

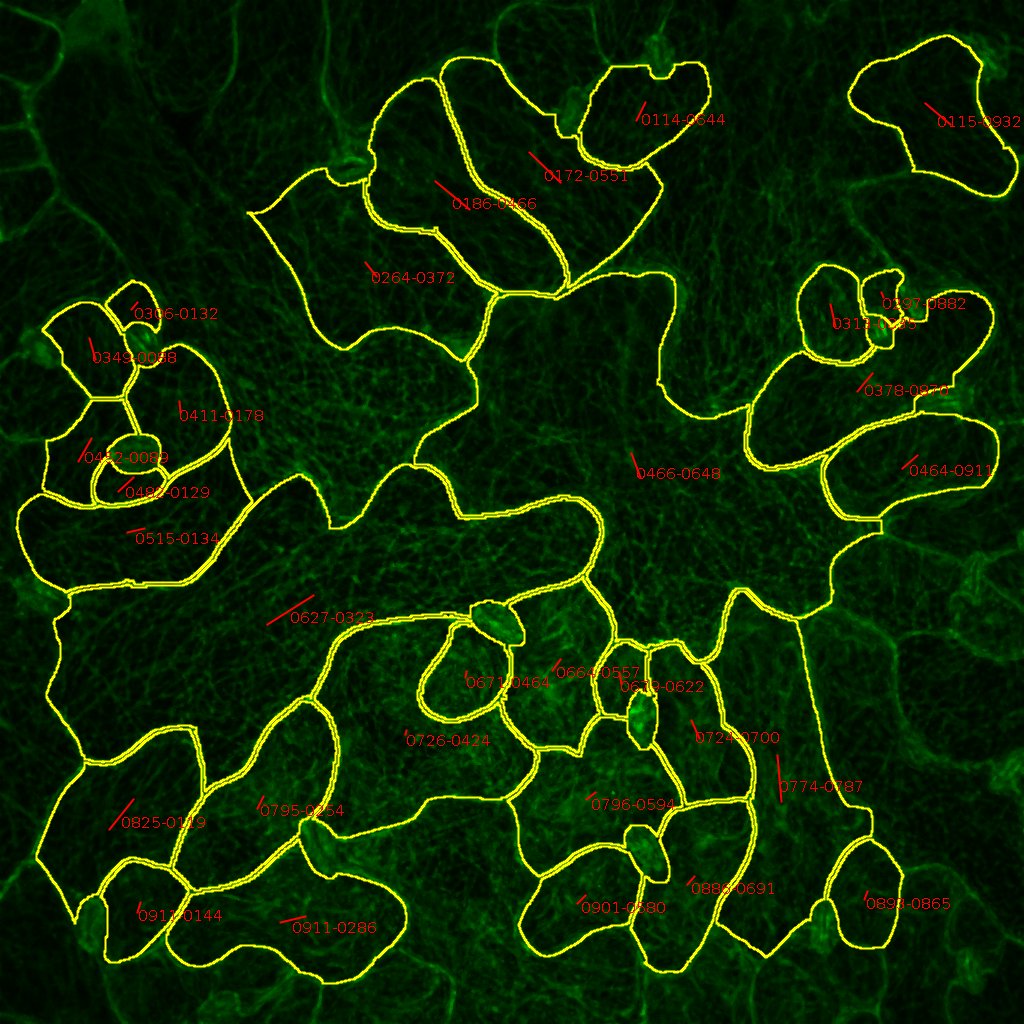

Supplement: Supplementary file 14 — Additional file 14: File S5. Pictures used in this work (raw and analyzed). [file 12915_2022_1495_MOESM14_ESM.xz › Infection/M3/24hpi/T0_fib.jpg]

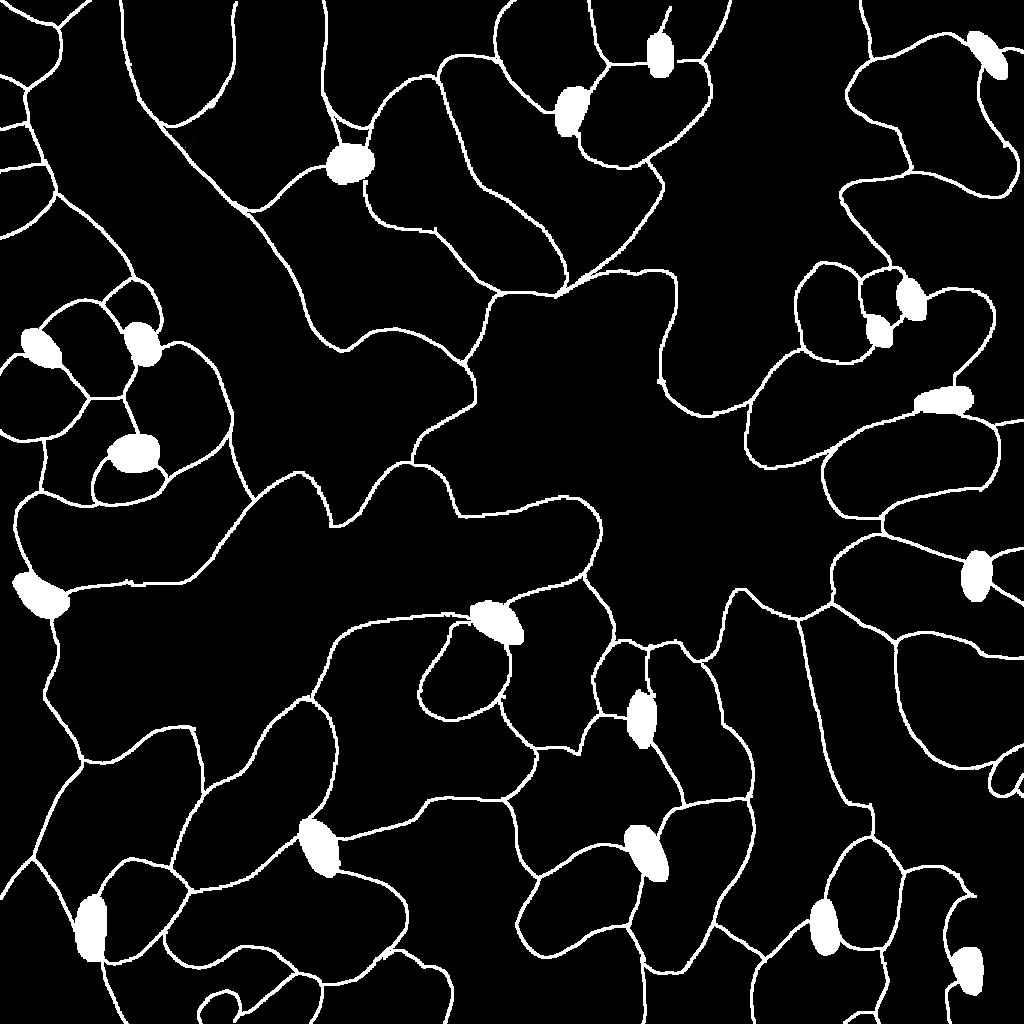

Supplement: Supplementary file 14 — Additional file 14: File S5. Pictures used in this work (raw and analyzed). [file 12915_2022_1495_MOESM14_ESM.xz › Infection/M3/24hpi/mask_checked.tif]

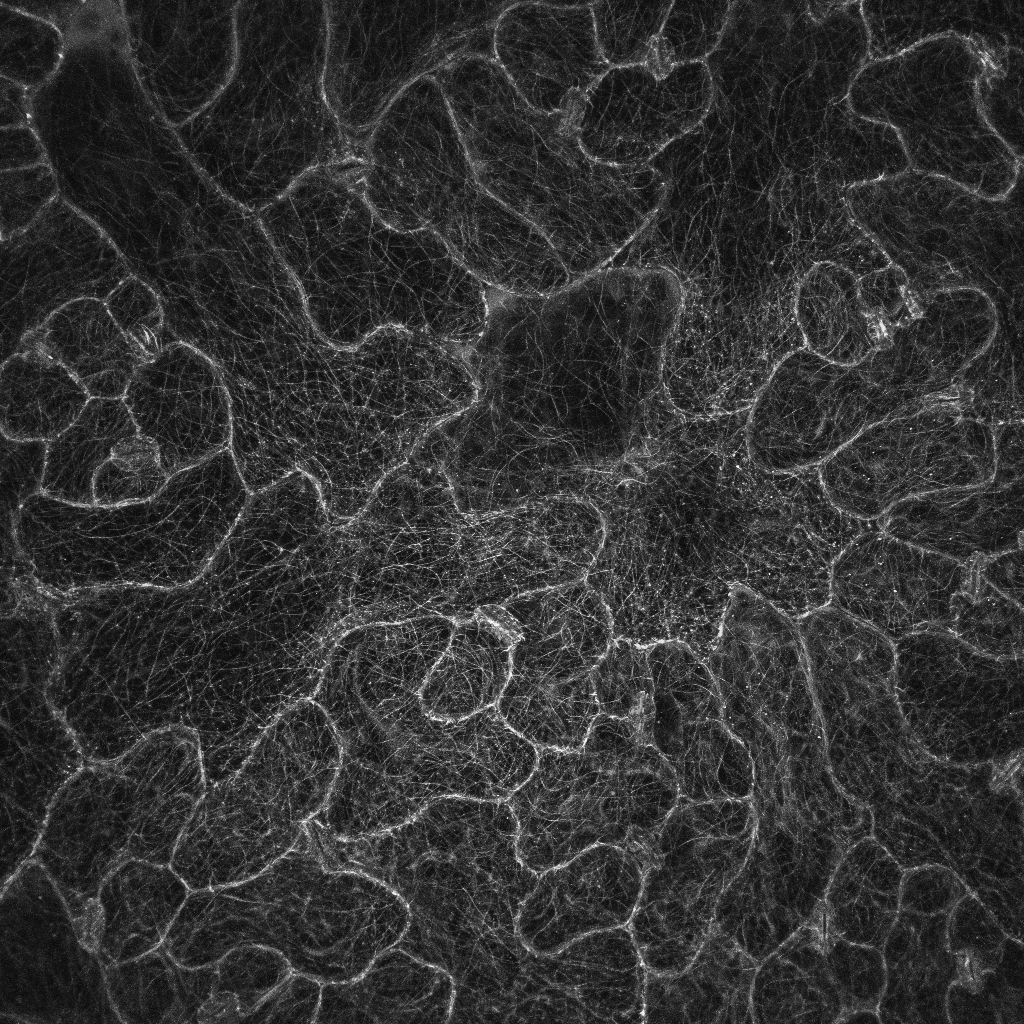

Supplement: Supplementary file 14 — Additional file 14: File S5. Pictures used in this work (raw and analyzed). [file 12915_2022_1495_MOESM14_ESM.xz › Infection/M3/24hpi/grey.tif]

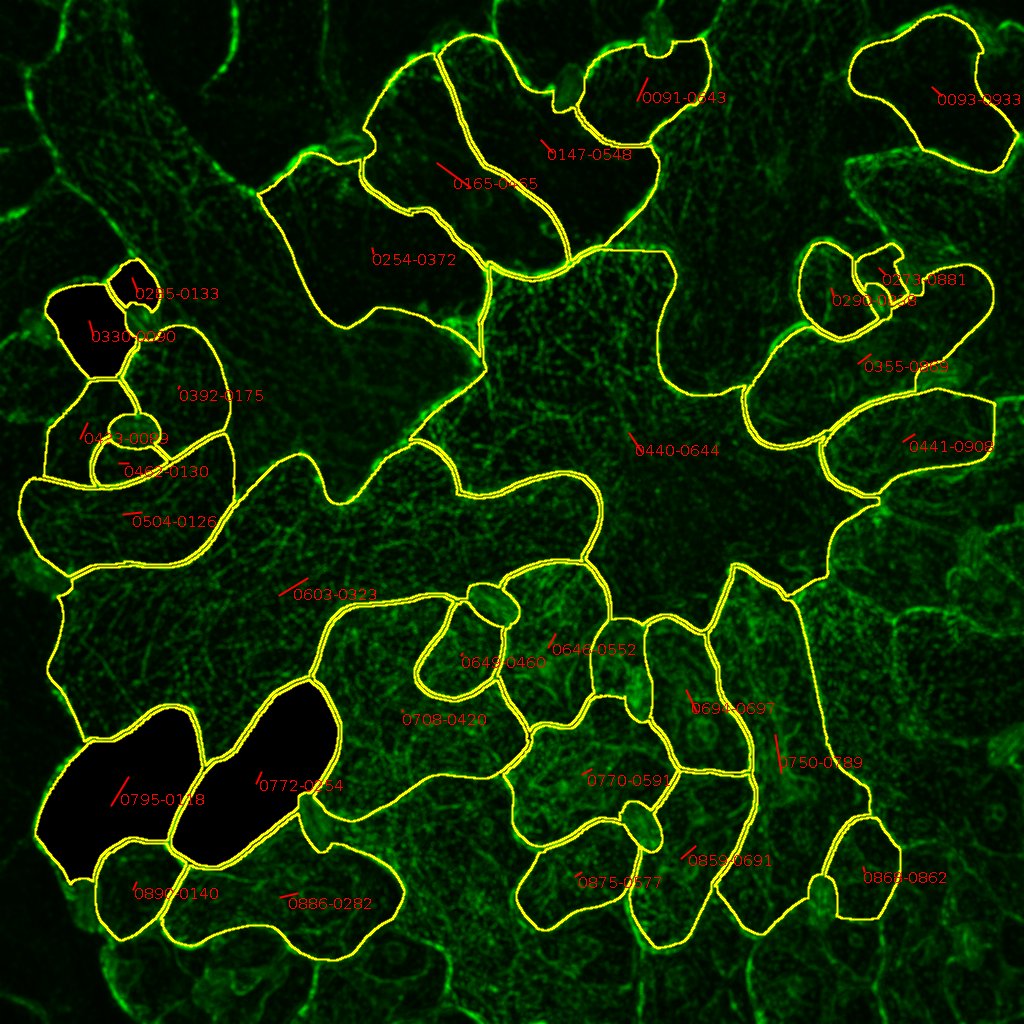

Supplement: Supplementary file 14 — Additional file 14: File S5. Pictures used in this work (raw and analyzed). [file 12915_2022_1495_MOESM14_ESM.xz › Infection/M3/26hpi/T1_fib.jpg]

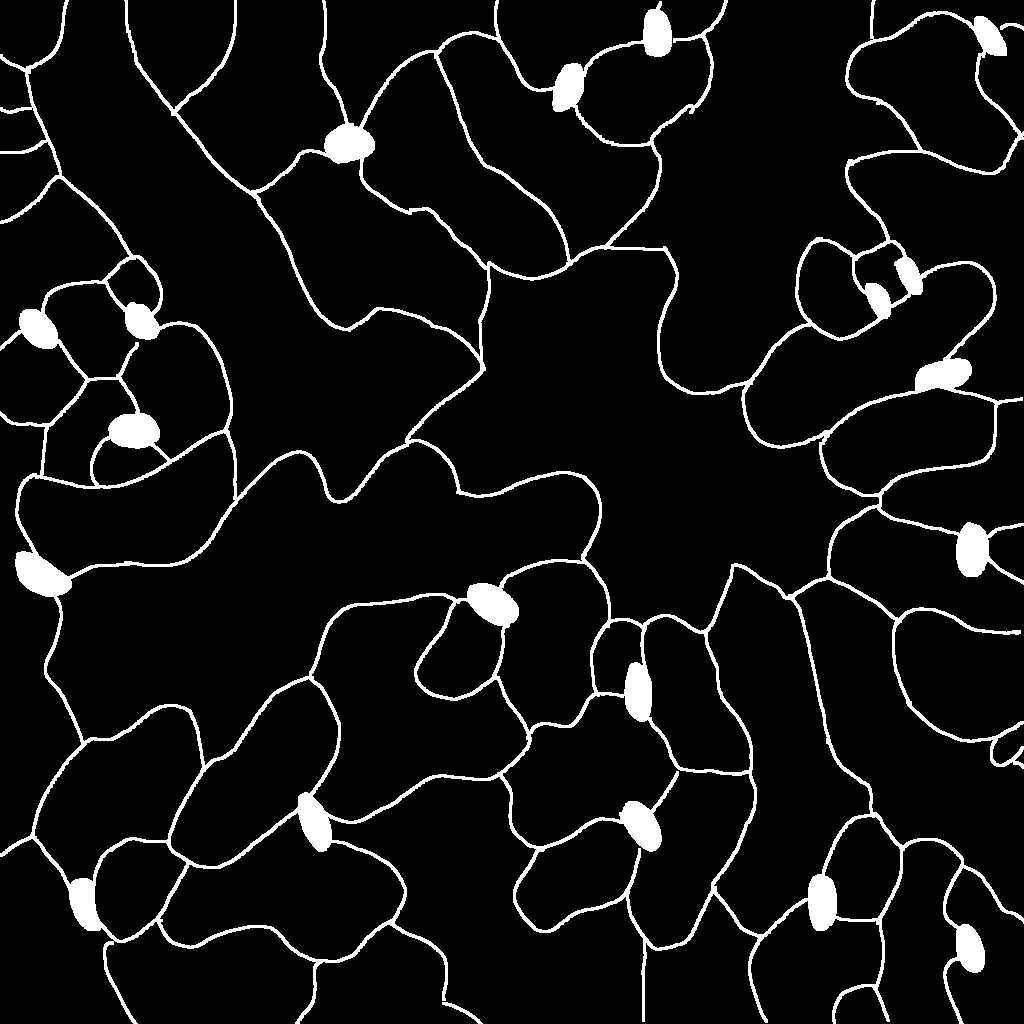

Supplement: Supplementary file 14 — Additional file 14: File S5. Pictures used in this work (raw and analyzed). [file 12915_2022_1495_MOESM14_ESM.xz › Infection/M3/26hpi/mask_checked.tif]

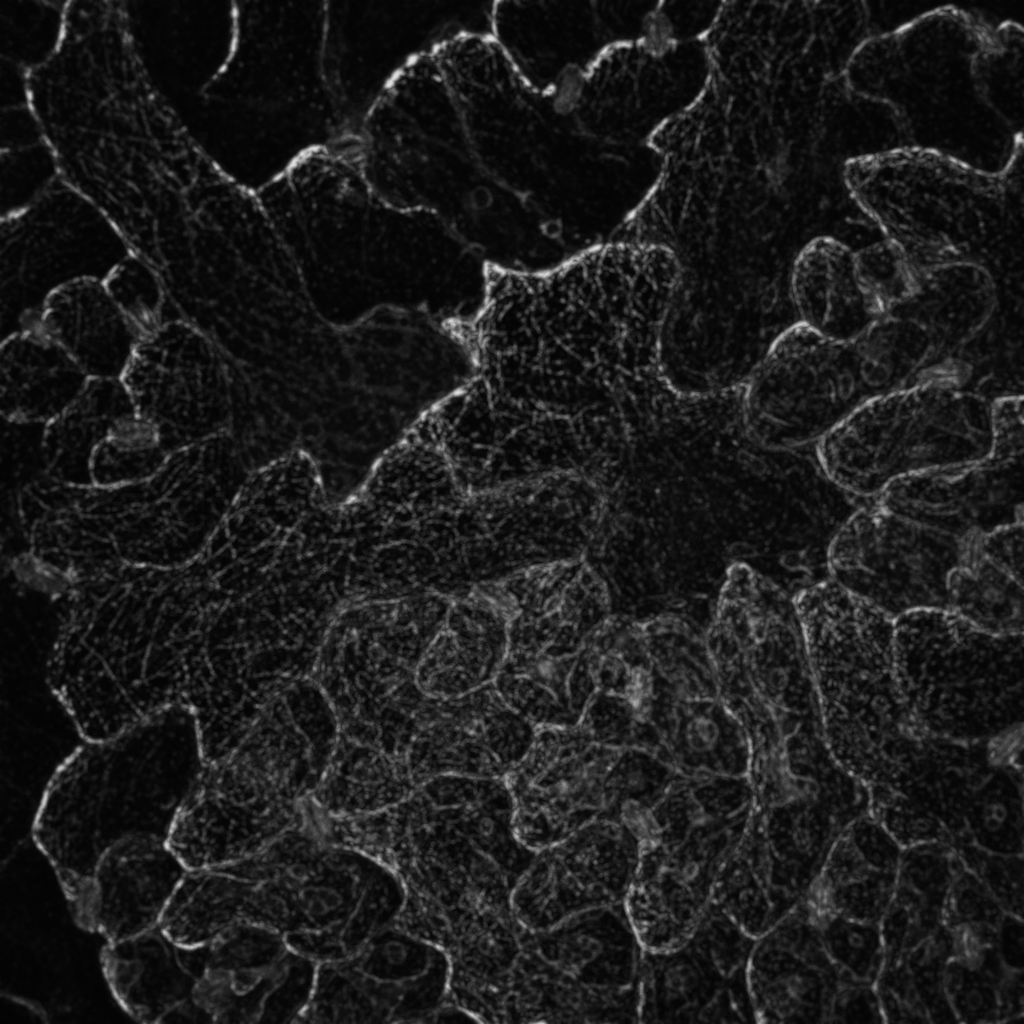

Supplement: Supplementary file 14 — Additional file 14: File S5. Pictures used in this work (raw and analyzed). [file 12915_2022_1495_MOESM14_ESM.xz › Infection/M3/26hpi/grey.tif]

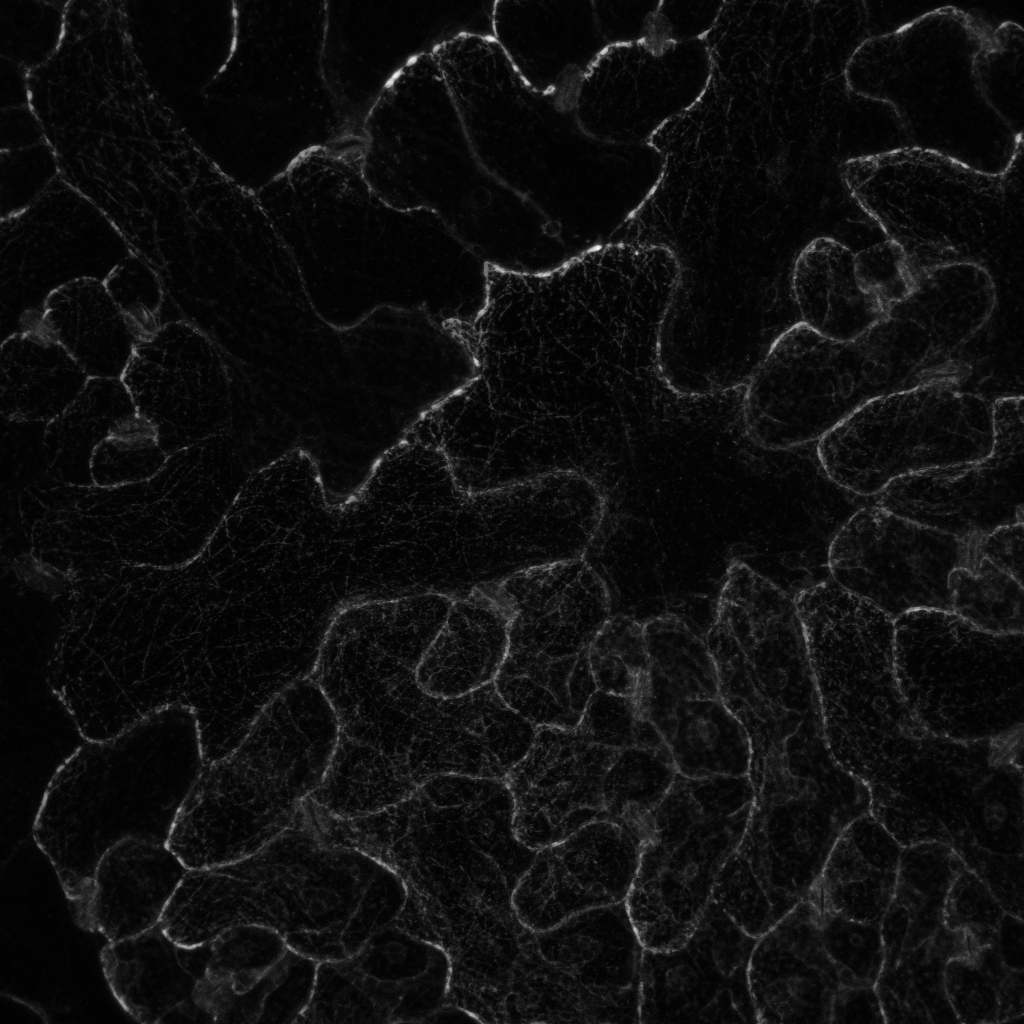

Supplement: Supplementary file 14 — Additional file 14: File S5. Pictures used in this work (raw and analyzed). [file 12915_2022_1495_MOESM14_ESM.xz › Infection/M3/KLT_T0_T1/T1.tif]

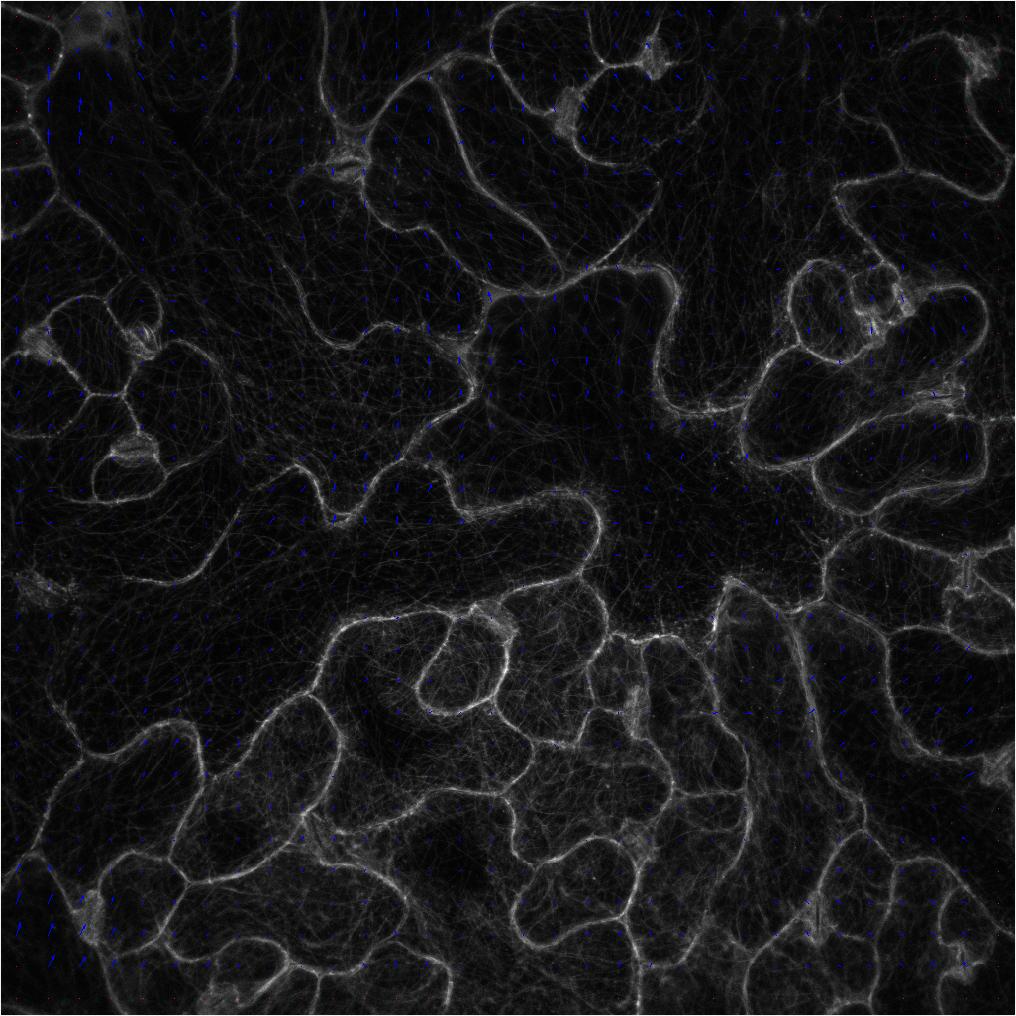

Supplement: Supplementary file 14 — Additional file 14: File S5. Pictures used in this work (raw and analyzed). [file 12915_2022_1495_MOESM14_ESM.xz › Infection/M3/KLT_T0_T1/res.jpg]

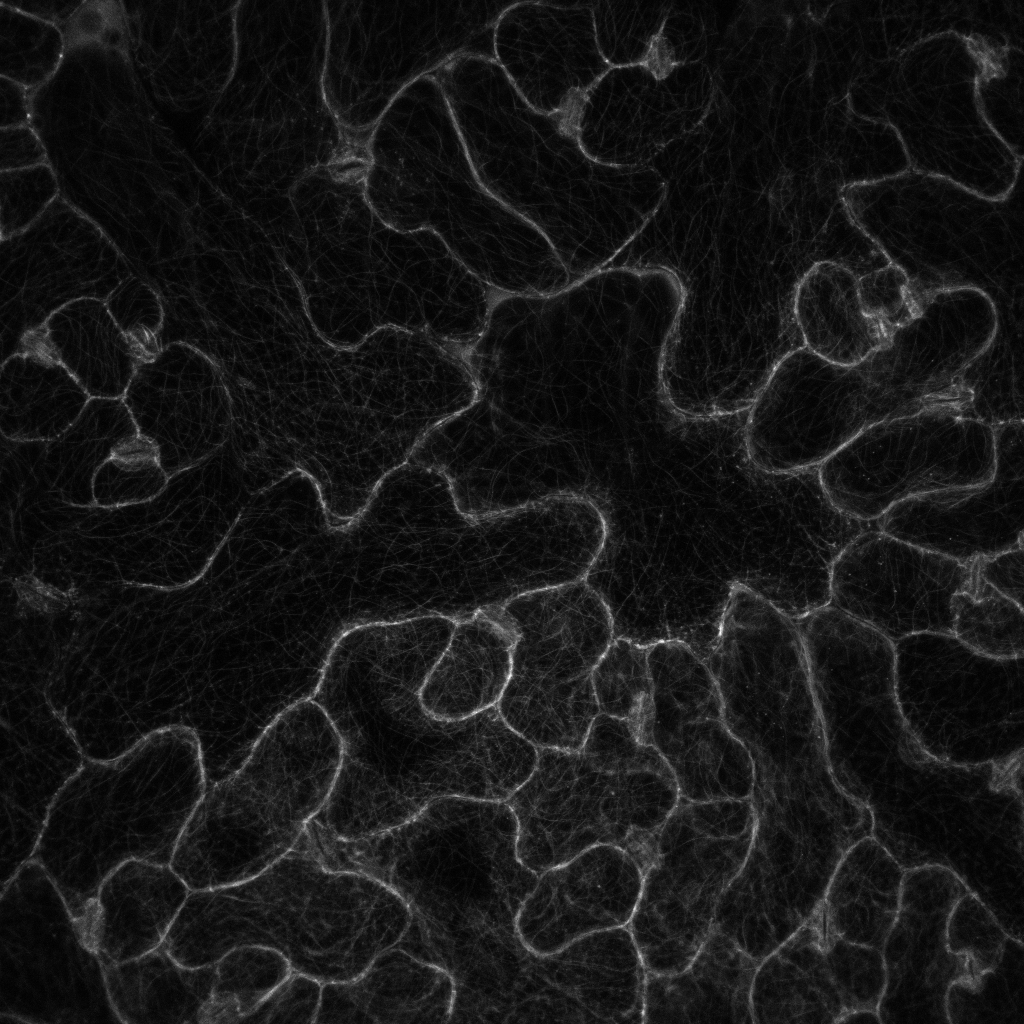

Supplement: Supplementary file 14 — Additional file 14: File S5. Pictures used in this work (raw and analyzed). [file 12915_2022_1495_MOESM14_ESM.xz › Infection/M3/KLT_T0_T1/T0.tif]

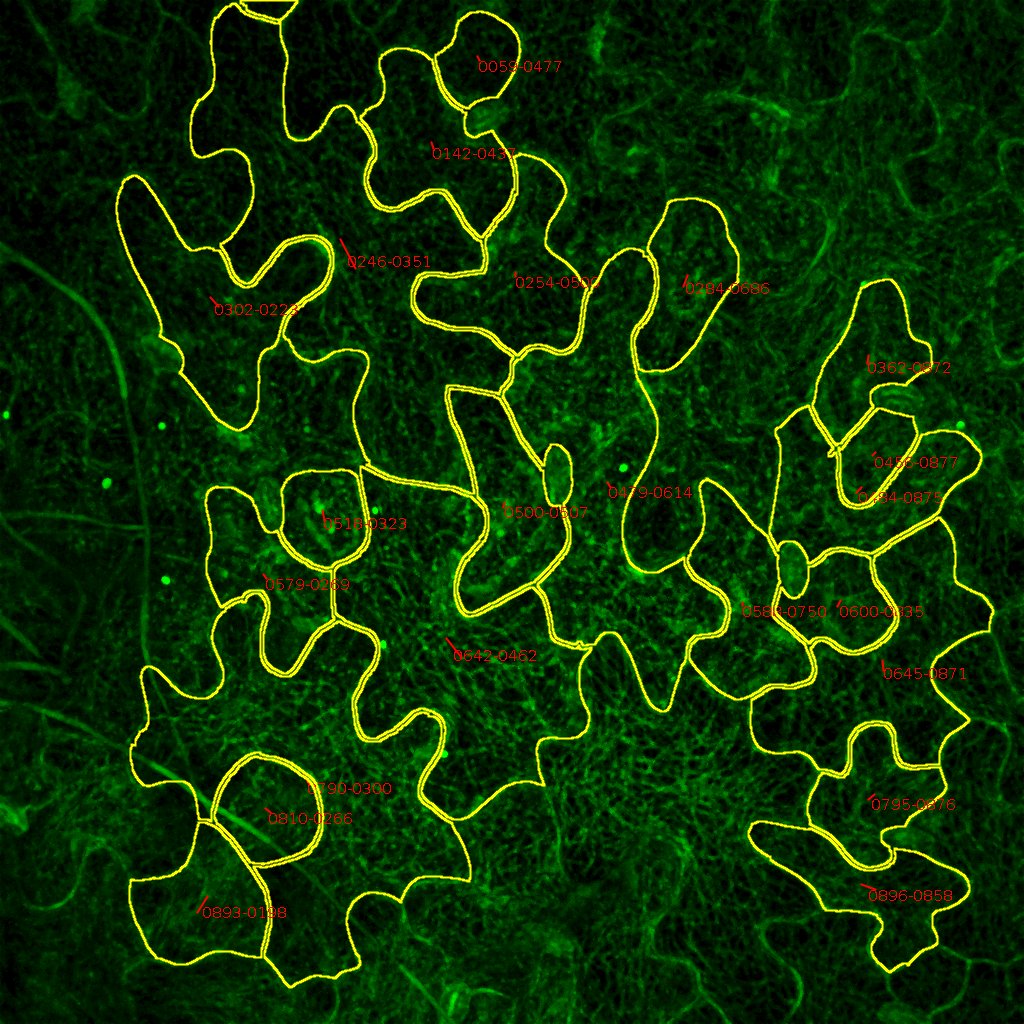

Supplement: Supplementary file 14 — Additional file 14: File S5. Pictures used in this work (raw and analyzed). [file 12915_2022_1495_MOESM14_ESM.xz › Infection/transept/Z1/24hpi/T0_fib.jpg]

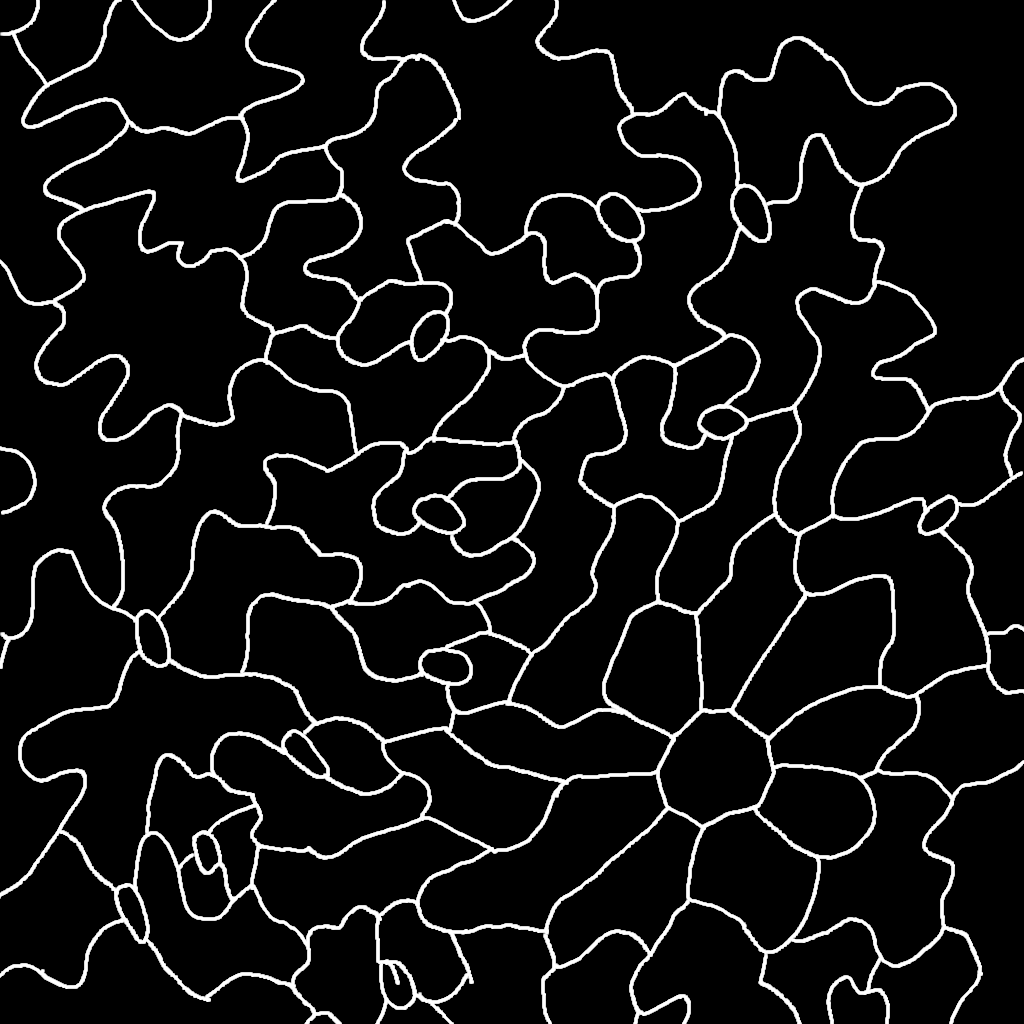

Supplement: Supplementary file 14 — Additional file 14: File S5. Pictures used in this work (raw and analyzed). [file 12915_2022_1495_MOESM14_ESM.xz › Infection/transept/Z2/24hpi/mask_checked.tif]

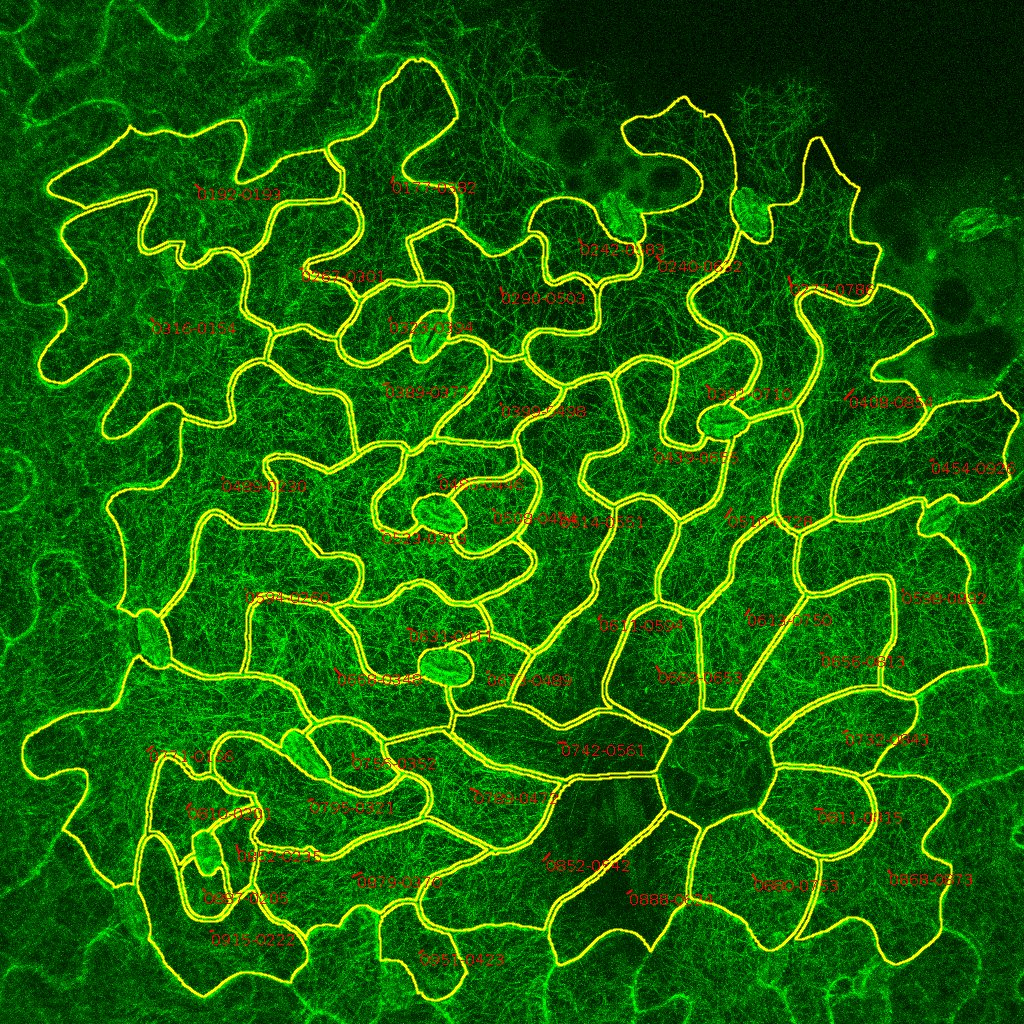

Supplement: Supplementary file 14 — Additional file 14: File S5. Pictures used in this work (raw and analyzed). [file 12915_2022_1495_MOESM14_ESM.xz › Infection/transept/Z2/24hpi/T0_fib.jpg]

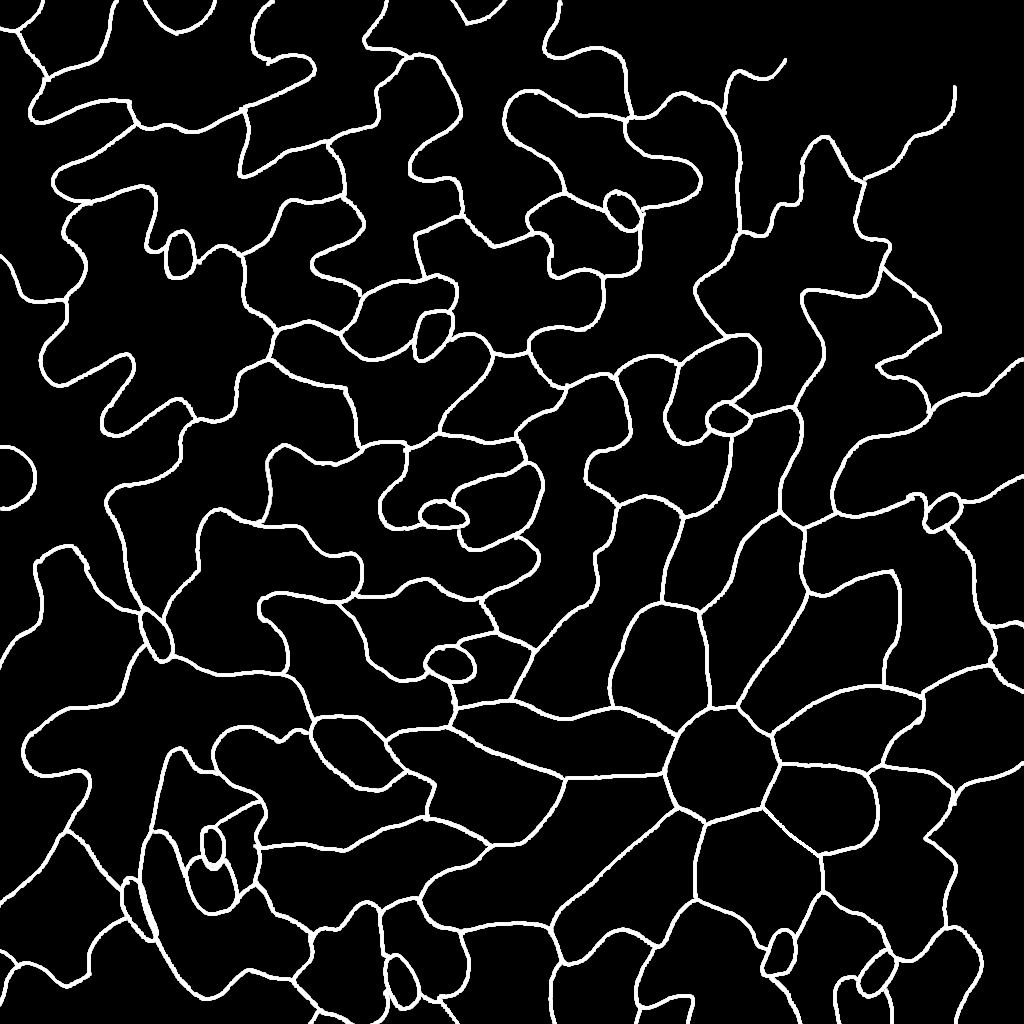

Supplement: Supplementary file 14 — Additional file 14: File S5. Pictures used in this work (raw and analyzed). [file 12915_2022_1495_MOESM14_ESM.xz › Infection/transept/Z2/26hpi/mask_checked.tif]

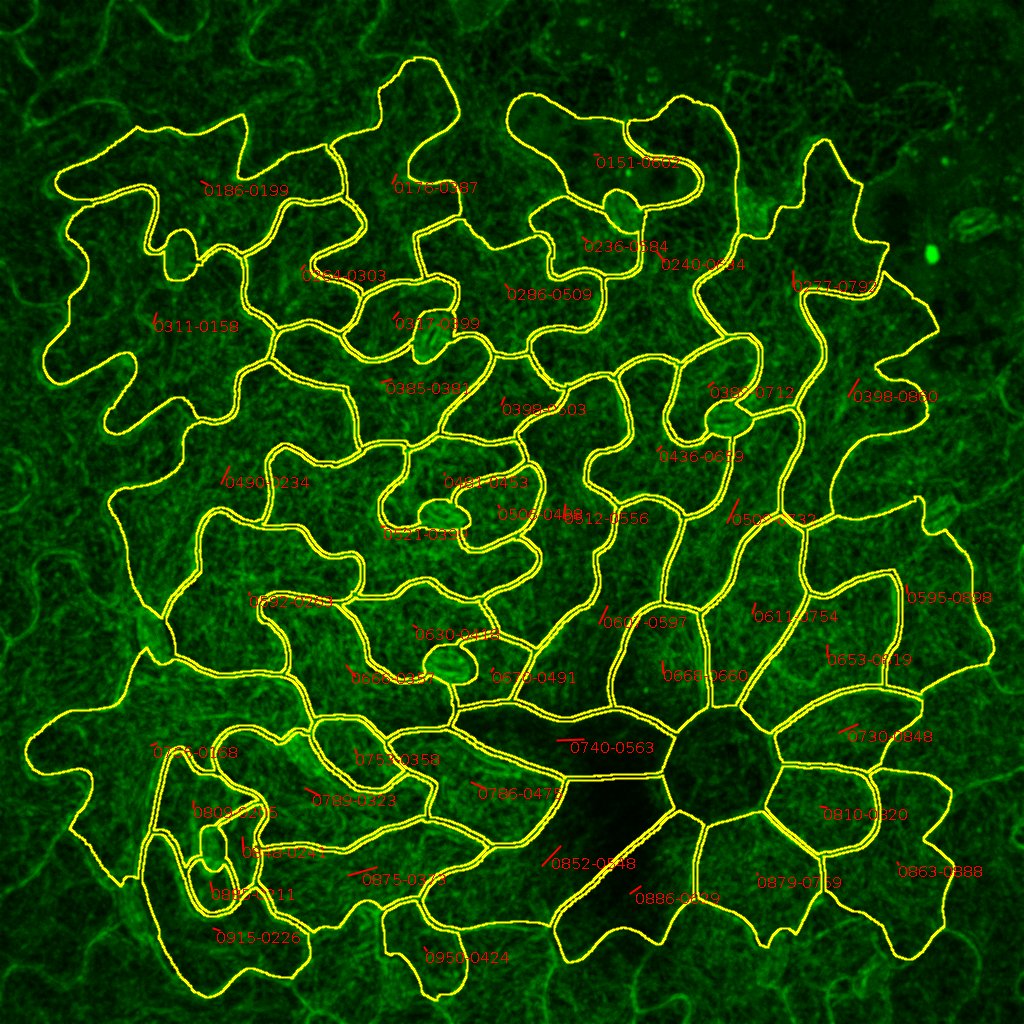

Supplement: Supplementary file 14 — Additional file 14: File S5. Pictures used in this work (raw and analyzed). [file 12915_2022_1495_MOESM14_ESM.xz › Infection/transept/Z2/26hpi/T2_fib.jpg]

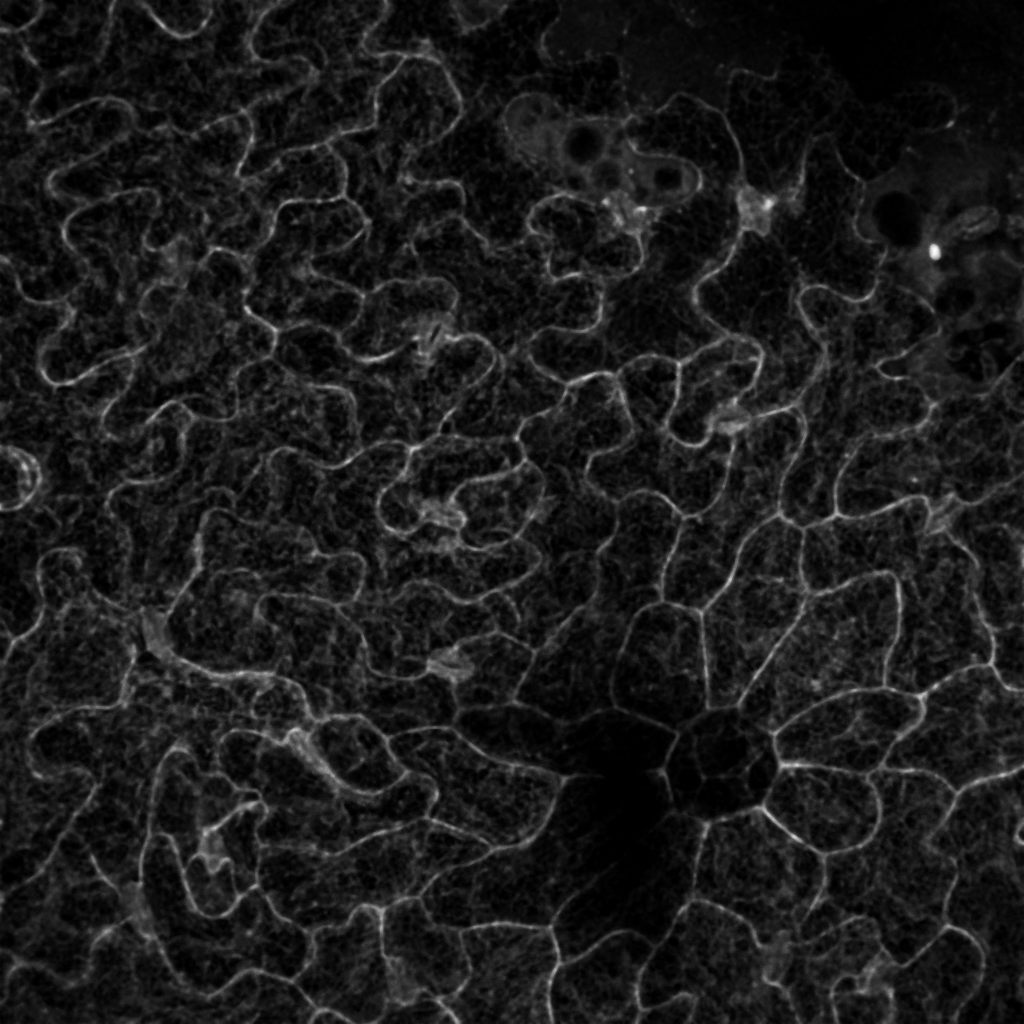

Supplement: Supplementary file 14 — Additional file 14: File S5. Pictures used in this work (raw and analyzed). [file 12915_2022_1495_MOESM14_ESM.xz › Infection/transept/Z2/KLT_T0_T2/T1.tif]

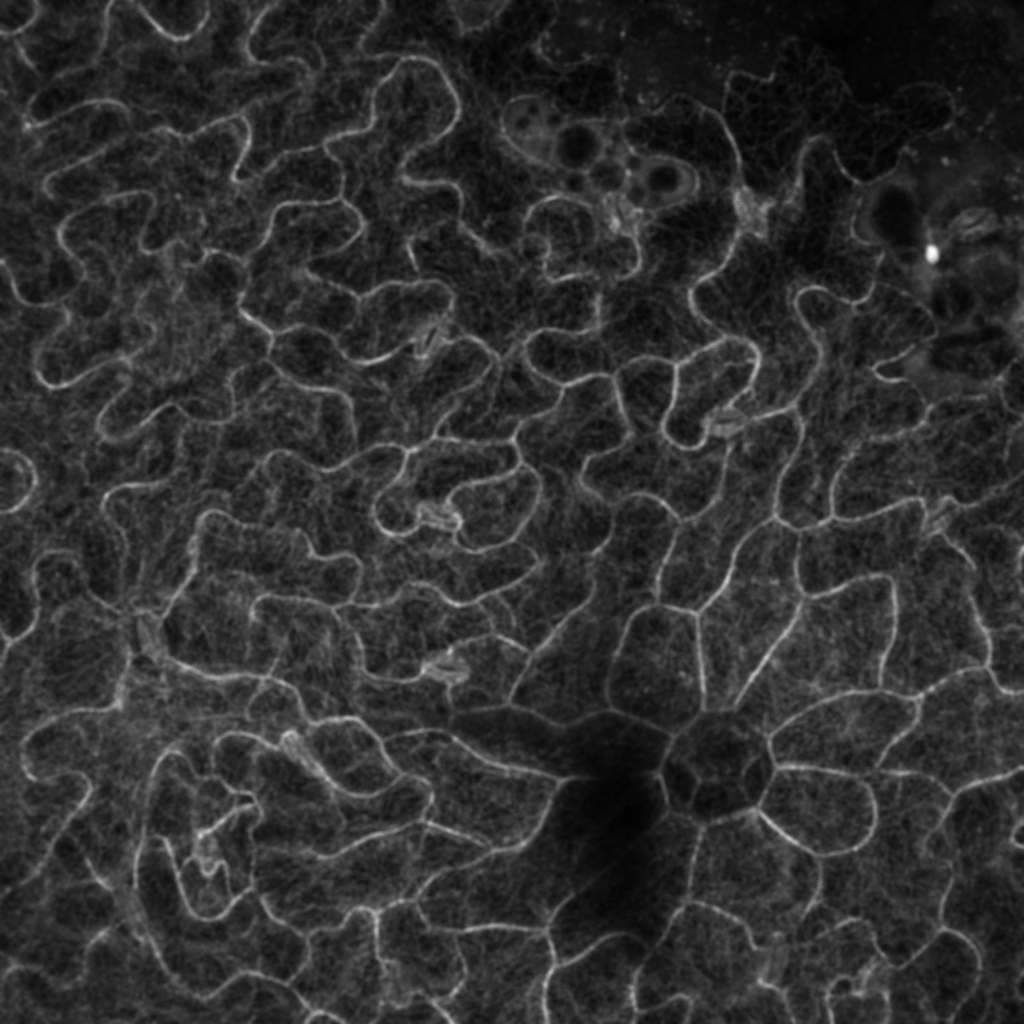

Supplement: Supplementary file 14 — Additional file 14: File S5. Pictures used in this work (raw and analyzed). [file 12915_2022_1495_MOESM14_ESM.xz › Infection/transept/Z2/KLT_T0_T2/T0.tif]

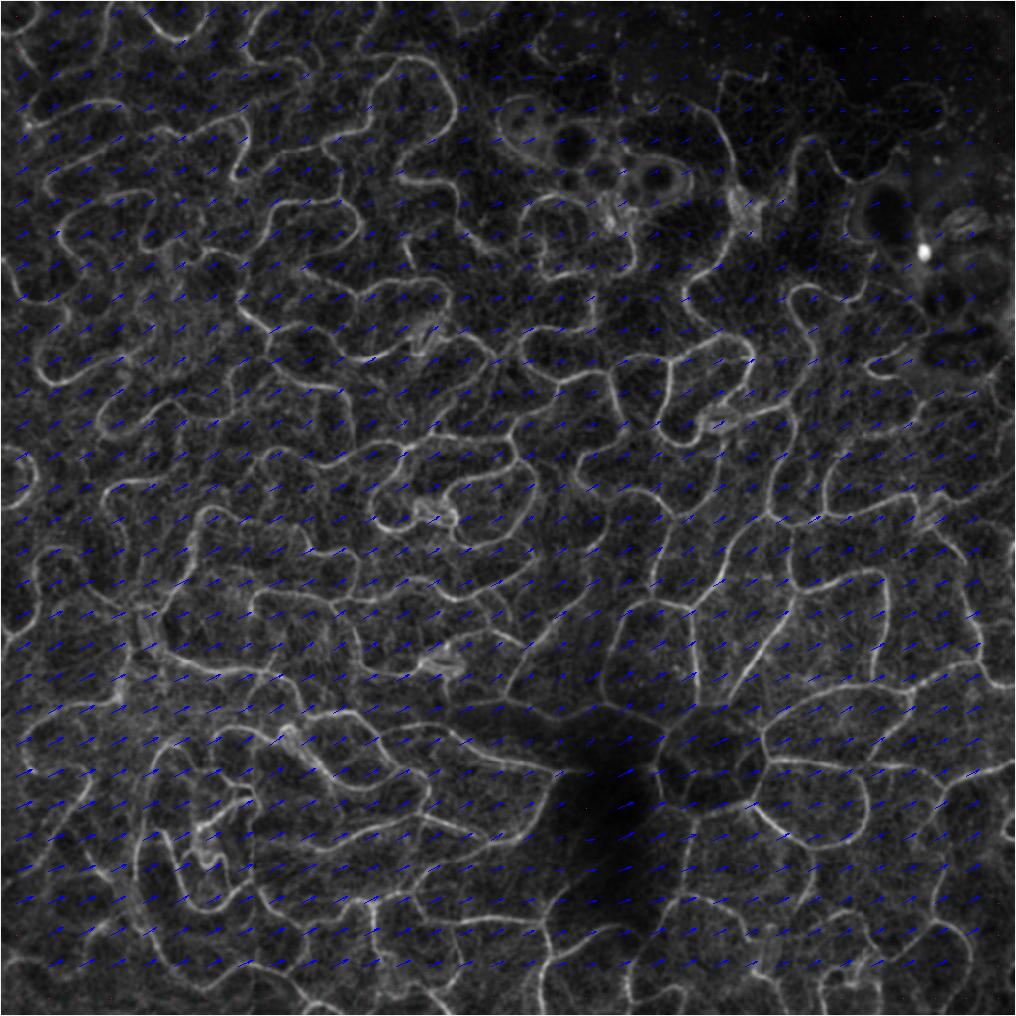

Supplement: Supplementary file 14 — Additional file 14: File S5. Pictures used in this work (raw and analyzed). [file 12915_2022_1495_MOESM14_ESM.xz › Infection/transept/Z2/KLT_T0_T2/test.jpg]
